# Similar, but not the same: multiomics comparison of human valve interstitial cells and osteoblast osteogenic differentiation expanded with an estimation of data-dependent and data-independent PASEF proteomics

**DOI:** 10.1093/gigascience/giae110

**Published:** 2025-01-11

**Authors:** Arseniy Lobov, Polina Kuchur, Nadezhda Boyarskaya, Daria Perepletchikova, Ivan Taraskin, Andrei Ivashkin, Daria Kostina, Irina Khvorova, Vladimir Uspensky, Egor Repkin, Evgeny Denisov, Tatiana Gerashchenko, Rashid Tikhilov, Svetlana Bozhkova, Vitaly Karelkin, Chunli Wang, Kang Xu, Anna Malashicheva

**Affiliations:** Laboratory of Regenerative Biomedicine, Institute of Cytology Russian Academy of Science, St. Petersburg, 194064, Russia; Laboratory of Regenerative Biomedicine, Institute of Cytology Russian Academy of Science, St. Petersburg, 194064, Russia; Laboratory of Regenerative Biomedicine, Institute of Cytology Russian Academy of Science, St. Petersburg, 194064, Russia; Almazov National Medical Research Centre, St. Petersburg, 197341, Russia; Laboratory of Regenerative Biomedicine, Institute of Cytology Russian Academy of Science, St. Petersburg, 194064, Russia; Laboratory of Regenerative Biomedicine, Institute of Cytology Russian Academy of Science, St. Petersburg, 194064, Russia; Laboratory of Regenerative Biomedicine, Institute of Cytology Russian Academy of Science, St. Petersburg, 194064, Russia; Laboratory of Regenerative Biomedicine, Institute of Cytology Russian Academy of Science, St. Petersburg, 194064, Russia; Laboratory of Regenerative Biomedicine, Institute of Cytology Russian Academy of Science, St. Petersburg, 194064, Russia; Almazov National Medical Research Centre, St. Petersburg, 197341, Russia; Centre for Molecular and Cell Technologies, St. Petersburg State University, St. Petersburg, 199034, Russia; Laboratory of Cancer Progression Biology, Cancer Research Institute, Tomsk National Research Medical Center, Russian Academy of Sciences, Tomsk, 634050, Russia; Laboratory of Cancer Progression Biology, Cancer Research Institute, Tomsk National Research Medical Center, Russian Academy of Sciences, Tomsk, 634050, Russia; Vreden National Medical Research Center of Traumatology and Orthopedics, St. Petersburg, 195427, Russia; Vreden National Medical Research Center of Traumatology and Orthopedics, St. Petersburg, 195427, Russia; Vreden National Medical Research Center of Traumatology and Orthopedics, St. Petersburg, 195427, Russia; School of Pharmacy, Hubei University of Chinese Medicine, Wuhan, 430065, China; School of Pharmacy, Hubei University of Chinese Medicine, Wuhan, 430065, China; Laboratory of Regenerative Biomedicine, Institute of Cytology Russian Academy of Science, St. Petersburg, 194064, Russia

**Keywords:** timsTOF Pro, Data-Independent Acquisition, DIA-PASEF, calcific aortic valve disease (CAVD), valve interstitial cells, osteogenic differentiation

## Abstract

Osteogenic differentiation is crucial in normal bone formation and pathological calcification, such as calcific aortic valve disease (CAVD). Understanding the proteomic and transcriptomic landscapes underlying this differentiation can unveil potential therapeutic targets for CAVD. In this study, we employed RNA sequencing transcriptomics and proteomics on a timsTOF Pro platform to explore the multiomics profiles of valve interstitial cells (VICs) and osteoblasts during osteogenic differentiation. For proteomics, we utilized 3 data acquisition/analysis techniques: data-dependent acquisition (DDA)–parallel accumulation serial fragmentation (PASEF) and data-independent acquisition (DIA)–PASEF with a classic library-based (DIA) and machine learning–based library-free search (DIA-ML). Using RNA sequencing data as a biological reference, we compared these 3 analytical techniques in the context of actual biological experiments. We use this comprehensive dataset to reveal distinct proteomic and transcriptomic profiles between VICs and osteoblasts, highlighting specific biological processes in their osteogenic differentiation pathways. The study identified potential therapeutic targets specific for VICs osteogenic differentiation in CAVD, including the MAOA and ERK1/2 pathway. From a technical perspective, we found that DIA-based methods demonstrate even higher superiority against DDA for more sophisticated human primary cell cultures than it was shown before on HeLa samples. While the classic library-based DIA approach has proved to be a gold standard for shotgun proteomics research, the DIA-ML offers significant advantages with a relatively minor compromise in data reliability, making it the method of choice for routine proteomics.

## Background

Vessels and bone tissues exhibit intricate interconnections, where vessels provide a structural framework for bone formation and play a pivotal role in governing bone development and repair [1]. This interrelation clarifies why, in pathological conditions, vascular tissues may undergo calcification. Calcifications can manifest in diverse forms and mechanisms throughout the body, significantly elevating health risks irrespective of location. Notably, meta-analyses have indicated that calcification in any arterial wall is linked to a 3- to 4-fold increase in mortality risk and cardiovascular events [2].

Calcific aortic valve disease (CAVD) or calcific aortic valve stenosis is one of the most dangerous forms of vascular calcification. CAVD is a gradual condition that initiates with aortic valve thickening and gradually advances to extensive calcification. Subsequently, valve calcification in advanced stages can lead to potentially life-threatening circulatory disorders. The prevalence of CAVD has exhibited substantial growth over time. Recent statistics indicate a rise in incident cases of CAVD globally, from 130,822 in 1990 to 589,638 in 2019 [3]. During this period, CAVD witnessed the highest surge in death rates and prevalence among the three primary valvular diseases, including rheumatic heart disease, mitral regurgitation, and CAVD [4]. Over the past three decades, many Western nations have witnessed a prevalence increase of over 10% in CAVD [4]. While comprehensive data are lacking for numerous resource-poor countries, the global trend underscores a universal concern [4, 5]. The existing treatment for CAVD is based on surgical valve replacement using either a bio- or mechanical prosthesis, necessitating replacement every 5–15 years. It is vital to develop therapeutic strategies that can mitigate the progression of CAVD urgently.

A fundamental lack of understanding regarding the pathogenesis of CAVD stands as a critical barrier to therapeutic interventions. The endothelium is believed to be significant in initiating CAVD [6], the osteogenic transformation of resident valve interstitial cells (VICs) is central to disease progression and valve calcification [5, 7].

While there are many high-quality studies on the molecular mechanisms behind VICs osteogenic differentiation [8], there are still gaps in our understanding. To develop potential therapies, it is essential to understand the differences between VICs osteogenic transdifferentiation and normal osteogenic differentiation. Although it is generally assumed that osteogenic differentiation of VICs is similar to that of osteoblasts, there is still no empirical confirmation of this assumption because of the lack of multiomics studies that describe the molecular mechanisms of adult osteoblasts differentiation and physiology [9]. Therefore, we aimed to perform the multiomics comparison of molecular mechanisms of VICs and osteoblasts osteogenic differentiation *in vitro*.

In omics, particularly in proteomics research, the depth of biological insights is constrained by the technical capabilities of omics methodologies. The field of mass spectrometry–based omics is advancing, with novel platforms for proteomics emerging regularly. This progress necessitates the development of specialized bioinformatics methods. However, given that the primary users of mass spectrometry–based omics frequently include biologists and clinicians who may not have expertise in mass spectrometry, conducting comparative studies is crucial. Such studies should elucidate how different platforms and data acquisition strategies impact the ability to address biological questions effectively.

The trapped ion mobility spectrometry coupled with time-of-flight mass spectrometry (TIMS-TOF) technology, exemplified by the timsTOF Pro platform from Bruker, represents a cutting-edge advancement in shotgun proteomics, showcasing superior performance capabilities [10]. This platform supports both data-dependent acquisition (DDA) and data-independent acquisition (DIA) strategies, offering flexibility in experimental approaches. Moreover, various bioinformatics tools are accessible for analyzing data derived from either approach. Despite numerous studies comparing these methodologies and their corresponding analysis software using standard test samples, such as HeLa cell lysate, a significant gap exists in the literature concerning their comparison in the context of addressing specific biological inquiries in biomedical studies [11–14].

To bridge biological and technical gaps in current knowledge, we conducted a comparative study on the molecular mechanisms underlying osteogenic differentiation in human osteoblasts isolated from adult femur bones and VICs from patients with CAVD. Our methodology combined transcriptomics with shotgun proteomics. To achieve the most comprehensive proteomic coverage and assess various parallel accumulation serial fragmentation (PASEF) techniques, we analyzed the same samples using DDA- and DIA-PASEF. For the DIA-PASEF data, a library-free search strategy utilizing DIA-NN was employed alongside a traditional library-based approach, constructing a spectral library from prefractionated pooled samples of each biological group under investigation.

Our findings reveal differences in molecular mechanisms of osteoblasts and VICs osteogenic differentiation, which might be used to target anti-CAVD therapy development. Specifically, we underline MAOA and ERK1/2 as fruitful targets, which are upregulated in VIC osteogenic differentiation and have been shown to have the potential for CAVD treatment before. From a technical perspective, we show that for complex samples, such as human primary cell cultures, DIA-PASEF outperforms DDA to an even greater extent than previously demonstrated with test samples. While classic DIA-PASEF with library-based data analysis appears to be a gold standard, the library-free search approach with DIA-NN proves nearly as efficient as the library-based method in recognizing differentially expressed proteins. By comparing the RNA sequencing (RNA-seq data), we observed only a minor compromise in data reliability with the DIA-NN method compared to library-based analysis.

## Methods

**Figure 1:**
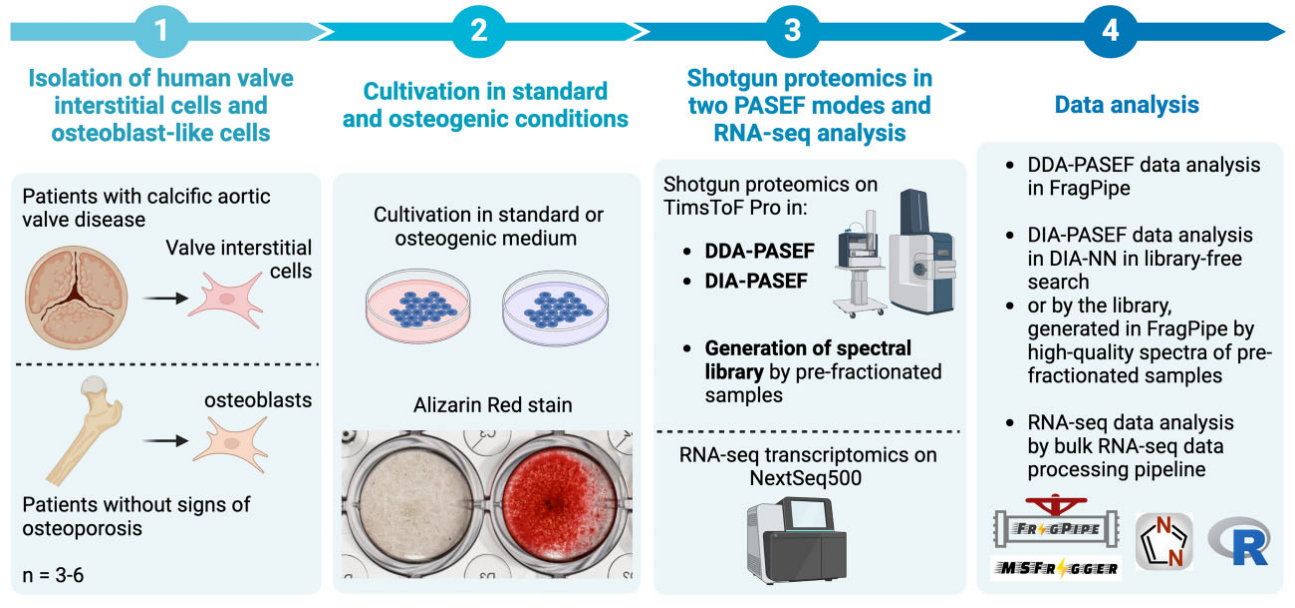
A schematic representation of the study design.

A schematic representation of the study design is provided in Fig. 1, followed by a detailed explanation in the text below.

### Cell cultures

#### Valve interstitial cell isolation

Human VICs were obtained from the calcified human aortic valves (*n* = 5), which were isolated from patients with CAVD during surgical valve replacement in the Almazov National Medical Research Centre of the Ministry of Health of the Russian Federation. The study was conducted according to the guidelines of the Declaration of Helsinki and approved by the local Ethics Committee of the Almazov Federal Medical Research Center. All patients gave informed consent. Patients with bicuspid valves were excluded.

Fragments of calcified valve were washed in phosphate-buffered saline (PBS) and treated with collagenase II (1 mg/mL; Worthington Biochemical Corporation) in PBS for 10 minutes, and then endothelial cells were scraped out. The tissues were then digested by collagenase II in a standard cultivation medium overnight at 37°C. Tissue fragments were transferred to a cultural flask.

#### Osteoblasts isolation

Human osteoblast-like cells were obtained from the fragments of the epiphysis of bone spongy tissue of femur bone (*n* = 6), harvested during surgery at Vreden National Medical Research Center of Traumatology and Orthopedics. The local Ethics Committee of Vreden National Medical Research Center of Traumatology and Orthopedics approved the clinical research protocol and followed the principles of the Declaration of Helsinki. All patients gave informed consent. Patients with osteoporosis were excluded.

Bone fragments were washed in PBS supplemented with penicillin/streptomycin 3 times. Then, the fragments were crushed into fragments up to 0.5 mm using carbide cutters. Cancellous bone was repeatedly washed in PBS until connective tissue and capillaries were removed. Then, the fragments were incubated with 0.2% collagenase type II (Worthington Biochemical Corporation) solution in PBS for 30 minutes at 37°C, washed in PBS, and transferred into 0.2% collagenase type IV solution (Worthington Biochemical Corporation) in standard cultivation media and incubated for 16 hours at 37°C. Then, fragments were transferred to a culture flask, where cells were explanted for several weeks.

#### Cell culturing

Both VICs and osteoblasts were cultured in the same conditions (37°C in 5% CO_2_) in Dulbecco’s modified Eagle’s medium (DMEM) (41,966–052, Gibco) supplemented with 15% fetal bovine serum (FBS; HyClone, GE Healthcare), 2 mM L-glutamine (Gibco), and penicillin/streptomycin (Gibco) until 70–80% confluence. The medium was changed twice a week. Cell cultures were routinely checked for mycoplasma contamination each week, according to Janetzko et al. [15] with minor modifications. Cells of 3–4 passages were used for the experiments.

#### Osteogenic differentiation

We used cells from the same donors for both transcriptomics and proteomics analysis. For both methods, cells were analyzed in standard cultivation and on day 10 after induction of osteogenic differentiation.

To induce osteogenic differentiation, we used a classic osteogenic medium, usually used for *in vitro* osteogenic differentiation: DMEM supplemented with 10% FBS, 2 mM L-glutamine, penicillin/streptomycin, 50 mg/mL ascorbic acid, 0.1 mM dexamethasone, and 10 mM β-glycerophosphate [9].

Osteogenic differentiation was verified by Alizarin red stain (stain for calcium deposition) on day 21 of osteogenic differentiation. For this purpose, in parallel with the main experiment, cells were plated in 24-well plates. After 21 days of differentiation, cells were washed with PBS, fixed in 70% ethanol for 60 minutes, washed twice with distilled water, and stained using Alizarin red solution (Sigma).

### Shotgun proteomics

#### Protein isolation

The cells were washed twice with PBS and lysed in a Petri dish with RIPA buffer (ThermoFisher Scientific) supplemented with a complete protease inhibitor cocktail (Roche). Then, the dishes were transferred to the ice for 30 minutes. Cell lysates were stored at −80°C before use.

The samples were sonicated for 10 minutes in an ultrasonic bath with ice and centrifuged (12,000 *g*, 20 minutes, 4°C). Proteins were cleaned by standard acetone precipitation (EM grade acetone; EMS). The protein pellet was resuspended in 8 M urea/50 mM ammonium bicarbonate (Sigma Aldrich) and centrifuged (12,000 *g*, 10 minutes, 4°C). The protein concentration was measured by a Qubit fluorometer (ThermoFisher Scientific) using the QuDye Protein Quantification Kit (Lumiprobe) according to the manufacturer’s recommendations.

#### In-solution digestion

In total, 20 μg of each sample was digested by trypsin. Sample volumes were equalized by the addition of 8 M urea/50 mM ammonium bicarbonate. Disulfide bonds were reduced and alkylated by incubation for 1 hour at 37°C in 5 mM DTT (Sigma Aldrich) with subsequent incubation in 15 mM iodoacetamide for 30 minutes in the dark at room temperature (Sigma Aldrich). Then, the samples were diluted with 7 volumes of 50 mM ammonium bicarbonate and incubated for 16 hours at 37°C with 400 ng trypsin (ratio 1:50; “Trypsin Gold”; Promega).

After digestion, the samples were desalted by solid phase extraction in “Stage tips,” self-made from polypropylene Vertex pipette tips (200 µL; SSIbio) filled with 4 layers of C18 reversed-phase excised from Empore 3 M C18 extraction disks according to Rappsilber et al. [16]. Desalted peptides were evaporated in Eppendorf Concentrator plus (Eppendorf) and dissolved in water/0.1% formic acid for further liquid chromatography/tandem mass spectrometry (LC-MS/MS) analysis. Each sample was analyzed in both DDA- and DIA-PASEF modes.

#### Library fractionation

To obtain a spectral library, we fractionated 4 pooled samples. For this purpose, we mixed all replicates across each biological group (VICs and osteoblasts in standard cultivation and after osteogenic differentiation). Then, 100 μg of each pooled sample was digested in-solution as described above and fractionated by the Pierce High pH Reversed-Phase Peptide Fractionation Kit (ThermoFisher Scientific) according to manufacturer recommendations. Each fraction was desalted as described above and dissolved in water/0.1% formic acid. The peptide concentration was measured by the Pierce Quantitative Fluorometric Peptide Assay (ThermoFisher Scientific) in a Cary Eclipse Fluorescence Spectrometer (Agilent) according to manufacturer recommendations.

#### High-performance liquid chromatography

Approximately 500 ng of tryptic peptides was used for shotgun proteomics analysis by nano–high-performance liquid chromatography (HPLC)–MS/MS with trapped ion mobility mass spectrometry in timsTOF (Bruker). HPLC was performed in 2-column separation mode with Acclaim PepMap 5-mm Trap Cartridge and Aurora Series separation column with nanoZero technology (C18, 25 cm × 75 µm ID, 1.6 µm C18) in gradient mode with a 400-nL/min flow rate. Phase A was water/0.1% formic acid, and phase B was acetonitrile/0.1% formic acid. The gradient was from 2% to 18% of phase B for 44 minutes, then to 25% of phase B for 11 minutes, to 37% of phase B for 5 minutes with subsequent wash with 95% of phase B for 17 minutes. The CaptiveSpray ion source was used for electrospray ionization with 1,600 V of capillary voltage, 3 L/min N_2_ flow, and 180°C source temperature. MS acquisition was performed in 2 different modes.

#### DDA-PASEF

MS/MS acquisition in DDA-PASEF mode was used to analyze experimental and fractionated samples for the spectral library. The analysis was performed by automatic DDA-PASEF mode with a 1.1-second cycle time and 10 PASEF ramps in positive polarity with the fragmentation of ions with at least 2 charges in *m/z* range from 100 to 1,700 and ion mobility range from 0.60 to 1.60 1/K0. The active exclusion was enabled with a release time of 0.4 minutes and reconsideration after a 4-fold increase in intensity. Collision energy was 20 eV for 0.6 1/K0 and 59 eV for 1.6 1/K0. Isolation width was 2 *m/z* for 700 *m/z* and 3 *m/z* for 800 *m/z*.

#### DIA-PASEF

Analysis in DIA-PASEF mode was performed in the same *m/z* and ion mobility range as DDA-PASEF. The DIA analysis was performed in positive polarity with the fragmentation of ions with at least 2 charges in *m/z* range from 100 to 1,700 and ion mobility range from 0.60 to 1.60 1/K0. Cycle time was 1.6 seconds, and it included 16 fragmentation steps with 26 *m/z* and 0.01–0.02 1/K0 step (Table 1). Each step included parallel fragmentation of 2 fragments of the spectra with a difference in 400 *m/z* and 0.3 1/K0. The collision energy was 20 eV for 0.85 1/K0 and 59 eV for 1.3 1/K0.

**Table 1:** DIA-PASEF isolation list

MS type	Cycle ID	Start IM [1/K0]	End IM [1/K0]	Start mass [*m/z*]	End mass [*m/z*]
MS1	0	—	—	—	—
PASEF	1	0.9001	1.2001	800.00	826.00
PASEF	1	0.6000	0.9001	400.00	426.00
PASEF	2	0.9201	1.2201	825.00	851.00
PASEF	2	0.6200	0.9201	425.00	451.00
PASEF	3	0.9301	1.2301	850.00	876.00
PASEF	3	0.6300	0.9301	450.00	476.00
PASEF	4	0.9500	1.2501	875.00	901.00
PASEF	4	0.6501	0.9500	475.00	501.00
PASEF	5	0.9600	1.2601	900.00	926.00
PASEF	5	0.6601	0.9600	500.00	526.00
PASEF	6	0.9800	1.2801	925.00	951.00
PASEF	6	0.6801	0.9800	525.00	551.00
PASEF	7	0.9900	1.2901	950.00	976.00
PASEF	7	0.6900	0.9900	550.00	576.00
PASEF	8	1.0101	1.3101	975.00	1,001.00
PASEF	8	0.7100	1.0101	575.00	601.00
PASEF	9	1.0201	1.3201	1,000.00	1,026.01
PASEF	9	0.7200	1.0201	600.00	626.00
PASEF	10	1.0401	1.3401	1,025.01	1,051.01
PASEF	10	0.7400	1.0401	625.00	651.00
PASEF	11	1.0601	1.3601	1,050.01	1,076.01
PASEF	11	0.7601	1.0601	650.00	676.00
PASEF	12	1.0701	1.3701	1,075.01	1,101.01
PASEF	12	0.7701	1.0701	675.00	701.00
PASEF	13	1.0901	1.3901	1,100.01	1,126.01
PASEF	13	0.7901	1.0901	700.00	726.00
PASEF	14	1.1001	1.4001	1,125.01	1,151.01
PASEF	14	0.8001	1.1001	725.00	751.00
PASEF	15	1.1201	1.4201	1,150.01	1,176.01
PASEF	15	0.8200	1.1201	750.00	776.00
PASEF	16	1.1300	1.4301	1,175.01	1,201.01
PASEF	16	0.8300	1.1300	775.00	801.00

#### Data analysis

Protein identification in all cases was performed using the same *Homo sapiens* reference proteome (UP000005640, downloaded 27.06.2022) with contaminations included.

Analysis of DDA-PASEF data and spectral library generation was performed in FragPipe (v. 18.0) according to the default LFQ-MBR workflow. The search parameters were parent and fragment mass error tolerance 15 ppm, protein and peptide false discovery rate (FDR) less than 1%, protease rule—strict trypsin (cleave after KR), and 2 possible missed cleavage sites. Cysteine carbamidomethylation was set as a fixed modification. Methionine oxidation and acetylation of protein N-term were set as variable modifications.

DIA-PASEF data analysis was performed in DIA-NN (v. 1.8.1.) in 2 variants—with an empirically obtained spectral library from FragPipe (DIA) or with a deep learning–predicted spectral library constructed in DIA-NN (DIA-ML) with the same search parameters. The search parameters were default with minor changes: parent and fragment mass error tolerance were set in automatic interference mode and were within 13–15 ppm for all samples, protein and peptide FDR less than 1%, protease rule—strict trypsin (cleave after KR), and 2 possible missed cleavage sites. Cysteine carbamidomethylation was set as a fixed modification. Methionine oxidation and acetylation of protein N-term were set as variable modifications.

### RNA-seq transcriptomics

#### RNA isolation

RNA was isolated using ExtractRNA (Eurogene) using a standard phenol/chloroform extraction procedure according to the manufacturer’s instructions. Briefly, cells were washed with PBS and lysed in a Petri dish by ExtractRNA for 5 minutes at room temperature. Then, lysates were transferred to microcentrifuge tubes and mixed with one-fifth of the volume of chloroform. The samples were incubated for 10 minutes and centrifuged (20 minutes, 12,600 *g*, 4°С). The aqueous phase was mixed with one-half of the initial ExtractRNA volume of ice-cold isopropanol. After incubation for 10 minutes, the samples were centrifuged (20 minutes, 12,600 *g*, 4°С). The precipitate was washed with 70% ethanol, air-dried at room temperature, and dissolved in water. RNA quality was assessed by agarose electrophoresis and Nanodrop spectrometry.

#### Next-generation sequencing

For RNA-seq library preparation, 500 ng of each sample was used with the CORALL Total RNA seq Library Prep Kit (Lexogen), which included poly-A RNA selection according to the manufacturer’s recommendation. The quality of the obtained libraries was verified by capillary electrophoresis using a 4150 TapeStation (Agilent). Finally, the libraries were sequenced on the Illumina NextSeq500 platform using single-end reads. All samples were run simultaneously. Raw reads were deposited in the NCBI SRA database with BioProject identifier PRJNA947173. RNA sequencing was carried out using the equipment of the Core Facility “Medical Genomics” (Tomsk NRMC) and the Tomsk Regional Common Use Center.

#### Data analysis

Quality control of the fastq files was performed using the FASTQC and MultiQC packages. Adapter sequences were removed by the Fastp package. Then, reads were aligned to the *H. sapiens* reference genome GRCh38 using the STAR package, with further quantification by the QoRTs package.

### Statistical analysis

We performed statistical data analysis to compare osteoblasts and VICs at each stage (before and after osteogenic differentiation). Proteomic and transcriptomic data were analyzed separately, and then their results were compared. Statistical analysis was performed using R.

#### RNA-seq data analysis

Regarding the RNA-seq data analysis, we examined 6 samples of osteoblasts (comprising 3 under standard culture conditions and 3 differentiated samples) and 6 samples of VICs (including 3 under standard culture conditions and 3 differentiated samples), each with 2 technical replicates. The RNA-seq count analysis was performed in R using the DESeq2 library per the standard algorithm [17]. The VICs and osteoblasts samples were compared at each time point, encompassing the standard culture conditions (control) and at day 10 of osteogenic differentiation. The technical replicates were consolidated using the collapseReplicates function from the DESeq2 package. During data filtering, genes with counts below 10 were excluded. Statistical analysis involved the Wald test integrated into the standard DESeq2 analysis algorithm. Differentially expressed genes were chosen based on |log2 fold change| > 1 and adjusted *P* < 0.05 criteria. Data normalization entailed applying the rlog transformation. The sample clustering was executed through principal component analysis (PCA), and the conversion of Entrez Gene identifiers was conducted using the bitr function sourced from the clusterProfiler library [18].

Enrichment analysis was performed using the clusterProfiler library, drawing from the Reactome Pathways database [19] and human whole genome annotation org.Hs.eg.db. The differential gene expression results were visualized through various R packages, including pheatmap [20], ComplexHeatmap [21], EnhancedVolcano, and ggplot2 [22]. Additionally, Venn diagrams were generated using the VennDiagram library [23] to illustrate the intersections among differentially expressed genes from VIC and osteoblast comparisons of varying types.

#### Proteomics data analysis

We performed data filtration and imputation of missing values with the NAguideR package for proteomic data analysis [24]. Proteins with missed values in more than half of samples in at least 1 biological group and proteins with a coefficient of variation higher than 0.8 were removed. Then, we selected the optimal method for missed values imputation using “classic criteria,” which was the “Robust data imputation” approach (“Impseqrob”) [25].

Differential expression analysis was performed similarly to RNA-seq data analysis described above, with minor differences. Specifically, we used quantile normalization instead of rlog transformation and limma instead of DESeq2 for proteomics data [26].

#### Qualitative analysis of proteome/transctiptome composition

For the qualitative comparative analysis (presence/absence of transcript/protein) of VICs and osteoblasts, their transcriptomes and proteomes were compared under native (control) and differentiating conditions. To compare the proteomes and transcriptomes, proteins/transcripts present in at least 5 out of 6 samples were selected. For the transcriptome comparison, the dataset also was filtered for genes with fewer than 10 counts. The comparison was performed separately for control and differentiating cells. Enrichment analysis was performed for the unique genes according to the Biological Processes Gene Ontology. Overlap between transcriptomes and proteomes was visualized using Venn diagrams.

## Analyses

### Various proteomics approaches give different proteomics coverage

To obtain maximal proteome coverage and estimate the effectiveness of various types of MS data acquisition on a timsTOF Pro platform for the real biological experiment, we collected 3 datasets for the comparison of osteogenic differentiation of VICs and osteoblasts: (i) classic DDA in PASEF mode of ion mobility trapped mass spectrometry (DDA), (ii) DIA mass spectrometry in PASEF mode with the identification of proteins based on an empirically obtained spectral library in DDA-PASEF mode for prefractionated samples (DIA), or (iii) with the identification based on a library-free search with a spectral library predicted from protein sequences by machine learning in DIA-NN software (DIA-ML).

**Figure 2:**
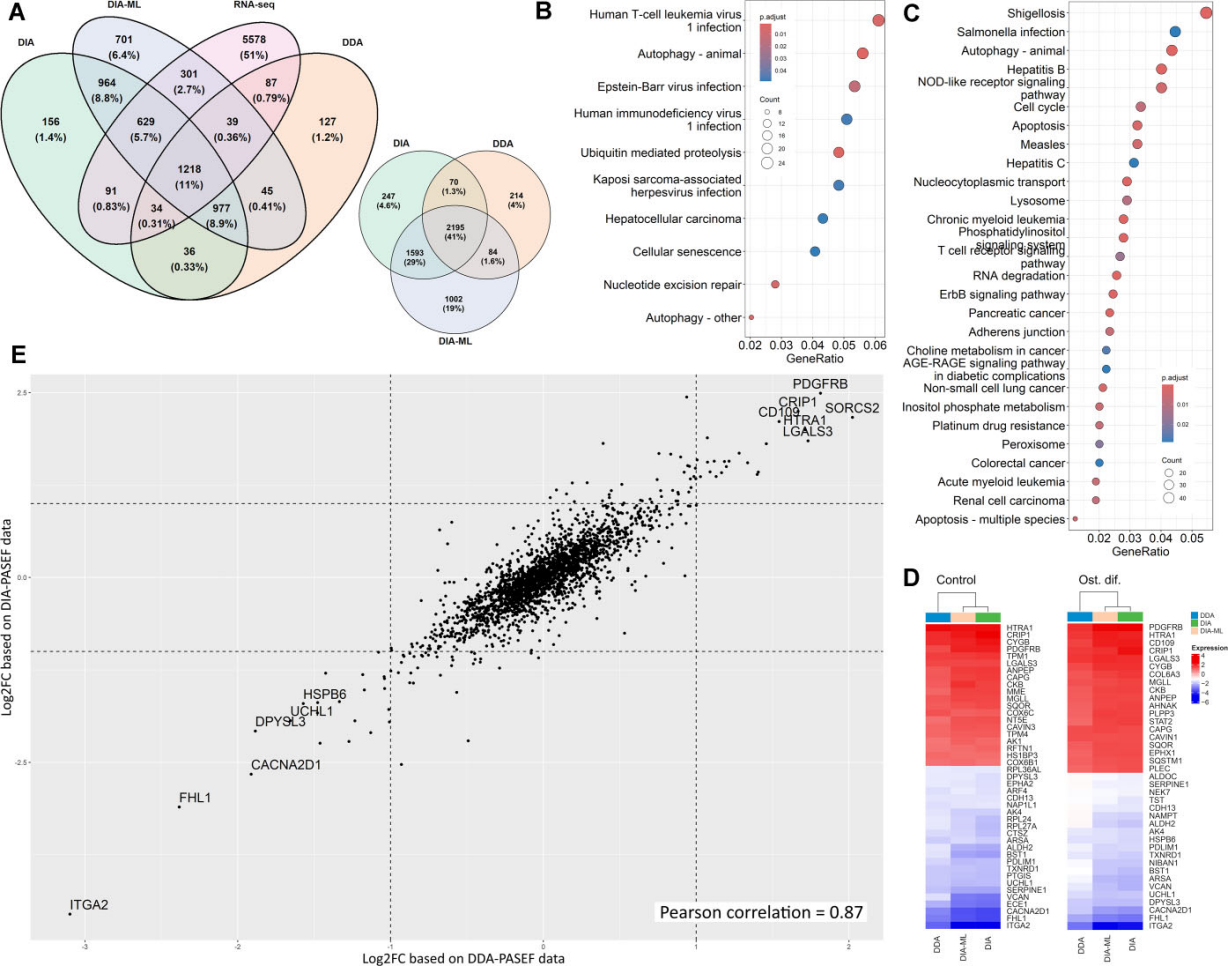
Comparison of 3 proteomics datasets (DDA, DIA, and DIA-ML) and RNA-seq data obtained for shotgun proteomics analysis of mechanisms of osteogenic differentiation of human VICs and osteoblasts. (A) Venn diagrams with overlap between gene products identified by RNA-seq 3 proteomics datasets (left) and between gene products identified in 3 proteomics datasets (right). Proteomics datasets are represented after missed values filtration. (B, C) KEGG pathway enrichment analysis of proteins identified only by DIA-ML (B) or DIA (C). (D) Heatmap demonstrating the log2 fold changes of differentially expressed genes, identified by all 3 proteomics methods between osteoblasts and VICs in standard cultivation (control) and after osteogenic differentiation (Ost. dif.). (E) Correlation of log2 fold changes for differentially expressed proteins compared to differentiated osteoblasts and VICs by DDA and DIA proteomics.

After data filtration, we included 2,563 proteins for DDA, 4,105 proteins for DIA, and 4,874 for the DIA-ML datasets for further analysis (Supplementary Materials 1). So, in our case, classic DIA gives 37.5% higher proteomics coverage than the DDA approach. Summarizing proteins, we found 5,405 proteins identified by at least 1 method and 2,195 identified by all 3 methods. By RNA-seq data, we identified transcripts associated with 7,977 genes. However, we could not expect to have a full overlap of RNA and protein levels within dynamic biological processes as shown in our previous proteotranscriptomic study of VICs osteogenic differentiation [27]. Nevertheless, as soon as RNA-seq is less biased by RNA/protein abundance, we might expect all shotgun proteomics datasets to have similar overlap with RNA-seq data. As expected, we observed a similar number of unique proteins identified exclusively in the DDA and DIA datasets (127 and 156, respectively) while 6.4% (701 protein) of all proteins/transcripts identified were unique for DIA-ML and were not identified in RNA-seq data (Fig. 2A). This might be a sign of the weaker accuracy of this approach, which is expected.

Furthermore, all 3 proteomics datasets identified only 41% of proteins (Fig. 2B), 977 of which were not identified by RNA-seq (Fig. 2a). DIA should be able to identify more proteins, and we see 1,593 proteins identified only in DIA and DIA-ML datasets. Nevertheless, we see 1,002 proteins unique for DIA-ML data besides these DIA-unique proteins.

KEGG pathway enrichment analysis of proteins, unique for DIA-PASEF methods, demonstrates various functional groups, including several signaling pathways: ErbB, NOD-like receptor, T-cell receptor, and phosphatidylinositol signaling system (Fig. 2C). Proteins, unique for DIA-ML data, are mainly associated with RNA and, therefore, with KEGG signaling pathways related to viral infections (Fig. 2B). The DIA-ML dataset includes several proteins important in the focus of our study: TAB2 (MAP3K7 binding protein 2), PIK3CB (phosphatidylinositol-4,5-bisphosphate 3-kinase catalytic subunit beta), PIK3R2 (phosphoinositide-3-kinase regulatory subunit 2), ITGB3 (integrin subunit beta 3), PPP3CB and PPP3CC (protein phosphatase 3 catalytic subunit beta and gamma), CTSK (cathepsin K), FOSL1 (FOS like 1, AP-1 transcription factor subunit), CHUK (a component of inhibitor of nuclear factor kappa B kinase complex), and TRAF2 and TRAF6 (TNF receptor-associated factor 2 and 6)Notably, some of these proteins are also associated with osteoclasts. Still, we did not observe expression of MMP9 and ACP5, identified to be specific markers of the osteoclast lineage by single-cell RNA-seq human bone marrow [28], and as we showed before, we have not found the presence of osteoclast-associated surface antigens (CD34 and CD117) in the osteoblast cells used here [29].

Despite such differences in proteome coverage, these methods seem similar in the quantitation of proteins identified by both methods. We see a good correlation between different methods in pairwise Pearson correlation. The average correlation for the same sample in different datasets is 0.87 (0.83–0.88) between DDA and DIA, 0.87 (0.84–0.88) between DDA and DIA-ML, and 0.98 (0.97–0.98) between DIA and DIA-ML. Accordingly, there is a good correlation between log2 fold changes calculated in different datasets for pairwise comparisons of biological groups studied. The average correlation log2 fold changes in different datasets is 0.83 (0.8–0.87) between DDA and DIA, 0.83 (0.81–0.86) between DDA and DIA-ML, and 0.91 (0.89–0.92) between DIA and DIA-ML. We want to emphasize the apparent correlation between log2 fold changes for differentially expressed proteins and the complete absence of proteins with mutually exclusive expression in different datasets (Fig. 2D-E, Supplementary Materials 2). Still, similar to our previous study of VICs osteogenic differentiation utilizing a multiomics approach [27], we observe weak correlation of RNA-seq and proteomics data (Supplementary Materials 2). Taking into account the fact that our previous study was performed independently with different proteomics and transcriptomics platforms, these phenomena seem to characterize the biological object and not the quality of the data obtained.

### Proteomic and transcriptomic profiles of VICs are distinct from osteoblasts both before and after osteogenic differentiation

In this work, we used osteoblasts and VICs isolated by the standard protocol comprehensively characterized by us previously [29, 30]. Osteoblasts were isolated from the femur of adult patients and represent the terminally differentiated cells expressing RUNX2, BGLAP, and COL1A1, even in standard cultivation, as was shown before. Still, an osteogenic medium is necessary for extracellular matrix mineralization [29]. VICs were isolated from calcified aortic valve leaflets of adult patients, which makes them similar to osteoblasts—both cells were isolated from calcified tissues. Still, VICs do not express osteogenic markers and do not show ALP activity in standard cultivation [30, 31].

**Figure 3:**
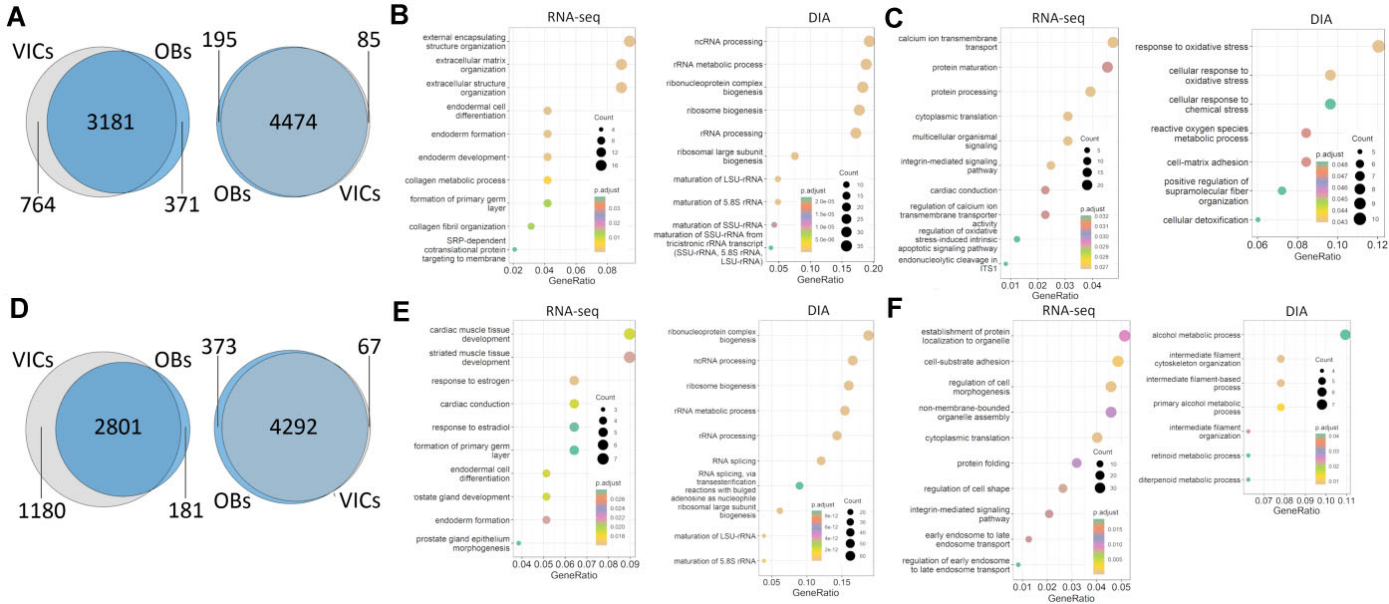
Comparative analysis of transcriptome (RNA-seq) and proteome (DIA) composition in VICs and osteoblasts (OBs) in standard cultivation (A–C) or after osteogenic differentiation (D–F). (A, D) Venn diagrams depicting the overlap of identified transcripts (left) or proteins (right). (B−C, E–F) Enrichment analysis based on “Biological Processes” Gene Ontology for genes (RNA-seq) and proteins (DIA) uniquely expressed in osteoblasts (B, E) or VICs (C, F) under standard cultivation (B, C) or after induction of osteogenic differentiation (E, F).

To better characterize the cells studied, we started from qualitative analysis (presence/absence) of transcriptome and proteome composition (focusing on the DIA dataset as the most representative one). As expected, we observed significant differences in transcriptome composition between VICs and osteoblasts in standard cultivation, while proteome composition was more similar (Fig. 3A; Supplementary Materials 3). Transcripts specific for osteoblasts were associated with extracellular matrix and collagen fibril organization. Proteomic data reinforced the importance of pathways such as ribosome biogenesis and RNA splicing (Fig. 3B). In contrast, for VICs, we observed unique signaling and protein processing transcripts (Fig. 3C). These differences were increased even more after osteogenic differentiation (Fig. 3D-F).

**Figure 4:**
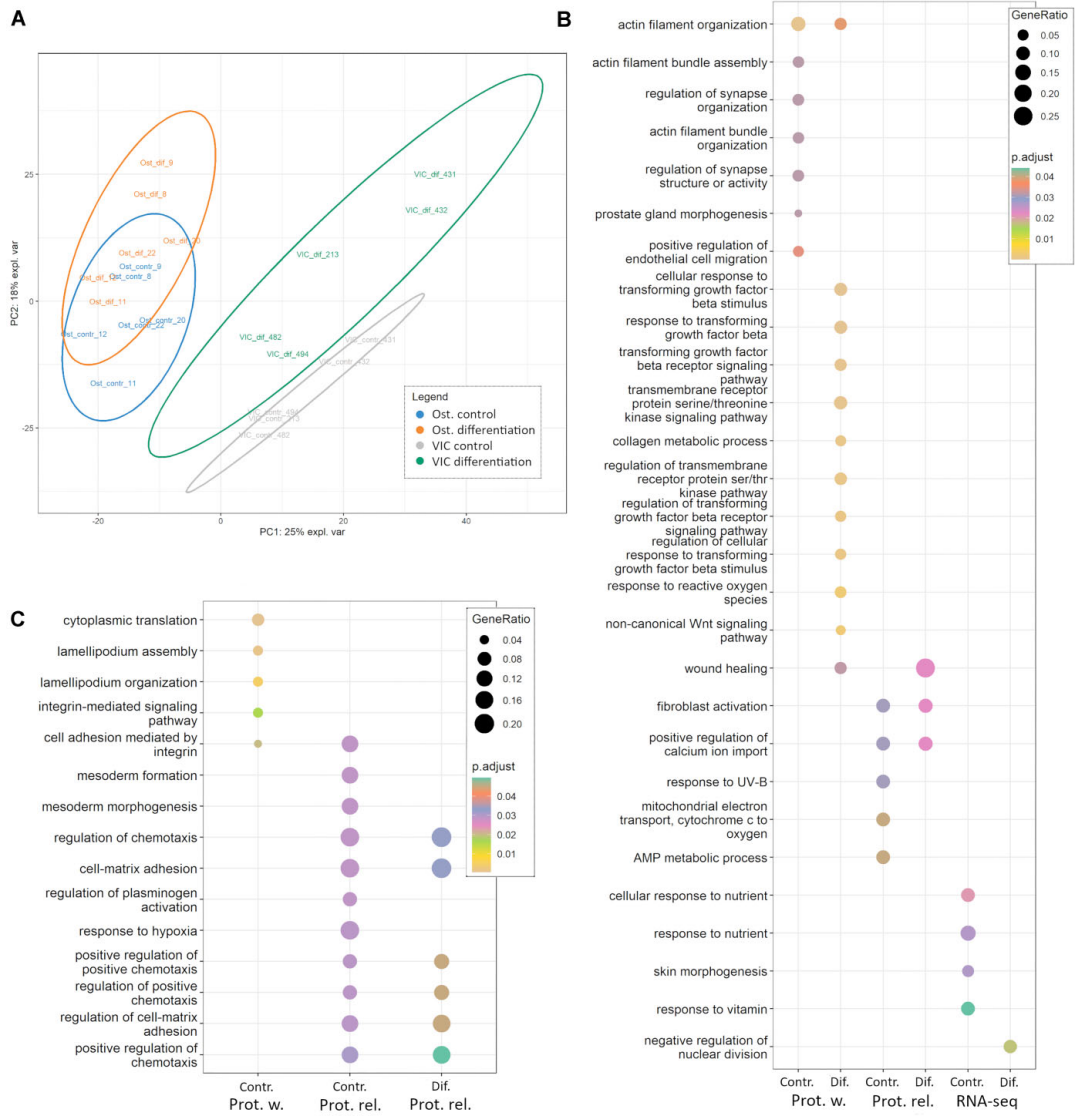
Comparison of human VICs and osteoblasts (Ost) cultured in standard conditions or after induction of osteogenic differentiation. (A) Clusterization of the samples in the PCA dimension based on DIA-PASEF data. Gray—VICs in standard cultivation; green—VICs on day 10 after induction of osteogenic differentiation; blue—osteoblasts in standard cultivation; yellow—osteoblasts on day 10 after induction of osteogenic differentiation. (B) Proteins or transcripts upregulated in osteoblasts during standard cultivation (Contr.) or on day 10 after induction of osteogenic differentiation (Dif.). (C) Proteins or transcripts upregulated in VICs during standard cultivation (contr.) or on day 10 after induction of osteogenic differentiation (Dif.). RNA-seq—transcripts identified by RNA-seq; Prot. w.—differentially expressed proteins, identified by at least 1 shotgun proteomics dataset; Prot. rel.—differentially expressed proteins identified by all 3 shotgun proteomics datasets.

Using the 4 obtained datasets, we then performed quantitative analysis for proteins/transcripts being expressed across all samples. We compared the osteogenic differentiation of VICs (pathological calcification) and osteoblasts (normal ossification). Clustering of the samples by PCA revealed a similar pattern for all datasets (Supplementary Materials 4)—VICs formed distinct clusters before and after osteogenic differentiation and never clustered together with osteoblasts (Fig. 4A). Similar to our previous studies, osteoblasts formed mixed clusters (Fig. 4A) [32, 33].

For optimal data representation, we combined the results of the analysis of a differential expression to the 3 datasets: (i) differentially expressed transcripts identified by RNA-seq, (ii) differentially expressed proteins identified in all 3 proteomics datasets (proteomics reliable, “Prot. rel.”), or (iii) identified in at least 1 of the proteomics datasets (proteomics wide, “Prot. w.”). The datasets include 101, 20, and 162 genes upregulated in osteoblasts and 106, 23, and 208 genes upregulated in VICs, respectively, in standard cultivation. In total, 102, 19, and 206 genes were upregulated in osteoblasts, and 118, 20, and 191 genes were upregulated in VICs, respectively, after the induction of osteogenic differentiation.

In focusing on the Gene Ontology analysis in “Biological Processes,” a limited intersection of differentially expressed genes is noticeable before and after the induction of osteogenic differentiation (Fig. 4). Noteworthy is that the Prot. rel dataset encompasses proteins identified via all 3 proteomic methods (DIA, DDA, and DIA-ML). In osteoblasts, this dataset shows overlaps in the biological processes of fibroblast activation and positive regulation of calcium ion import. In VICs, a more diverse array of processes is observed, primarily associated with chemotaxis, cell adhesion to the extracellular matrix, and regulating these processes.

Under standard culture conditions (control), osteoblasts exhibit the activation of actin filament assembly and organization processes, fibroblast activation, and cellular response to nutrients. In VICs under the same conditions, genes are enriched in lamellipodium assembly and organization, mesoderm formation, cell matrix adhesion, and the positive regulation of chemotaxis. Differentiated osteoblasts show an upregulation of TGF-βnaling and noncanonical Wnt signaling, an increase in collagen metabolism, and the activation of fibroblasts (Fig. 4B). In differentiated VICs, these processes are absent, and the enriched pathways coincide with the pathways identified in undifferentiated VICs (Fig. 4C). It is noteworthy that only about 30% of differentially expressed genes significantly differ between both control and differentiated VICs and osteoblasts. At the same time, about 60% of the genes are differentially expressed between these cell types only before or after osteogenic differentiation (Supplementary Materials 4). Notably, there is a similarity between the Gene Ontology analysis for cell-type specific (Fig. 3) and differentially expressed (Fig. 4) transcripts/proteins. Particularly, both types of analyses identify genes associated with cytoplasmic translation, integrin-mediated signaling pathway, and regulation of cell matrix adhesion for VICs, while for osteoblasts, the similarity is associated with various extracellular matrix–related terms.

We found a low overlap between proteomics and RNA-seq data, which was expected from our previous proteotranscriptomics dataset of VICs osteogenic differentiation (Fig. 4) [27]. Table 2 lists gene products (proteins or transcripts) up- or downregulated between osteoblasts and VICs in both proteomics and RNA-seq data.

**Table 2:** Genes, products of which are up- or downregulated in both RNA-seq and one of the proteomic datasets in comparisons of osteoblasts and VICs in standard cultivation conditions or on day 10 of osteogenic differentiation

	Standard cultivation	Osteogenic differentiation
Upregulated in osteoblasts	**TGFBI, SULF1**, GALNT1, CALD1, POSTN	**TGFBI**, SERPINE2, COL8A1
Upregulated in VICs	**VCAN, EFEMP1, ANK3, CCDC80**, CACNA2D1	**VCAN, EFEMP1, ANK3, CCDC80**, HEG1

Bold marks genes, identified as differentially expressed in both standard cultivation and osteogenic differentiation.

One of the differences between transcriptomic and proteomic datasets is that in the RNA-seq data, we found a surprisingly high proportion of differentially expressed long noncoding RNA (lncRNA). Most of these lncRNAs are known as antisense RNA (Table 3). Notably, many of these antisense RNAs significantly differ only before but not after induction of osteogenic differentiation (Table 3).

**Table 3:** Annotated antisense RNA found in transcriptomes of osteoblasts in comparison with VICs in standard cultivation conditions or on day 10 of osteogenic differentiation. Nonsignificant—adjusted *P* > 0.05.

Upregulated in osteoblasts						
Name	ENSEMBL	Standard cultivation		Osteogenic differentiation		Target gene
		Log2 FC	Adjusted *P* value	Log2 FC	Adjusted *P* value	
HOXA-AS3	ENSG00000254369	8.3	4.82E-09	7.7	2.02E-07	HOXA
HOXA10-AS	ENSG00000253187	8.2	9.84E-09	7.1	1.71E-06	HOXA10
DLX6-AS1	ENSG00000231764	6.0	2.76E-04	7.0	1.86E-05	DLX6
HOXA11-AS	ENSG00000240990	6.8	2.38E-05	5.9	1.93E-03	HOXA11
Lnc-FIGNL2-6	ENSG00000260122	5.8	7.42E-04	5.7	1.06E-03	HSALNG0091106
CHL1-AS1	ENSG00000234661	6.2	3.47E-04	5.6	2.38E-03	CHL1
Lnc-RD3-11	ENSG00000279333	7.6	5.50E-07	5.6	1.32E-03	KCNH1
PENK-AS1	ENSG00000254254	5.2	4.38E-04	5.4	9.61E-08	PENK
HOXD-AS2	ENSG00000237380	Nonsignificant		5.2	2.55E-02	HOXD
Lnc-DAOA-47	ENSG00000284966	4.1	2.01E-10	5.1	7.12E-02	EFNB2
Lnc-SLC25A32-9	ENSG00000253851	6.5	6.51E-07	Nonsignificant		BAALC
Upregulated in valve interstitial cells						
Name	ENSEMBL	Standard cultivation		Osteogenic differentiation		Target gene
		Log2 FC	Adjusted *P* value	Log2 FC	Adjusted *P* value	
Lnc-COPG2-3	ENSG00000270823	1.8	5.13E-07	6.4	2.24E-05	MEST
LAMA5-AS1	ENSG00000228812	Nonsignificant		5.1	8.59E-03	LAMA5
CAVIN2-AS1	ENSG00000233766	3.5	3.94E-02	5.0	1.08E-02	CAVIN2
ANK3-AS	ENSG00000232682	3.6	1.73E-19	4.9	3.14E-37	ANK3
CADM3-AS1	ENSG00000225670	6.6	8.84E-03	Nonsignificant		CADM3
CCDC110-AS	ENSG00000249679	6.07	1.34E-04	4.3	3.23E-02	CCDC110
ADGRD1-AS1	ENSG00000256151	6.0	5.02E-04	Nonsignificant		ADGRD1
Lnc-THNSL1-4	ENSG00000273107	5.9	9.12E-04	Nonsignificant		THNSL1
HAND2-AS1	ENSG00000237125	5.1	5.05E-09	2.5	1.36E-04	HAND2
ENSG00000289061	ENSG00000289061	5.1	1.84E-02	Nonsignificant		EMILIN2

## Discussion

### DIA-PASEF with library-free search is optimal for routine shotgun proteomics

The rapid advancements in shotgun proteomic techniques present a challenge for end users, particularly those who may need to be more expert in proteomics, to determine the most suitable approach for their specific needs. Furthermore, multiple data acquisition modes are available within a single proteomics platform, making it challenging to select the optimal one.

Trapped ion mobility spectrometry (TIMS), utilized in the timsTOF Pro platform, stands out as one of the leading technologies for routine shotgun proteomics. It is particularly valuable for analyses involving limited sample amounts or the need to study large cohorts, as often found in biomedical research. However, comprehensive comparisons of different data acquisition and analysis strategies are scarce from a user’s perspective, particularly those conducted within actual biological objectives and complemented by transcriptomics data.

In this context, we have conducted a comparative study of 2 major proteomic approaches on the timsTOF Pro platform: DDA and DIA with PASEF. We used one of the most modern approaches for data analysis but still available in a user-friendly interface: MSFragger for DDA-PASEF and DIA-NN for DIA-PASEF data [34, 35].

Our biological aim was to compare VICs and osteoblasts osteogenic differentiation *in vitro*. Our samples exhibit greater sophistication than the HeLa samples typically utilized in comparative proteomics research as test samples. This sophistication arises from 3 key factors: (i) The primary function of both VICs and osteoblasts is the secretion of extracellular matrix (ECM). As a result, their proteomes are enriched with numerous ECM proteins, which interfere with identifying less abundant proteins, thereby limiting proteome coverage. (ii) The primary cell cultures in our study are derived from donors with diverse genetic backgrounds and lifestyle factors, introducing a more comprehensive range of biological variability compared to studies using uniform cell lines like HeLa. (iii) The availability of a limited number of biological replicates is a common constraint in most biomedical studies, including ours. This limitation can affect the statistical power and reproducibility of the findings.

As a result, our study quantified half as many protein groups as those reported for HeLa cells [13]. However, DIA-PASEF methodologies proved to be more advantageous in our context. Specifically, DIA-NN library-free search and the conventional library-based DIA analysis increased the quantification of protein groups to 90% and 60%, respectively. A similar dramatic increase in performance was observed in the comparison of DDA- and DIA-PASEF methods for metaproteomics, which possess even more technical challenges than we faced in our study. In the study by Gómez-Varela et al. [12], DIA-PASEF resulted in the quantification of up to 4 times more taxonomic units than DDA-PASEF. This contrasts with the findings of Huang et al. [13], who reported only a 30% increase in quantified proteins for HeLa samples using a similar DDA- and DIA-PASEF comparative analysis.

To evaluate the reliability of our protein identification, we compared the identified proteins with the transcripts detected through RNA-seq transcriptomics. The inherent discrepancies between RNA-seq and proteomics data are well known. It is generally assumed that this phenomenon comes from the variation in translation and protein degradation rates [36, 37]. While the exact reason for the poor correlation of mRNA/protein abundance is hard to define, much phenomenological data have been accumulated. Notably, the study by Gygi et al. [38] represents one of the classic works on yeasts regarding this problem. They showed that the correlation between mRNA and protein abundances cannot predict protein expression. Moreover, the mRNA levels were stable for some genes, but the protein abundances varied more than 20-fold and vice versa. No wonder that in human cells and tissues, we also observe a poor correlation between protein and mRNA abundances, which might be clearly seen in the deep proteome and transcriptome atlas of healthy human tissues [39]. Hundreds of proteins could not be detected even for highly expressed mRNAs. At the same time, protein expression is often more stable across tissues than that of transcripts, which correlates well with our observation regarding higher differences in transcriptome composition between VICs and osteoblasts compared to lower proteome variability (Fig. 3). The mRNA/protein correlation might be especially low for specialized and terminally differentiated tissue; for example, Moritz et al. [40] revealed poor to moderate mRNA/protein correlation varying from 0.34 to 0.54 in the brain. Similarly, our previous study observed a low correlation between RNA-seq and shotgun proteomic data for various mesenchymal cell primary cultures [33] and VICs [27]. Therefore, we expect a limited correlation between transcriptomic and proteomic data here.

Still, comparing the RNA-seq data with various proteomics datasets obtained for the same samples might be used to roughly assess various PASEF techniques. Notably, the highest number of unique proteins was observed with DIA-PASEF data analyzed through a library-free approach, accounting for only 6.4% of all proteins identified (Fig. 2A). Meanwhile, traditional DIA-PASEF using a library-based search yielded the most balanced results in terms of both high protein quantification and methodological overlap. This finding aligns well with existing literature recognizing library-based searches as exceptionally reliable for analyzing DDA and DIA data [41].

Achieving nearly a 2-fold increase in the number of proteins identified is critical for the downstream identification of differentially expressed proteins. This significance is underscored by the fact that highly abundant proteins are often housekeeping proteins, which are remarkably stable from both evolutionary and functional perspectives [42, 43]. In our study, this enhanced identification capability has enabled us to detect hundreds of proteins associated with key cellular compartments (such as peroxisomes and lysosomes) and processes (including endocytosis and apoptosis), as well as numerous signaling pathways, such as ErbB, NOD-like receptor, phosphatidylinositol, T-cell receptor, and EGFR (Fig. 2B, C). As a result, we significantly increased the detection of differentially expressed proteins (DEPs), identifying 371 DEPs in DDA datasets and 646 DEPs in DIA datasets when comparing VICs and osteoblasts after osteogenic differentiation as an example (Supplementary Materials 1).

Further in-depth comparison of DEPs identified by different PASEF protocols revealed a surprisingly high correlation in DEP identification across various methods. The correlation of log2 fold changes (log2FC) between different approaches within all biological comparisons ranged from 0.8 to 0.92. We encountered no DEPs with opposing directions across the datasets (Fig. 2D, E, Supplementary Materials 2). The highest correlation was observed between DIA and DIA-ML approaches (0.91), indicating a robust consistency in our data.

In summary, our observations have shown that the DIA-PASEF method offers a significantly greater advantage over the DDA approach for complex samples in actual proteomics studies, surpassing the performance observed with HeLa test samples. While the library-based search in DIA-PASEF sets a high standard in shotgun proteomics, the DIA-NN–based library-free search presents a more accessible alternative. The library-free search, facilitated by the user-friendly DIA-NN software, rivals the library-based approach in terms of DEPs identified, and it exhibits only a slight reduction in data reliability. Our findings suggest that for routine shotgun proteomics tasks on the timsTOF Pro platform, DIA-PASEF with a library-free search emerges as the preferable method.

### Specificity of VICs osteogenic differentiation offers potential for target CAVD therapy

The comprehensive multiomics dataset discussed in detail above allows us to investigate the differences in molecular mechanisms of normal osteogenic differentiation in osteoblasts and pathological osteogenic differentiation in VICs.

Calcified aortic valves, particularly in the later stages of CAVD, are often presumed to exhibit a high degree of similarity to lamellar bone regarding calcium crystal structure and histological organization. However, upon closer examination, the structures akin to mature bone are observed in only a minority of the CAVD cases [44]. Consequently, our observations reveal significant differences in the mechanisms of osteogenic differentiation between osteoblast-like cells derived from adult bone and VICs isolated from calcified aortic valves in the CAVD latter stages. As previously demonstrated, the osteoblasts proteome does not undergo significant changes during osteogenic differentiation [32, 33]. In contrast, the VICs’ proteome exhibits notable alterations (Supplementary Materials 4) [27]. Before and following differentiation, VICs express higher levels of proteins related to chemotaxis, cell matrix adhesion, and mesodermal markers. Conversely, osteoblasts show an increased expression of proteins involved in osteogenic differentiation, such as those associated with TGF-β and Wnt signaling pathways, collagen synthesis, and calcium ion import. Given the endothelial origins of VICs [7], these differences align well with previous studies that have documented varied osteogenic differentiation mechanisms in cells of different origins [9, 30, 33, 45–47]. Furthermore, research by Nantavisai et al. [45] suggests that these distinctions could be leveraged for the selective inhibition of osteogenic differentiation in specific cell types *in vitro*. From this perspective, proteins exclusively upregulated during VICs osteogenic differentiation present promising targets for selective anti-CAVD treatments (Supplementary Materials 6).

We found an overexpression of monoamine oxidase A (MAOA) during osteogenic differentiation of VICs compared to osteoblasts. Recognized as a critical marker for CAVD, MAOA has been identified as a potential treatment for this disease [48]. Importantly, our data highlight its selectivity in targeting pathological valve calcification, enhancing its therapeutic promise. Additionally, we observed an upregulation of A-kinase anchoring protein 2 (AKAP2) exclusively during VICs osteogenic differentiation. Previous studies have linked AKAP2 mutations with adolescent idiopathic scoliosis and demonstrated the critical role of the AKAP2/ERK1/2 signaling axis in longitudinal bone growth [49, 50]. The specific upregulation of AKAP2 in VICs—unlike in osteoblasts—suggests its association with the early stages of osteogenic differentiation in VICs. Similarly, another protein upregulated in VIC differentiation, GLIPR2, also modulates ERK1/2 signaling, although its role in osteogenic differentiation has not been previously described [51]. The p38 mitogen-activated protein kinase (MAPK) signaling pathway, which includes ERK1/2, is involved in inflammation and is well known to be associated with atherosclerotic and aortic valve calcification [52]. Moreover, it was shown that andrographolide inhibits VICs calcification via the NF-kappa B/Akt/ERK pathway *in vitro*, which supports our data regarding ERK1/2 to be a promising target for selective therapy against aortic valve calcification [53].

### Limitations of the study

Finally, we would like to emphasize the limitations of the study. First, we cultured primary cell cultures for several passages *in vitro* due to the high amount of samples needed. For this, we used a well-defined methodology [9, 27, 29, 30, 33], guaranteeing that we worked with well-defined cell types, but it also limited our observation relative to the native tissue. Still, as discussed above, many of our observations correlate well with the data from the whole-tissue models. Other significant limitations are associated with general poor proteomics reproducibility and rapid development of data analysis tools. Particularly, Varnavides et al. [54] compared 16 shotgun proteomics sample preparation methods and revealed method-dependent differences in specific protein features identification. Beyond sample preparation, the data-dependent acquisition approach used by us for the DDA dataset and spectral library preparation is also known to have limited reproducibility due to stochastic sampling [55]. Finally, the software used in this study was significantly improved even during the time between submitting and publishing the presented manuscript (especially in the case of library-free DIA workflow).

## Supplementary Material

giae110_Supplemental_Files

giae110_GIGA-D-24-00130_Original_Submission

giae110_GIGA-D-24-00130_Revision_1

giae110_Response_to_Reviewer_Comments_Original_Submission

giae110_Reviewer_1_Report_Original_Submission Anton Kutikhin -- 5/31/2024 Reviewed

giae110_Reviewer_2_Report_Original_Submission George R Beck -- 9/23/2024 Reviewed

giae110_Reviewer_2_Report_Revision_1 George R Beck -- 11/4/2024 Reviewed

## Data Availability

The mass spectrometry proteomics data have been deposited to the ProteomeXchange Consortium via the PRIDE [56] partner repository with the dataset identifier PXD036566. Raw reads have been deposited in the NCBI SRA database with BioProject identifier PRJNA947173. All additional supporting data are available in the *GigaScience* repository, GigaDB [57]. Reproducible codes for data analysis in R are available at https://github.com/tniapp/Human-VICs-and-osteoblasts-osteogenic-differentiation.

## Additional Files

**Supplementary Materials 1**. Results of differential expression analysis between VICs and osteoblasts in control and osteogenic differentiation for the 3 proteomics and RNA-seq datasets.

**Supplementary Materials 2**. Correlation of log2 fold changes for differentially expressed proteins between various proteomic datasets and proteomic datasets with RNA-seq data.

**Supplementary Materials 3**. Results of qualitative analysis (presence/absence) of protein/transcripts between VICs and osteoblasts in standard cultivation and after osteogenic differentiation (separate xlsx file).

**Supplementary Materials 4**. Clustering of osteoblasts and human VICs samples by principal component analysis (PCA).

**Supplementary Materials 5**. Venn diagrams representing the overlap of differentially expressed genes and proteins.

**Supplementary Materials 6**. Boxplots for selective proteins.

## Abbreviations

AKAP2: A-kinase anchoring protein 2; CAVD: calcific aortic valve disease; DDA: data-dependent acquisition; DEP: differentially expressed protein; DIA: data-independent acquisition; DMEM: Dulbecco’s modified Eagle’s medium; ECM: extracellular matrix; FBS: fetal bovine serum; HPLC: high-performance liquid chromatography; LC-MS/MS: liquid chromatography/tandem mass spectrometry; lncRNA: long noncoding RNA; MAOA: monoamine oxidase A; MAPK: mitogen-activated protein kinase; PASEF: parallel accumulation serial fragmentation; PBS: phosphate-buffered saline; PCA: principal component analysis; RNA-seq: RNA sequencing; TIMS-TOF: trapped ion mobility spectrometry coupled with time-of-flight mass spectrometry; VIC: valve interstitial cell.
