# Supplementary material for: Similar, but not the same: multiomics comparison of human valve interstitial cells and osteoblast osteogenic differentiation expanded with an estimation of data-dependent and data-independent PASEF proteomics
Source: Gigascience. 2025 Jan 11;14:giae110. doi: 10.1093/gigascience/giae110 (PMC11724719; doi:10.1093/gigascience/giae110)
Supplement: giae110_GIGA-D-24-00130_Revision_1 [file giae110_giga-d-24-00130_revision_1.pdf]

GigaScience

Similar, but not the same: multi-omics comparison of human valve interstitial cells and osteoblast osteogenic differentiation expanded with an estimation of data-dependent and data-independent PASEF proteomics

--Manuscript Draft--

**Manuscript Number:**	GIGA-D-24-00130R1
**Full Title:**	Similar, but not the same: multi-omics comparison of human valve interstitial cells and osteoblast osteogenic differentiation expanded with an estimation of data-dependent and data-independent PASEF proteomics
**Article Type:**	Research
**Funding Information:**	Russian Science Fondation (RU) (23-15-00320) — Prof Anna Malashicheva
**Abstract:**	Osteogenic differentiation is crucial in normal bone formation and pathological calcification, such as calcific aortic valve disease (CAVD). Understanding the proteomic and transcriptomic landscapes underlying this differentiation can unveil potential therapeutic targets for CAVD. In this study, we employed RNA sequencing transcriptomics and proteomics on a timsTOF Pro platform to explore the multi-omics profiles of valve interstitial cells (VICs) and osteoblasts during osteogenic differentiation. For proteomics, we utilized three data acquisition/analysis techniques: Data-Dependent Acquisition (DDA-PASEF) and Data-Independent Acquisition (DIA-PASEF) with a classic library-based (DIA) and machine learning-based library-free search (DIA-ML). Using RNA-seq data as biological reference, we compared these three analytical techniques in the context of actual biological experiments. We use this comprehensive dataset to reveal distinct proteomic and transcriptomic profiles between VICs and osteoblasts, highlighting specific biological processes in their osteogenic differentiation pathways. The study identified potential therapeutic targets specific for VICs osteogenic differentiation in CAVD, including MAOA and ERK1/2 pathway. From a technical perspective, we found that DIA-based methods demonstrate even higher superiority against DDA for more sophisticated human primary cell cultures than it was shown before on HeLa samples. While the classic library-based DIA approach has proved to be a golden standard for shotgun proteomics research, the DIA-ML offers significant advantages with a relatively minor compromise in data reliability, making it the method of choice for routine proteomics.
**Corresponding Author:**	Anna Malashicheva, PhD DrSci<br>Institute of Cytology Russian Academy of Sciences: FGBUN Institut citologii Rossijskoj akademii nauk<br>Sankt-Peterburg, --- Select One --- RUSSIAN FEDERATION
**Corresponding Author Secondary Information:**	
**Corresponding Author's Institution:**	Institute of Cytology Russian Academy of Sciences: FGBUN Institut citologii Rossijskoj akademii nauk
**Corresponding Author's Secondary Institution:**	
**First Author:**	Arseniy Lobov
**First Author Secondary Information:**	
**Order of Authors:**	Arseniy Lobov
	Polina Kuchur
	Nadezhda Boyarskaya
	Daria Perepletchikova
	Ivan Taraskin
	Andrei Ivashkin

Powered by Editorial Manager® and ProduXion Manager® from Aries Systems Corporation

	Daria Kostina
	Irina Khvorova
	Vladimir Uspensky
	Egor Repkin
	Evgeny Denisov
	Tatiana Gerashchenko
	Rashid Tikhilov
	Svetlana Bozhkova
	Vitaly Karelkin
	Chunli Wang
	Kang Xu
	Anna Malashicheva, PhD DrSci
**Order of Authors Secondary Information:**	
**Response to Reviewers:**	We thank the reviewers for their appreciation and thorough evaluation of our study. All the comments have been addressed as detailed below in point-by-point answers. The changes made appear in orange in the manuscript.<br><br>Response to Reviewer #1<br>Summary of the paper. Here, Lobov and colleagues performed a multimodal comparison of osteogenic signatures in valve interstitial cells and osteoblasts. The authors have combined transcriptomic and proteomic techniques and distinct approaches to the data analysis (data-dependent acquisition, data-independent acquisition with classic library-based search, and data-independent acquisition with machine learning-based library-free search). The authors have identified 2,195 proteins common for all data acquisition methods and 5,405 proteins in total (the number significantly exceeding those could be obtained by any of the techniques), and found data-independent acquisition as the most powerful approach (4,105 proteins) for these datasets. Despite aforementioned differences in proteome coverage, these methods showed an excellent correlation when quantifying the protein expression (0.83 - 0.98) that advocates using each of them but still highlights data-independent acquisition as a technique of choice because of the highest proteome coverage. RNA-seq obtained transcripts for around 8,000 genes but transcriptomic and proteomic data did not correlate well. This is a commonly observed phenomenon in multi-omics studies. In vivo, this might be explained by a high proportion of proteins precipitating from systemic circulation rather than being synthesised by cell populations in situ. Here, the authors profiled the RNA and total protein lysate from the cell cultures, suggesting temporal shifts in transcription and translation of the same molecules.<br>The combination of distinct techniques and data analysis modalities is a significant advantage of the paper as it improves the methodological basis for analysing multi-omics data and raises the question on optimising the workflow of bioinformatics analysis in relation to the proteomic data.<br>The authors showed clear clusterisation of undifferentiated (i.e., primary) and differentiated valvular interstitial cells and identified a set of osteogenic proteins charasteristic of VIC-derived osteoblasts. Notably, the molecular pattern of osteogenic differentiation in valvular interstitial cells demonstrated slight differences in comparison with osteoblasts.<br>General comment<br>Overall, the study has no glaring omission and deserves to be published, although I would like to recommend extending Discussion by commenting the following issues:...<br>Authors' response: We sincerely thank the reviewer for appreciating the value of this work and extending the Discussion according to the comments below.<br>Comment 1.<br>The reasons behind such differences between transcriptomic and proteomic data?<br>Authors' response:<br>Thank you for the suggestion to discuss this point in more detail. We agreed that it is indeed an important and interesting question. We added a short paragraph to discuss

Powered by Editorial Manager® and ProduXion Manager® from Aries Systems Corporation

this:
“The inherent discrepancies between RNA-seq and proteomics data are well known. It is generally assumed that this phenomenon comes from the variation in translation and protein degradation rates.[35,36] While the exact reason for the poor correlation of mRNA/protein abundance is hard to define, much phenomenological data has been accumulated. Notably, Gygi et al. (1999) represent one of the classic works on yeasts regarding this problem. They showed that the correlation between mRNA and protein abundances cannot predict protein expression. Moreover, the mRNA levels were stable for some genes, but the protein abundances varied more than 20-fold and vice versa.[37] No wonder that in human cells and tissues, we also observe a poor correlation between protein and mRNA abundances, which might be clearly seen in the deep proteome and transcriptome atlas of healthy human tissues.[38] Hundreds of proteins could not be detected even for highly expressed mRNAs. At the same time, protein expression is often more stable across tissues than that of transcripts, which correlates well with our observation regarding higher differences in transcriptome composition between VICs and osteoblasts compared to lower proteome variability (Fig. 3). The mRNA/protein correlation might be especially low for specialized and terminally differentiated tissue, e.g. Moritz et al. (2019) revealed poor to moderate mRNA/protein correlation varying from 0.34 to 0.54 in brain.[39] Similarly, our previous study observed a low correlation between RNA-seq and shotgun proteomic data for various mesenchymal cell primary cultures [32] and VICs [27]. Therefore, we expect a limited correlation between transcriptomic and proteomic data here. Still, comparing the RNA-seq data with various proteomics datasets obtained for the same samples might be used to roughly assess various PASEF techniques.“

Comment 2.
The "core sets" of proteins characterising the osteogenic differentiation which are: 1) unique or top differentially expressed in bone-derived and VIC-derived osteoblasts; 2) have been either identified by all three proteomic data acquisition approaches or show similar results when analysing transcriptomic and proteomic data. Adding a table might be useful to show a focused summary for the subsequent studies.
Authors’ response:
Thank you for raising this question. As suggested, we added the qualitative analysis showing proteins/transcripts unique for osteoblasts and VICs after osteogenic differentiation. This analysis is represented in figure 3 and supplementary materials 3 in the current version of the manuscript:
“To better characterize the cells studied we started from qualitative analysis (presence/absence) of transcriptome and proteome composition (focusing on DIA dataset as the most representative one). As expected, we observed significant differences in transcriptome composition between VICs and osteoblasts in standard cultivation, while proteome composition was more similar (Fig. 3 a; supplementary materials 3). Transcripts specific for osteoblasts are associated with extracellular matrix and collagen fibril organization. Proteomic data reinforce the importance of pathways such as ribosome biogenesis and RNA splicing (Fig. 3 b). In contrast, for VICs we observed unique signaling and protein processing transcripts (Fig. 3 c). These differences become increased even more after osteogenic differentiation (Fig. 3 d-f).”
Regarding the second suggestion, we would like to emphesize that we underlined proteins being overexpressed by both RNA-seq and proteomics data in Table 2 and focused the discussion on them. We agreed that these proteins are the most interesting ones.

Comment 3.
The correlations between the differentially expressed pathways and abovementioned osteogenic protein signatures of bone-derived and VIC-derived osteoblasts (which proteins do correspond to which pathway). Adding a table might be useful to show a focused summary for the subsequent studies.
Authors’ response:
As suggested, we added the pathway enrichment analysis for the unique proteins mentioned above in Figure 3 (please see also the detailed answer within the answer to comment 2). We also briefly discussed comparing this analysis's results with the pathway enrichment results for differentially expressed proteins:
“Notably, there is a similarity between the gene ontology analysis for cell-type specific (Fig. 3) and differentially expressed (Fig. 4) transcripts/proteins. Particularly, both types of analyses identify genes associated with cytoplasmic translation, integrin-mediated

signaling pathway, and regulation of cell matrix adhesion for VICs; while for osteoblasts, the similarity is associated with various extracellular matrix-related terms."

Response to Reviewer #2

Summary of the paper. This study employed RNA sequencing transcriptomics and proteomics to explore the multi-omics profiles of valve interstitial cells (VICs) and osteoblasts during osteogenic differentiation. The study identified potential therapeutic targets specific for VICs osteogenic differentiation in CAVD, including MAOA and ERK1/2 pathway. The authors conclude that DIA-based methods demonstrate even higher superiority against DDA for more sophisticated human primary cell cultures than it was shown before on test samples. While the classic library-based DIA approach has proved to be a golden standard for shotgun proteomics research, the DIA-ML offers significant advantages with a relatively minor compromise in data reliability, making it the method of choice for routine proteomics.

General comment

The study addresses and interesting and biologically relevant topic. The comparison of proteomic techniques should be of interest to readers. However, there are questions about the nature of cells being analyzed with the seeming lack of osteoblast specific genes/proteins suggesting either mixed cell lineages or possibly a more terminally differentiated cell population such as osteocytes, How this would affect the interpretation of the data is not clear. A cleaner characterization of the cell populations would strengthen the manuscript.

Authors' response: We sincerely thank the reviewer for the feedback and for recognizing the relevance of our work. We agree that a more detailed characterization of the cell populations might enhance the manuscript. However, the primary focus of our study is on the comparative analysis of proteomic techniques rather than on cellular characterization. The cells we used were comprehensively characterized in several of our recent studies (Kostina et al., 2021, 2022; Semenova et al., 2022; Bogdanova et al., 2019), where their osteoblast-specific markers and differentiation status were well-defined.

To maintain focus on the technical aspects of shotgun proteomics, we opted to limit the scope of the cellular characterization in this paper. Nevertheless, we have carefully addressed the concerns raised by citing our previous papers, where we introduced these cell isolation protocols, and by thoroughly discussing the potential implications of interpreting our data. We believe this approach balances the technical emphasis of our current work and the biological context. Please find more detailed answers below.

Comment 1-2.

The isolation protocol for bone is likely collecting more osteocytes than osteoblasts. Data figure of OB characterization. Alizarin Red S staining was performed but data not clearly shown. Is this the 2 wells in Fig.1? If so, the manuscript would be significantly improved by including a better characterization of the cells. As ARS binds calcium it is possible that the positive stating is a Calcium-phosphate precipitate and not necessarily hydroxyapatite which is normally formed initially as nodules. Therefore, increased ALP staining or enzyme activity, changes in maker gene/protein expression such as Col-1, BSP, osteocalcin, OSX, Runx2, etc. would make a stronger case that the cells are actually differentiating to osteoblasts as claimed. Likewise, some characterization of the VICs would also strengthen the conclusions.

Authors' response:

We limited the characterization of the cells in this paper because we comprehensively characterized these cells and their osteogenic differentiation in detail in previous papers (Kostina et al., 2021, 2022; Semenova et al., 2022; Bogdanova et al., 2019), and here, we aimed to focus on the proteomics data analysis rather than on the biology of these cell types. Nevertheless, we agreed that this is an important concern. Therefore, we included additional explanations in the analyses section:

"In this work, we used osteoblast and valve interstitial cells (VICs) isolated by the standard protocol comprehensively characterized by us previously [28,29]. Osteoblasts were isolated from a femur of adult patients and represent the terminally differentiated cells expressing RUNX2, BGLAP, and COL1A1 even in standard cultivation as was shown before. Still, an osteogenic medium is necessary for extracellular matrix mineralization [28]. VICs were isolated from calcified aortic valve leaflets of adult patients, which makes them similar to osteoblasts - both cells were isolated from calcified tissues. Still, VICs do not express osteogenic markers and do not show ALP activity in standard cultivation [29,30]."

Comment 3.

Powered by Editorial Manager® and ProduXion Manager® from Aries Systems Corporation

A Table of OB or VIC specific gene/protein expression, even if not significantly altered might provide additional information as to the characterization of the cells.
Authors' response:
Thank you for this suggestion. We included the qualitative analysis to characterize the gene/protein expression profiles of osteoblasts and VICs. The analysis is presented now as figure 3 with the description as follow:
"To better characterize the cells studied we started from qualitative analysis (presence/absence) of transcriptome and proteome (focusing on DIA dataset as the most representative one) composition. As expected, we observed significant differences in transcriptome composition between VICs and osteoblasts in standard cultivation, while proteome composition was more similar (Fig. 3 a). Transcripts specific for osteoblasts are associated with extracellular matrix and collagen fibril organization. Proteomic data reinforce the importance of pathways such as ribosome biogenesis and RNA splicing (Fig. 3 b). In contrast, for VICs we observed unique signaling and protein processing transcripts (Fig. 3 c). These differences become increase even more after osteogenic differentiation (Fig. 3 d-f)."

Comment 4.
Osteoclast genes are noted with the osteoblast differentiation dataset however these are very different cell types expressing a very different gene profile so the result might suggest contamination with osteoclasts and mixed lineages of cell populations?
Authors' response:
This is a very important observation; indeed, the osteoblasts express some of the proteins traditionally regarded as specific for osteoclasts. The recent development in the single-cell RNA-seq data allows us to uncover natural cell heterogeneity. It shows that despite the significant differences between various cell types (particularly between osteoblasts and osteoclasts), the number of qualitative differences (presence/absence of protein expression) is limited (Lee et al., 2023). Specifically, by the data of human bone marrow scRNA-seq atlas, many osteoclast-related proteins can be found in osteoblasts (https://www.immunesinglecell.org/atlas/bone_marrow; Lee et al., 2023). MMP9, ACP5 and CTSK expression are regarded as specific markers of osteoclasts. Indeed, the MMP9 and ACP5 are not expressed in our samples, which clearly shows that we do not have osteoclast contamination.
Moreover, osteoclasts are myeloid-origin cells expressing specific surface antigens (CD34 and CD117). Previously, we described in detail the protocol for osteoblasts isolation used here and showed the absence of these markers by flow cytometry (Kostina et al., 2022). So, the surface antigen markers for osteoblasts used in this work are different for osteoclasts.
Finally, we emphasize that the expression of some osteoclast-related genes in osteoblasts is well-known in the literature. Moreover, the expression of many of them in the osteoblasts is vital for osteogenesis. In particular, we would like to underline TAB2, PIK3CB, PIK3R2, and CTSK, which were found to be expressed in osteoblasts in our study. Kim et al. (2018), showed the deregulation of NF-κB by TAB2 degradation in osteoblasts and osteoclasts types (Kim et al., 2018). Similarly, PI-3-kinase class I plays a central role in bone formation through SMAD1 regulation in osteoblasts (Gámez et al., 2016). CTSK is traditionally regarded as an osteoclast protease, but osteoblasts are known to secrete it and also contribute to extracellular matrix remodeling (Mandelin et al., 2006).
We also added clarification regarding this in the Analyses section:
"Still, we do not observed expression of MMP9 and ACP5, identified to be specific markers of osteoclast linage by scRNA-seq human bone marrow atlas (immunesinglecell.org/atlas/bone_marrow; accessed 11/10/24)[28] and, as we showed before, we have not found the presence of osteoclast-associated surface antigens (CD34 and CD117) in the osteoblast cells used here.[29]"
Comment 5.
The fact that the cells were cultured instead of taken straight from the patient might also confound interpretation as this likely significantly alters gene and protein expression.
Authors' response:
We absolutely agree that the best way for our study would be to take the cells directly from the patient. Nevertheless, it is technically impossible due to the limitations for sample amounts in shotgun proteomics. This is especially true for our study in which we used the same cells for RNA-seq, protein fractionation for spectral library preparation, and two runs of shotgun proteomics with various data acquisition modes.

Powered by Editorial Manager® and ProduXion Manager® from Aries Systems Corporation

	Even with the high sensitivity of the TimsToF platform, the amount of protein that might be isolated straight from the patient would not be enough.<br>To make these limitations also clear for the readers, we added the “Limitations of the study” section at the end of the discussion:<br>“Finally, we would like to emphasize the limitations of the study. First, we cultured primary cell cultures for several passages in vitro due to the high amount of samples needed. For this, we used a well-defined methodology [9, 27,29,30,33], guaranteeing that we worked with well-defined cell types but also limited our observation relative to the native tissue. Still, as discussed above, many of our observations correlate well with the data from the whole-tissue models. Other significant limitations are associated with general poor proteomics reproducibility and rapid development of data analysis tools. Particularly, Varnavides et al. (2022) compared 16 shotgun proteomics sample preparation methods and revealed method-dependent differences in specific protein features identification.[54] Beyond sample preparation, the data-dependent acquisition approach used by us for the DDA dataset and spectral library preparation is also known to have limited reproducibility due to stochastic sampling.[55] Finally, the software used in this study was significantly improved even during the time between submitting and publishing the presented manuscript (especially in the case of library-free DIA workflow).”
**Additional Information:**	
**Question**	**Response**
Are you submitting this manuscript to a special series or article collection?	No
**Experimental design and statistics**<br><br>Full details of the experimental design and statistical methods used should be given in the Methods section, as detailed in our Minimum Standards Reporting Checklist. Information essential to interpreting the data presented should be made available in the figure legends.<br><br>Have you included all the information requested in your manuscript?	Yes
**Resources**<br><br>A description of all resources used, including antibodies, cell lines, animals and software tools, with enough information to allow them to be uniquely identified, should be included in the Methods section. Authors are strongly encouraged to cite Research Resource Identifiers (RRIDs) for antibodies, model organisms and tools, where possible.<br><br>Have you included the information requested as detailed in our Minimum	Yes

Powered by Editorial Manager® and ProduXion Manager® from Aries Systems Corporation

Standards Reporting Checklist?	
**Availability of data and materials**<br><br>All datasets and code on which the conclusions of the paper rely must be either included in your submission or deposited in publicly available repositories (where available and ethically appropriate), referencing such data using a unique identifier in the references and in the “Availability of Data and Materials” section of your manuscript.<br><br>Have you have met the above requirement as detailed in our Minimum Standards Reporting Checklist?	Yes

Powered by Editorial Manager® and ProduXion Manager® from Aries Systems Corporation

Manuscript

Click here to access/download;Manuscript;Lobov_et_al_GigaScience_R1-

Similar, but not the same: multi-omics comparison of human valve interstitial cells and osteoblast osteogenic differentiation expanded with an estimation of data-dependent and data-independent PASEF proteomics

Arseniy Lobov[1*#]& Polina Kuchur[1#], Nadezhda Boyarskaya[2], Daria Perepletchikova[1], Ivan Taraskin[1], Andrei Ivashkin[1], Daria Kostina[1], Irina Khvorova[1], Vladimir Uspensky[2], Egor Repkin[3], Evgeny Denisov[4], Tatiana Gerashchenko[4], Rashid Tikhilov[5], Svetlana Bozhkova[5], Vitaly Karelkin[5], Chunli Wang[6], Kang Xu[6], Anna Malashicheva[1*]

[1]Institute of Cytology Russian Academy of Science, St. Petersburg, Russia
[2]Almazov National Medical Research Centre, St. Petersburg, Russia
[3]Centre for Molecular and Cell Technologies, St. Petersburg State University, St. Petersburg, Russia
[4]Laboratory of Cancer Progression Biology, Cancer Research Institute, Tomsk National Research Medical Center, Russian Academy of Sciences, Tomsk, Russia
[5]Vreden National Medical Research Center of Traumatology and Orthopedics, St. Petersburg, Russia
[6]School of Pharmacy, Hubei University of Chinese Medicine, Wuhan, China

* Corresponding authors: malashicheva@incras.ru arseniylobov@gmail.com
[#] These authors contributed equally to this work
ORCID iDs: Arseniy Lobov [0000-0002-0930-1171]; Polina Kuchur [0000-0002-9415-577X]; Nadezhda Boyarskaya [0000-0002-6402-770X]; Daria Perepletchikova [0000-0002-5056-3368]; Ivan Taraskin [0009-0001-7251-1067]; Andrei Ivashkin [0009-0006-6039-3960]; Daria Kostina [0000-0003-0641-8101]; Irina Khvorova [0000-0002-7124-9749]; Vladimir Uspensky [0000-0002-7929-0594]; Egor Repkin [0000-0002-8599-3173]; Evgeny Denisov [0000-0003-2923-9755]; Tatiana Gerashchenko [0000-0002-7283-0092]; Rashid Tikhilov [0000-0003-0733-2414]; Svetlana Bozhkova [0000-0002-2083-2424]; Vitaly Karelkin [0009-0005-3020-2417]; Chunli Wang [0000-0001-8891-1797]; Kang Xu [0000-0002-8492-2058]; Anna Malashicheva [0000-0002-0820-2913]

Abstract

Osteogenic differentiation is crucial in normal bone formation and pathological calcification, such as calcific aortic valve disease (CAVD). Understanding the proteomic and transcriptomic landscapes underlying this differentiation can unveil potential therapeutic targets for CAVD. In this study, we employed RNA sequencing transcriptomics and proteomics on a timsTOF Pro platform to explore the multi-omics profiles of valve interstitial cells (VICs) and osteoblasts during osteogenic differentiation. For proteomics, we utilized three data acquisition/analysis techniques: Data-Dependent Acquisition (DDA-PASEF) and Data-Independent Acquisition (DIA-PASEF) with a classic library-based (DIA) and machine learning-based library-free search (DIA-ML). Using RNA-seq data as biological reference, we compared these three analytical techniques in the context of actual biological experiments. We use this comprehensive dataset to reveal distinct proteomic and transcriptomic profiles between VICs and osteoblasts, highlighting specific biological processes in their osteogenic differentiation pathways. The study identified potential therapeutic targets specific for VICs osteogenic differentiation in CAVD, including MAOA and ERK1/2 pathway. From a technical perspective, we found that DIA-based methods demonstrate even higher superiority against DDA for more sophisticated human primary cell cultures than it was shown before on HeLa samples. While the classic library-based DIA approach has proved to be a golden standard for shotgun proteomics research, the DIA-ML offers significant advantages

with a relatively minor compromise in data reliability, making it the method of choice for routine proteomics.

Keywords: timsTOF Pro, Data-Independent Acquisition, DIA-PASEF, calcific aortic valve disease (CAVD), valve interstitial cells, osteogenic differentiation

Background

Vessels and bone tissues exhibit intricate interconnections, where vessels provide a structural framework for bone formation and play a pivotal role in governing bone development and repair.[1] This interrelation clarifies why, in pathological conditions, vascular tissues may undergo calcification. Calcifications can manifest in diverse forms and mechanisms throughout the body, significantly elevating health risks irrespective of location. Notably, meta-analyses have indicated that calcification in any arterial wall is linked to a 3–4-fold increase in mortality risk and cardiovascular events.[2]

Calcific aortic valve disease (CAVD) or calcific aortic valve stenosis is one of the most dangerous forms of vascular calcification. CAVD is a gradual condition that initiates with aortic valve thickening and gradually advances to extensive calcification. Subsequently, valve calcification in advanced stages can precipitate potentially life-threatening circulatory disorders. The prevalence of CAVD has exhibited substantial growth over time. Recent statistics indicate a rise in incident cases of CAVD globally, from 130,822 in 1990 to 589,638 in 2019.[3] During this period, CAVD witnessed the highest surge in death rates and prevalence among the three primary valvular diseases, including rheumatic heart disease, mitral regurgitation, and CAVD.[4] Over the past three decades, many Western nations have witnessed a prevalence increase of over 10% in CAVD.[4] While comprehensive data is lacking for numerous resource-poor countries, the global trend underscores a universal concern.[4,5] The existing treatment for CAVD entails surgical valve replacement using either a bio- or mechanical prosthesis, necessitating replacement every 5-15 years. It is imperative to develop therapeutic strategies that can mitigate the progression of CAVD urgently.

A fundamental lack of understanding regarding the pathogenesis of CAVD stands as a critical barrier to therapeutic interventions. While the endothelium is believed to be significant in initiating CAVD[6], the osteogenic transformation of resident valve interstitial cells (VICs) is central to disease progression and valve calcification.[5,7]

While there are many high-quality studies on the molecular mechanisms behind VICs osteogenic differentiation[8], there are still gaps in our understanding. To develop potential therapies, it is essential to understand the differences between VICs osteogenic trans-differentiation and normal osteogenic differentiation. Although it is generally assumed that osteogenic differentiation of VICs is similar to that of osteoblasts, there is still no empirical confirmation of this assumption because of the lack of multi-omics studies that describe the molecular mechanisms of adult osteoblast differentiation and physiology.[9] Therefore, we

aimed to perform the multi-omics comparison of molecular mechanisms of VICs and osteoblast osteogenic differentiation *in vitro*.

In omics, particularly in proteomics research, the depth of biological insights is constrained by the technical capabilities of omics methodologies. The field of mass spectrometry-based omics is advancing swiftly, with novel platforms for proteomics emerging regularly. This progress necessitates the development of specialized bioinformatics methods. However, given that the primary users of mass spectrometry-based omics frequently include biologists and clinicians who may not have expertise in mass spectrometry, conducting comparative studies is crucial. Such studies should elucidate how different platforms and data acquisition strategies impact the ability to address biological questions effectively.

The Trapped Ion Mobility Spectrometry coupled with Time-of-Flight Mass Spectrometry (TIMS-TOF) technology, exemplified by the timsTOF Pro platform from Bruker, represents a cutting-edge advancement in shotgun proteomics, showcasing superior performance capabilities.[10] This platform supports both Data-Dependent Acquisition (DDA) and Data-Independent Acquisition (DIA) strategies, offering flexibility in experimental approaches. Moreover, various bioinformatics tools are accessible for analyzing data derived from either approach. Despite numerous studies comparing these methodologies and their corresponding analysis software using standard test samples, such as HeLa cell lysate, a significant gap exists in the literature concerning their comparison in the context of addressing specific biological inquiries in biomedical studies.[11–14]

To bridge biological and technical gaps in current knowledge, we conducted a comparative study on the molecular mechanisms underlying osteogenic differentiation in human osteoblasts isolated from adult femur bones and VICs from patients with CAVD. Our methodology combined transcriptomics with shotgun proteomics. To achieve the most comprehensive proteomic coverage and assess various PASEF techniques, we analyzed the same samples using DDA- and DIA-PASEF. For the DIA-PASEF data, a library-free search strategy utilizing DIA-NN was employed alongside a traditional library-based approach, constructing a spectral library from pre-fractionated pooled samples of each biological group under investigation.

Our findings reveal differences in molecular mechanisms of osteoblasts and VICs osteogenic differentiation, which might be used to target anti-CAVD therapy development. Specifically, we underline MAOA and ERK1/2 as fruitful targets, which are upregulated in VICs osteogenic differentiation and have been shown to have the potential for CAVD treatment before. From a technical perspective, we show that for complex samples, such as human primary cell cultures, DIA-PASEF outperforms DDA to an even greater extent than previously demonstrated with test samples. While classic DIA-PASEF with library-based data analysis appears to be a golden standard, the library-free search approach with DIA-NN proves nearly as efficient as the library-based method in recognizing differentially expressed proteins. By comparing the RNA-seq data, we observed only a minor compromise in data reliability with the DIA-NN method compared to library-based analysis.

Methods

A schematic representation of the study design is provided in Figure 1, followed by a detailed explanation in the text below.

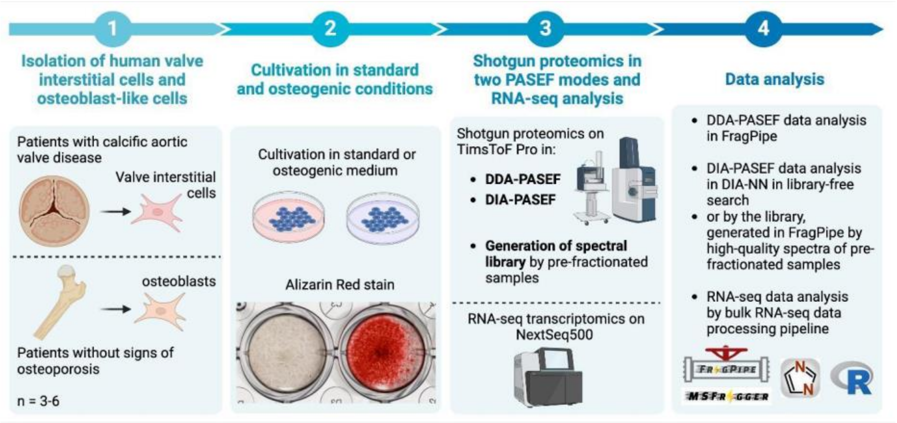

Figure 1 A schematic representation of the study design.

2.1. Cell cultures

Valve interstitial cell isolation

Human valve interstitial cells (VICs) were obtained from the calcified human aortic valves (n = 5), which were isolated from patients with calcific aortic valve disease (CAVD) during surgical valve replacement in the Almazov National Medical Research Centre of the Ministry of Health of the Russian Federation. The study was conducted according to the guidelines of the Declaration of Helsinki and approved by the local Ethics Committee of the Almazov Federal Medical Research Center. All patients gave informed consent. Patients with bicuspid valves were excluded.

Fragments of calcified valve were washed in PBS and treated with collagenase II (1 mg/ml; Worthington Biochemical Corporation) in PBS for 10 minutes, and then endothelial cells were scraped out. The tissues were then digested by collagenase II in a standard cultivation medium overnight at 37 °C. Tissue fragments were transferred to a cultural flask.

Osteoblasts isolation

Human osteoblast-like cells were obtained from the fragments of the epiphysis of bone spongy tissue of femur bone (n = 6), harvested during surgery at Vreden National Medical Research Center of Traumatology and Orthopedics. The local Ethics Committee of Vreden National Medical Research Center of Traumatology and Orthopedics approved the clinical research protocol and followed the Declaration of Helsinki principle. All patients gave informed consent. Patients with osteoporosis were excluded.

Bone fragments were washed in phosphate buffer saline (PBS) supplemented with Penicillin/Streptomycin 3 times. Then, the fragments were crushed into fragments up to 0.5 mm using carbide cutters. Cancellous bone was repeatedly washed in PBS until connective tissue and capillary were removed. Then, the fragments were incubated with 0.2% collagenase type II (Worthington Biochemical Corporation) solution in PBS for 30 min at 37°C, washed in PBS, and transferred into 0.2% collagenase type IV solution (Worthington Biochemical

Corporation) in standard cultivation media and incubated for 16 hours at 37°C. Then, fragments were transferred to a culture flask, where cells were explanted for several weeks.

Cell culturing

Both VICs and osteoblasts were cultured in the same conditions (37 °C in 5% CO_2) in DMEM (41966-052, Gibco) supplemented with 15% FBS (HyClone, GE Healthcare), 2 mM L-glutamine (Gibco) and penicillin/streptomycin (Gibco) until 70–80% confluence. The medium was changed twice a week. Cell cultures were routinely checked for mycoplasma contamination each week, according to Janetzko et al. 2014[15] with minor modifications. Cells of 3-4 passages were used for the experiments.

Osteogenic differentiation

We used cells from the same donors for both transcriptomics and proteomics analysis. For both methods, cells were analyzed in standard cultivation and on the 10th day after induction of osteogenic differentiation.

To induce osteogenic differentiation, we used a classic osteogenic medium, usually used for *in vitro* osteogenic differentiation: DMEM supplemented with 10% FBS, 2 mM L-glutamine, penicillin/streptomycin, 50 mg/ml ascorbic acid, 0.1 mM dexamethasone, and 10 mM b–glycerophosphate.[9]

Osteogenic differentiation was verified by Alizarin red stain (stain for calcium deposition) on the 21st day of osteogenic differentiation. For this purpose, in parallel with the main experiment, cells were plated in 24-well plates. After 21 days of differentiation, cells were washed with PBS, fixed in 70% ethanol for 60 min, washed twice with distilled water, and stained using Alizarin Red solution (Sigma).

2.2. Shotgun proteomics

Protein isolation

The cells were washed twice with PBS and lysed in a Petri dish with RIPA buffer (ThermoFisher Scientific) supplemented with a complete protease inhibitor cocktail (Roche). Then, the dishes were transferred to the ice for 30 min. Cell lysates were stored at −80°C before use.

The samples were sonicated for 10 min in an ultrasonic bath with ice and centrifuged (12 000 g, 20 min, 4°C). Proteins were cleaned by standard acetone precipitation (EM grade acetone; EMS). The protein pellet was resuspended in 8M Urea/50mM ammonium bicarbonate (Sigma Aldrich) and centrifuged (12 000 g, 10 min, 4°C). The protein concentration was measured by a Qubit fluorometer (ThermoFisher Scientific) using QuDye Protein Quantification Kit (Lumiprobe) according to the manufacturer's recommendations.

In-solution digestion

20 µg of each sample was digested by trypsin. Sample volumes were equalized by the addition of 8M Urea/50mM ammonium bicarbonate. Disulfide bonds were reduced and alkylated by incubation for 1 h at 37 °C in 5 mM DTT (Sigma Aldrich) with subsequent incubation in 15 mM iodoacetamide for 30 min in the dark at room temperature (Sigma

Aldrich). Then, the samples were diluted with seven volumes of 50 mM ammonium bicarbonate and incubated for 16 h at 37 °C with 400 ng of trypsin (ratio 1:50; "Trypsin Gold", Promega).

After digestion, the samples were desalted by solid phase extraction in "Stage tips", self-made from polypropylene Vertex pipette tips (200 µL; SSIbio) filled with four layers of C18 reversed-phase excised from Empore 3M C18 extraction disks according to Rappsilber et al.[16] Desalted peptides were evaporated in Eppendorf Concentrator plus (Eppendorf) and dissolved in water/0.1% formic acid for further LC-MS/MS analysis. Each sample was analyzed in both DDA- and DIA-PASEF modes.

Library fractionation

To obtain a spectral library, we fractionated four pooled samples. For this purpose, we mixed all replicates across each biological group (VICs and osteoblasts in standard cultivation and after osteogenic differentiation). Then, 100 µg of each pooled sample was digested in-solution as described above and fractionated by Pierce™ High pH Reversed-Phase Peptide Fractionation Kit (ThermoFisher Scientific) according to manufacturer recommendations. Each fraction was desalted as described above and dissolved in water/0.1% formic acid. The peptide concentration was measured by Pierce™ Quantitative Fluorometric Peptide Assay (ThermoFisher Scientific) in Cary Eclipse Fluorescence Spectrometer (Agilent) according to manufacturer recommendations.

HPLC

Approximately 500 ng of tryptic peptides were used for shotgun proteomics analysis by nanoHPLC-MS/MS with trapped ion mobility mass spectrometry in timsTOF (Bruker). HPLC was performed in two-column separation mode with Acclaim™ PepMap™ 5 mm Trap Cartridge and Aurora Series separation column with nanoZero technology (C18, 25 cm x 75 µm ID, 1.6 µm C18) in gradient mode with 400 nL/min flow rate. Phase A was water/0.1% formic acid, phase B was acetonitrile/0.1% formic acid. The gradient was from 2% to 18% of phase B for 44 min, then to 25% of phase B for 11 min, to 37% of phase B for 5 min with subsequent wash with 95% of phase B for 17 min. The CaptiveSpray ion source was used for electrospray ionization with 1600 V of capillary voltage, 3 l/min N_2 flow, and 180 °C source temperature. MS acquisition was performed in two different modes.

DDA-PASEF

MS/MS acquisition in DDA-PASEF mode was used to analyze experimental and fractionated samples for the spectral library. The analysis was performed by automatic DDA-PASEF mode with 1.1 s cycle time and 10 PASEF ramps in positive polarity with the fragmentation of ions with at least two charges in m/z range from 100 to 1700 and ion mobility range from 0.60 to 1.60 1/K0. The active exclusion was enabled with a release time of 0.4 min and reconsideration after a four-fold increase in intensity. Collision energy was 20 eV for 0.6 1/K0 and 59 eV for 1.6 1/K0. Isolation width was 2 m/z for 700 m/z and 3 m/z for 800 m/z.

DIA-PASEF

Analysis in DIA-PASEF mode was performed in the same m/z and ion mobility range as DDA-PASEF. The DIA analysis was performed in positive polarity with the fragmentation of ions with at least two charges in m/z range from 100 to 1700 and ion mobility range from 0.60 to 1.60

1/K0. Cycle time was 1.6 s, and it included 16 fragmentation steps with 26 m/z and 0.01-0.02 1/K0 step (tab. 1). Each step included parallel fragmentation of two fragments of the spectra with a difference in 400 m/z and 0.3 1/K0. The collision energy was 20 eV for 0.85 1/K0 and 59 eV for 1.3 1/K0.

Table 1 DIA-PASEF isolation list

MS type	Cycle Id	Start IM [1/K0]	End IM [1/K0]	Start Mass [m/z]	End Mass [m/z]
MS1	0	-	-	-	-
PASEF	1	0.9001	1.2001	800.00	826.00
PASEF	1	0.6000	0.9001	400.00	426.00
PASEF	2	0.9201	1.2201	825.00	851.00
PASEF	2	0.6200	0.9201	425.00	451.00
PASEF	3	0.9301	1.2301	850.00	876.00
PASEF	3	0.6300	0.9301	450.00	476.00
PASEF	4	0.9500	1.2501	875.00	901.00
PASEF	4	0.6501	0.9500	475.00	501.00
PASEF	5	0.9600	1.2601	900.00	926.00
PASEF	5	0.6601	0.9600	500.00	526.00
PASEF	6	0.9800	1.2801	925.00	951.00
PASEF	6	0.6801	0.9800	525.00	551.00
PASEF	7	0.9900	1.2901	950.00	976.00
PASEF	7	0.6900	0.9900	550.00	576.00
PASEF	8	1.0101	1.3101	975.00	1001.00
PASEF	8	0.7100	1.0101	575.00	601.00
PASEF	9	1.0201	1.3201	1000.00	1026.01
PASEF	9	0.7200	1.0201	600.00	626.00
PASEF	10	1.0401	1.3401	1025.01	1051.01
PASEF	10	0.7400	1.0401	625.00	651.00
PASEF	11	1.0601	1.3601	1050.01	1076.01
PASEF	11	0.7601	1.0601	650.00	676.00
PASEF	12	1.0701	1.3701	1075.01	1101.01
PASEF	12	0.7701	1.0701	675.00	701.00
PASEF	13	1.0901	1.3901	1100.01	1126.01
PASEF	13	0.7901	1.0901	700.00	726.00
PASEF	14	1.1001	1.4001	1125.01	1151.01
PASEF	14	0.8001	1.1001	725.00	751.00
PASEF	15	1.1201	1.4201	1150.01	1176.01
PASEF	15	0.8200	1.1201	750.00	776.00
PASEF	16	1.1300	1.4301	1175.01	1201.01
PASEF	16	0.8300	1.1300	775.00	801.00

Data analysis

Protein identification in all cases was performed using the same *Homo sapiens* reference proteome (UP000005640, downloaded 27.06.2022) with contaminations included.

Analysis of DDA-PASEF data and spectral library generation was performed in FragPipe (v. 18.0) according to the default LFQ-MBR workflow. The search parameters were parent and

fragment mass error tolerance 15 ppm, protein and peptide FDR less than 1%, protease rule – strict trypsin (cleave after KR), and two possible missed cleavage sites. Cysteine carbamidomethylation was set as a fixed modification. Methionine oxidation and acetylation of protein N-term were set as variable modifications.

DIA-PASEF data analysis was performed in DIA-NN (v. 1.8.1.) in two variants – with empirically obtained spectral library from FragPipe (DIA) or with deep-learning predicted spectral library constructed in DIA-NN (DIA-ML) with the same search parameters. The search parameters were default with minor changes: parent and fragment mass error tolerance were set in automatic interference mode and were within 13-15 ppm for all samples, protein, and peptide FDR less than 1%, protease rule – strict trypsin (cleave after KR), two possible missed cleavage sites. Cysteine carbamidomethylation was set as a fixed modification. Methionine oxidation and acetylation of protein N-term were set as variable modifications.

2.3 RNA-seq transcriptomics

RNA isolation

RNA was isolated using ExtractRNA (Eurogene) using a standard phenol/chloroform extraction procedure according to the manufacturer's instructions. Briefly, cells were washed with PBS and lysed in a Petry dish by ExtractRNA for 5 minutes at room temperature. Then, lysates were transferred to microcentrifuge tubes and mixed with 1/5 of the volume of chloroform. The samples were incubated for 10 minutes and centrifuged (20 min, 12600g, 4°C). The aqueous phase was mixed with ½ of the initial ExtractRNA volume of ice-cold isopropanol. After incubation for 10 minutes, the samples were centrifuged (20 min, 12600g, 4°C). The precipitate was washed with 70% ethanol, air-dried at room temperature, and dissolved in water. RNA quality was assessed by agarose electrophoresis and Nanodrop spectrometry.

Next-generation sequencing

For RNA-seq library preparation, 500 ng of each sample was used with the "CORALL Total RNA seq Library Prep Kit" (Lexogen), which included poly-A RNA selection according to the manufacturer's recommendation. The quality of the obtained libraries was verified by capillary electrophoresis using a 4150 TapeStation (Agilent, USA). Finally, the libraries were sequenced on the Illumina NextSeq500 platform using single-end reads. All samples were run simultaneously. Raw reads are deposited in the NCBI SRA database with BioProject identifier PRJNA947173. RNA sequencing was carried out using the equipment of the Core Facility "Medical Genomics" (Tomsk NRMC) and the Tomsk Regional Common Use Center.

Data analysis

Quality control of the fastq files was performed using the FASTQC and MultiQC packages. Adapter sequences were removed by the Fastp package. Then, reads were aligned to the *Homo sapiens* reference genome GRCh38 using the STAR package, with further quantification by the QoRTs package.

2.4. Statistical analysis

We performed statistical data analysis to compare osteoblasts and VICs at each stage (before and after osteogenic differentiation). Proteomic and transcriptomic data were analyzed separately, and then their results were compared. Statistical analysis was performed using R.

RNA-seq data analysis

Regarding the RNA-sequencing data analysis, we examined six samples of osteoblasts (comprising three under standard culture conditions and three differentiated samples) and six samples of VICs (including three under standard culture conditions and three differentiated samples), each with two technical replicates. The RNA-sequencing count analysis was performed in R using the DESeq2 library per the standard algorithm[17]. The VICs and osteoblast samples were compared at each time point, encompassing the standard culture conditions (control) and at the 10th day of osteogenic differentiation. The technical replicates were consolidated using the collapseReplicates function from the DESeq2 package. During data filtering, genes with counts below 10 were excluded. Statistical analysis involved the Wald test integrated into the standard DESeq2 analysis algorithm. Differentially expressed genes were chosen based on $|\text{Log2 Fold Change}| > 1$ and adjusted $p\text{-value} < 0.05$ criteria. Data normalization entailed applying the rlog transformation. The sample clustering was executed through Principal Component Analysis (PCA), and the conversion of Entrez Gene identifiers was conducted using the bitr function sourced from the clusterProfiler library.[18]

Enrichment analysis was performed using the clusterProfiler library, drawing from the Reactome Pathways database[19] and human whole genome annotation org.Hs.eg.db. The differential gene expression results were visualized through various R packages, including pheatmap[20], ComplexHeatmap[21], EnhancedVolcano, and ggplot2[22]. Additionally, Venn diagrams were generated using the VennDiagram library[23] to illustrate the intersections among differentially expressed genes from VIC and osteoblast comparisons of varying types.

Proteomics data analysis

We performed data filtration and imputation of missing values with the NAguideR package for proteomic data analysis.[24] Proteins with missed values in more than half of samples in at least one biological group and proteins with a coefficient of variation higher than 0.8 were removed. Then, we selected the optimal method for missed values imputation using "classic criteria" which was the "Robust data imputation" approach ("Impseqrob").[25]

Differential expression analysis was performed similarly to RNA-seq data analysis described above, with minor differences. Specifically, we used quantile normalization instead of rlog transformation and limma instead of DESeq2 for proteomics data.[26]

For the qualitative comparative analysis (presence/absence of transcript/protein) of VICs and osteoblasts, their transcriptomes and proteomes were compared under native (control) and differentiating conditions. To compare the proteomes and transcriptomes, proteins/transcripts present in at least 5 out of 6 samples were selected. For the transcriptome comparison, the dataset also was filtered for genes with less than 10 counts. The comparison was performed separately for control and differentiating cells. Enrichment analysis was performed for the unique genes according to the Biological Processes Gene Ontology. Overlap between transcriptomes and proteomes was visualized using Venn diagrams.

Analyses

Various proteomics approaches give different proteomics coverage

To obtain maximal proteome coverage and estimate the effectiveness of various types of MS data acquisition on a timsTOF Pro platform for the real biological experiment, we collected three datasets for the comparison of osteogenic differentiation of valve interstitial cells (VICs) and osteoblasts: (1) classic data-dependent acquisition (DDA) in parallel accumulation serial fragmentation (PASEF) mode of ion mobility trapped mass-spectrometry, (2) data-independent acquisition (DIA) mass-spectrometry in PASEF mode with the identification of proteins based on empirically obtained spectral library in DDA-PASEF mode for pre-fractionated samples or (3) with the identification based on library-free search with spectral library predicted from protein sequences by machine learning in DIA-NN software (DIA-ML).

After data filtration, we included 2 563 proteins for DDA, 4 105 proteins for DIA, and 4 874 for the DIA-ML datasets for further analysis (supplementary materials 1). So, in our case, classic DIA gives 37.5% higher proteomics coverage than the DDA approach. Summarizing proteins, we found 5 405 proteins identified by at least one method and 2195 identified by all three. By RNA-seq data, we identified transcripts associated with 7977 genes. While we could not expect to have a full overlap of RNA and protein levels within dynamic biological processes as it was shown in our previous proteotranscriptomic study of VICs osteogenic differentiation where we utilized different methods for both RNA-seq and shotgun proteomics[27]. Nevertheless, as soon as RNA-seq is less biased by the RNA/protein abundance, we might expect for all shotgun proteomics dataset to have similar overlap with RNA-seq data. As expected, we observed similar number of unique proteins identified exclusively in DDA and DIA datasets (127 and 156 respectfully) while 6.4% (701 protein) of all proteins/transcripts identified were unique for DIA-ML and were not identified in RNA-seq data (fig. 2 a). This might be a sign of the weaker accuracy of this approach, which is expected.

Furthermore, all three proteomics datasets identified only 41% of proteins (fig. 2 b), 977 of which were not identified by RNA-seq (fig. 2 a). DIA should be able to identify more proteins, and we see 1593 proteins identified only in DIA and DIA-ML datasets. Nevertheless, we see 1002 proteins unique for DIA-ML data besides these DIA-unique proteins.

KEGG pathway enrichment analysis of proteins, unique for DIA-PASEF methods, demonstrates various functional groups, including several signaling pathways: ErbB, NOD-like receptor, T cell receptor, and phosphatidylinositol signaling system (fig. 2 c). Proteins, unique for DIA-ML data, are mainly associated with RNA and, therefore, with KEGG signaling pathways related to viral infections (fig. 2 d). Nonetheless, the term “Osteoclast differentiation” attracts attention in the context of osteogenic differentiation. In the DIA-ML dataset, this term includes several proteins important in the focus of our study: TAB2 (MAP3K7 binding protein 2), PIK3CB (phosphatidylinositol-4,5-bisphosphate 3-kinase catalytic subunit beta), PIK3R2 (phosphoinositide-3-kinase regulatory subunit 2), ITGB3 (integrin subunit beta 3), PPP3CB and PPP3CC (protein phosphatase 3 catalytic subunit beta and gamma), CTSK (cathepsin K), FOSL1 (FOS like 1, AP-1 transcription factor subunit), CHUK (a component of inhibitor of nuclear factor kappa B kinase complex), TRAF2 and TRAF6 (TNF receptor-associated factor 2 and 6). Still, we do not observed expression of MMP9 and ACP5, identified to be specific markers of osteoclast linage by scRNA-seq human bone marrow [28] and, as we showed before, we have not found the presence of osteoclast-associated surface antigens (CD34 and CD117) in the osteoblast cells used here.[29]

Despite such differences in proteome coverage, these methods seem similar in the quantitation of proteins identified by both methods. We see a good correlation between different methods in pairwise Pearson correlation. The average correlation for the same sample in different datasets is 0.87 (0.83 – 0.88) between DDA and DIA; 0.87 (0.84 – 0.88) between DDA and DIA-ML; 0.98 (0.97 – 0.98) between DIA and DIA-ML. Accordingly, there is a good correlation between Log_2 Fold Changes calculated in different datasets for pairwise comparisons of biological groups studied. The average correlation Log_2 Fold Changes in different datasets is 0.83 (0.8 – 0.87) between DDA and DIA; 0.83 (0.81 – 0.86) between DDA and DIA-ML; 0.91 (0.89 – 0.92) between DIA and DIA-ML. We want to emphasize the apparent correlation between Log2 Fold Changes for differentially expressed proteins and the complete absence of proteins with mutually exclusive expression in different datasets (fig. 2 e, supplementary materials 2). Still, similar to our previous study of VICs osteogenic differentiation utilizing multi-omics approach[27], we observe weak correlation of RNA-seq and proteomics data (supplementary materials 2). Taking into account the fact that our previous study was performed independently with different proteomics and transcriptomics platforms, these phenomena seem to characterize the biological object and not the quality of the data obtained.

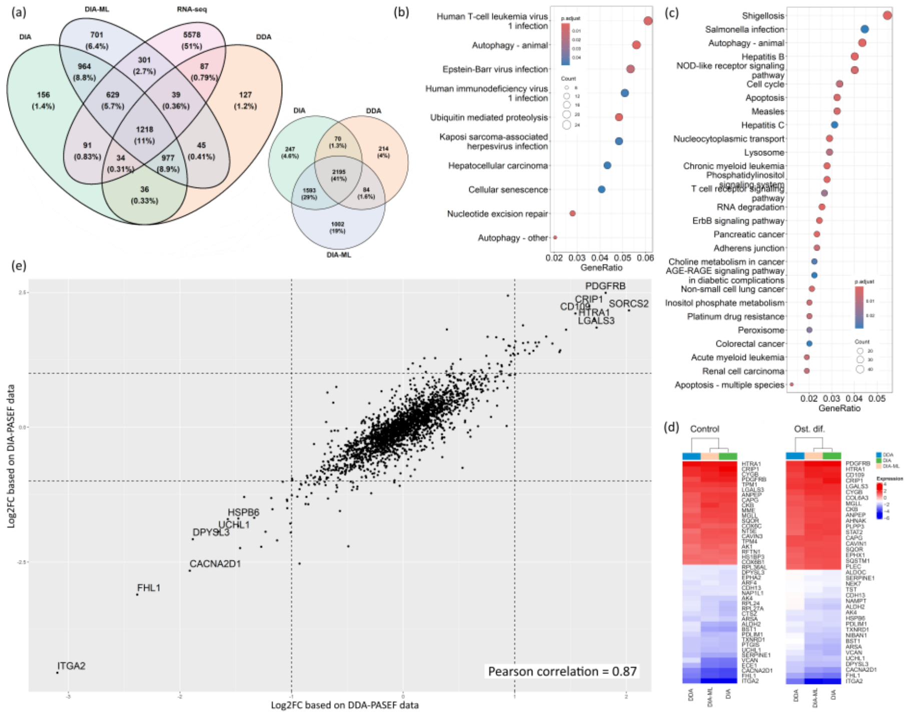

Figure 2 Comparison of three proteomics datasets (DDA, DIA, and DIA-ML) and RNA-seq data obtained for shotgun proteomics analysis of mechanisms of osteogenic differentiation of human valve interstitial cells (VICs) and osteoblasts. (a) Venn diagrams with overlap between gene products identified by RNA-seq in three proteomics datasets (left) and between gene products identified in three proteomics datasets (right). Proteomics datasets are represented after missed values filtration. (b, c) KEGG pathway enrichment analysis of proteins identified only by DIA-ML (b) or DIA (c). (d) Heatmap demonstrating the

Log$_2$ Fold Changes of differentially expressed genes, identified by all three proteomics methods between osteoblasts and differentiated VICs in standard cultivation (control) and after osteogenic differentiation (Ost. dif.). (e) Correlation of Log$_2$ Fold Changes for differentially expressed proteins compared to differentiated osteoblasts and VICs by DDA and DIA proteomics.

Proteomic and transcriptomic profiles of valve interstitial cells are distinct from osteoblasts both before and after osteogenic differentiation

In this work, we used osteoblast and valve interstitial cells (VICs) isolated by the standard protocol comprehensively characterized by us previously [29,30]. Osteoblasts were isolated from a femur of adult patients and represent the terminally differentiated cells expressing RUNX2, BGLAP, and COL1A1 even in standard cultivation as was shown before. Still, an osteogenic medium is necessary for extracellular matrix mineralization [29]. VICs were isolated from calcified aortic valve leaflets of adult patients, which makes them similar to osteoblasts - both cells were isolated from calcified tissues. Still, VICs do not express osteogenic markers and do not show ALP activity in standard cultivation [30,31].

To better characterize the cells studied we started from qualitative analysis (presence/absence) of transcriptome and proteome composition (focusing on DIA dataset as the most representative one). As expected, we observed significant differences in transcriptome composition between VICs and osteoblasts in standard cultivation, while proteome composition was more similar (Fig. 3 a; supplementary materials 3). Transcripts specific for osteoblasts are associated with extracellular matrix and collagen fibril organization. Proteomic data reinforce the importance of pathways such as ribosome biogenesis and RNA splicing (Fig. 3 b). In contrast, for VICs we observed unique signaling and protein processing transcripts (Fig. 3 c). These differences become increased even more after osteogenic differentiation (Fig. 3 d-f).

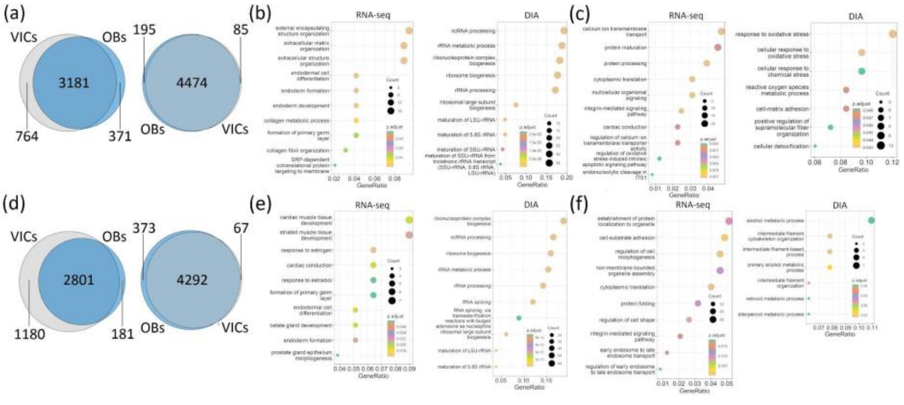

Figure 3. Comparative analysis of transcriptome (RNA-seq) and proteome (DIA) in valve interstitial cells (VICs) and osteoblasts (OBs) in standard cultivation (a-c) or after osteogenic differentiation (d-f). (a,d) Venn diagrams depicting the overlap of identified transcripts (left) or proteins (right). (b-c,e-f) Enrichment analysis based on "Biological processes" Gene Ontology for

genes (RNA-seq) and proteins (DIA) uniquely expressed in osteoblasts (b,e) or VICs (c,f) under standard cultivation (b-c) or after induction of osteogenic differentiation (e-f).

Using the four obtained datasets, we then performed quantitative analysis for proteins/transcripts being expressed across all samples. We compared the osteogenic differentiation of valve interstitial cells (pathological calcification) and osteoblasts (normal ossification). Clustering of the samples by principal component analysis (PCA) revealed a similar pattern for all datasets (supplementary materials 4) – VICs form distinct clusters before and after osteogenic differentiation and never cluster together with osteoblasts (fig. 4 a). Similar to our previous studies, osteoblasts form mixed clusters (fig. 4 a).[32,33]

For optimal data representation, we combined the results of the analysis of a differential expression to the three datasets: (1) differentially expressed transcripts identified by RNA-seq, (2) differentially expressed proteins identified in all three proteomics datasets (Proteomics reliable, "Prot. rel."), or (3) identified in at least one of the proteomics datasets (Proteomics wide, "Prot. w."). The datasets include 101, 20, and 162 genes upregulated in osteoblasts; 106, 23, and 208 genes upregulated in VICs, respectively, in standard cultivation. 102, 19, and 206 genes were upregulated in osteoblasts; 118, 20, and 191 genes were upregulated in VICs, respectively, after the induction of osteogenic differentiation.

In focusing on the gene ontology analysis in "Biological Processes", a limited intersection of differentially expressed genes is noticeable before and after the induction of osteogenic differentiation (fig. 4). Noteworthy is the Prot. rel dataset encompasses proteins identified via all three proteomic methods (DIA, DDA, and DIA-ML). In osteoblasts, this dataset shows overlaps in the biological processes of fibroblast activation and positive regulation of calcium ion import. In VICs, a more diverse array of processes is observed, primarily associated with chemotaxis, cell adhesion to the extracellular matrix, and regulating these processes.

Under standard culture conditions (control), osteoblasts exhibit the activation of actin filament assembly and organization processes, fibroblast activation, and cellular response to nutrients. In VICs under the same conditions, genes are enriched in lamellipodium assembly and organization, mesoderm formation, cell-matrix adhesion, and the positive regulation of chemotaxis. Differentiated osteoblasts show an upregulation of TGF-beta signaling and non-canonical Wnt signaling, an increase in collagen metabolism, and the activation of fibroblasts (fig. 4b). In differentiated VICs, these processes are absent, and the enriched pathways coincide with the pathways identified in undifferentiated VICs (fig. 4c). It is noteworthy that only about 30% of differentially expressed genes significantly differ between both control and differentiated VICs and osteoblasts. At the same time, about 60% of the genes are differentially expressed between these cell types only before or after osteogenic differentiation (supplementary materials 4). Notably, there is a similarity between the gene ontology analysis for cell-type specific (Fig. 3) and differentially expressed (Fig. 4) transcripts/proteins. Particularly, both types of analyses identify genes associated with cytoplasmic translation, integrin-mediated signaling pathway, and regulation of cell matrix adhesion for VICs; while for osteoblasts, the similarity is associated with various extracellular matrix-related terms.

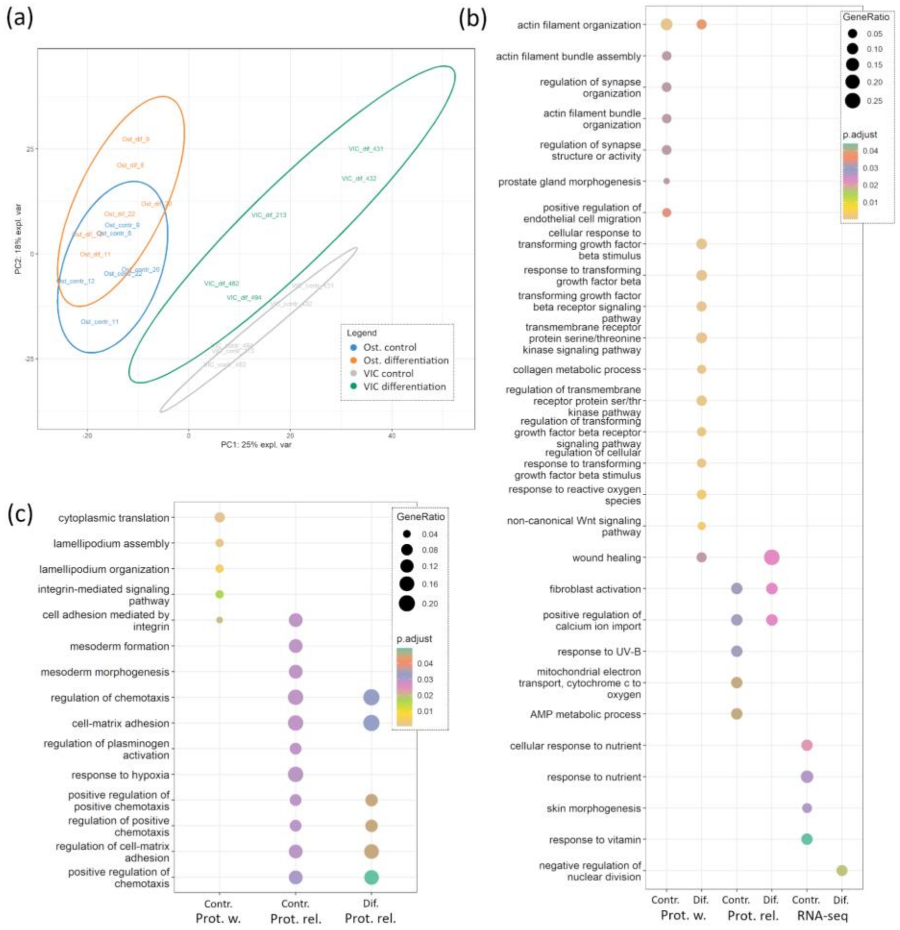

Figure 4 Comparison of human valve interstitial cells (VICs) and osteoblasts (Ost) cultured in standard conditions or after induction of osteogenic differentiation. (a) Clusterization of the samples in the principal component analysis (PCA) dimension based on DIA-PASEF data. Gray – VICs in standard cultivation; Green – VICs on the 10th day after induction of osteogenic differentiation; Blue – osteoblasts in standard cultivation; Yellow – osteoblasts on the 10th day after induction of osteogenic differentiation. (b) Proteins or transcripts upregulated in osteoblasts during standard cultivation (Contr.) or on the 10th day after induction of osteogenic differentiation (Dif.). (c) Proteins or transcripts upregulated in VICs during standard cultivation (contr.) or on the 10th day after induction of osteogenic differentiation (Dif.). RNA-seq – transcripts identified by RNA-seq; Prot. w. – differentially expressed proteins, identified by at least one shotgun proteomics dataset; Prot. rel. – differentially expressed proteins identified by all three shotgun proteomics datasets.

We found a low overlap between proteomics and RNA-seq data, which was expected from our previous proteotranscriptomics dataset of VICs osteogenic differentiation (fig. 4).[27] Table 2 lists gene products (proteins or transcripts) up- or downregulated between osteoblasts and VICs in both proteomics and RNA-seq data.

Table 2 Genes, products of which are up- or downregulated in both RNA-seq and one of the proteomic datasets in comparisons of osteoblasts and valve interstitial cells (VICs) in standard cultivation conditions or on the 10th day of osteogenic differentiation.

	Standard cultivation	Osteogenic differentiation
Upregulated in osteoblasts	**TGFBI**, **SULF1**, GALNT1, CALD1, POSTN	**TGFBI**, SERPINE2, COL8A1
Upregulated in VICs	**VCAN**, **EFEMP1**, **ANK3**, **CCDC80**, CACNA2D1	**VCAN**, **EFEMP1**, **ANK3**, **CCDC80**, HEG1

One of the differences between transcriptomic and proteomic datasets is that in the RNA-seq data, we found a surprisingly high proportion of differentially expressed long non-coding RNA (lncRNA). Most of these lncRNAs are known antisense RNA (tab. 3). Notably, many of these antisense RNAs significantly differ only before but not after induction of osteogenic differentiation (tab. 3).

Table 3 Annotated antisense RNA found in transcriptomes of osteoblasts in comparison with valve interstitial in standard cultivation conditions or on the 10th day of osteogenic differentiation. Non-significant – adjusted P-value > 0.05

Upregulated in osteoblasts						
Name	ENSEMBL	Std. cultivation		Ost. differentiation		Target gene
		Log_2 FC	adj. p.val.	Log_2 FC	adj. p.val.	
HOXA-AS3	ENSG00000254369	8.3	4.82E-09	7.7	2.02E-07	HOXA
HOXA10-AS	ENSG00000253187	8.2	9.84E-09	7.1	1.71E-06	HOXA10
DLX6-AS1	ENSG00000231764	6.0	2.76E-04	7.0	1.86E-05	DLX6
HOXA11-AS	ENSG00000240990	6.8	2.38E-05	5.9	1.93E-03	HOXA11
Lnc-FIGNL2-6	ENSG00000260122	5.8	7.42E-04	5.7	1.06E-03	HSALNG0091106
CHL1-AS1	ENSG00000234661	6.2	3.47E-04	5.6	2.38E-03	CHL1
Lnc-RD3-11	ENSG00000279333	7.6	5.50E-07	5.6	1.32E-03	KCNH1
PENK-AS1	ENSG00000254254	5.2	4.38E-04	5.4	9.61E-08	PENK
HOXD-AS2	ENSG00000237380	non-significant		5.2	2.55E-02	HOXD
Lnc-DAOA-47	ENSG00000284966	4.1	2.01E-10	5.1	7.12E-02	EFNB2
Lnc-SLC25A32-9	ENSG00000253851	6.5	6.51E-07	non-significant		BAALC
Upregulated in valve interstitial cells						
Name	ENSEMBL	Std. cultivation		Ost. differentiation		Target gene
		Log_2 FC	adj. p.val.	Log_2 FC	adj. p.val.	
Lnc-COPG2-3	ENSG00000270823	1.8	5.13E-07	6.4	2.24E-05	MEST
LAMA5-AS1	ENSG00000228812	non-significant		5.1	8.59E-03	LAMA5

CAVIN2-AS1	ENSG00000233766	3.5	3.94E-02	5.0	1.08E-02	CAVIN2
ANK3-AS	ENSG00000232682	3.6	1.73E-19	4.9	3.14E-37	ANK3
CADM3-AS1	ENSG00000225670	6.6	8.84E-03	non-significant		CADM3
CCDC110-AS	ENSG00000249679	6.07	1.34E-04	4.3	3.23E-02	CCDC110
ADGRD1-AS1	ENSG00000256151	6.0	5.02E-04	non-significant		ADGRD1
Lnc-THNSL1-4	ENSG00000273107	5.9	9.12E-04	non-significant		THNSL1
HAND2-AS1	ENSG00000237125	5.1	5.05E-09	2.5	1.36E-04	HAND2
ENSG00000289061	ENSG00000289061	5.1	1.84E-02	non-significant		EMILIN2

Discussion

DIA-PASEF with library-free search is optimal for routine shotgun proteomics

The rapid advancements in shotgun proteomic techniques present a challenge for end users, particularly those who may need to be more experts in proteomics, to determine the most suitable approach for their specific needs. Furthermore, multiple data acquisition modes are available within a single proteomics platform, making it challenging to select the optimal one.

Trapped Ion Mobility Spectrometry (TIMS), utilized in the timsTOF Pro platform, stands out as one of the leading technologies for routine shotgun proteomics. It is particularly valuable for analyses involving limited sample amounts or the need to study large cohorts, as often found in biomedical research. However, comprehensive comparisons of different data acquisition and analysis strategies are scarce from a user's perspective, particularly those conducted within actual biological objectives and complemented by transcriptomics data.

In this context, we have conducted a comparative study of two major proteomic approaches on the timsTOF Pro platform: Data Dependent Acquisition (DDA) and Data Independent Acquisition (DIA) with Parallel Accumulation-Serial Fragmentation (PASEF). We used one of the most modern approaches for data analysis, but still available in a user-friendly interface: MSFragger for DDA-PASEF and DIA-NN for DIA-PASEF data [34,35].

Our biological aim was to compare VICs and osteoblast osteogenic differentiation *in vitro*. Our samples exhibit greater sophistication than the HeLa samples typically utilized in comparative proteomics research as test samples. This sophistication arises from three key factors: (i) The primary function of both VICs and osteoblasts is the secretion of extracellular matrix (ECM). As a result, their proteomes are enriched with numerous ECM proteins, which interfere with identifying less abundant proteins, thereby limiting proteome coverage. (ii) The primary cell cultures in our study are derived from donors with diverse genetic backgrounds and lifestyle factors, introducing a more comprehensive range of biological variability compared to studies using uniform cell lines like HeLa. (iii) The availability of a limited number of biological replicates is a common constraint in most biomedical studies, including ours. This limitation can affect the statistical power and reproducibility of the findings.

As a result, our study quantified half as many protein groups as those reported for HeLa cells.[13] However, DIA-PASEF methodologies proved to be more advantageous in our context. Specifically, DIA-NN library-free search and the conventional library-based DIA analysis increased

the quantification of protein groups to 90% and 60%, respectively. A similar dramatic increase in performance was observed in the comparison of DDA- and DIA-PASEF methods for metaproteomics, which possess even more technical challenges than we faced in our study. In the study of Gómez-Varela et al., DIA-PASEF resulted in the quantification of up to 4 times more taxonomic units than DDA-PASEF.[12] This contrasts with the findings of Huang et al., who reported only 30% increase in quantified proteins for HeLa samples using a similar DDA- and DIA-PASEF comparative analysis.

To evaluate the reliability of our protein identification, we compared the identified proteins with the transcripts detected through RNA-seq transcriptomics. The inherent discrepancies between RNA-seq and proteomics data are well known. It is generally assumed that this phenomenon comes from the variation in translation and protein degradation rates.[36,37] While the exact reason for the poor correlation of mRNA/protein abundance is hard to define, much phenomenological data has been accumulated. Notably, Gygi et al. (1999) represent one of the classic works on yeasts regarding this problem. They showed that the correlation between mRNA and protein abundances cannot predict protein expression. Moreover, the mRNA levels were stable for some genes, but the protein abundances varied more than 20-fold and vice versa.[38] No wonder that in human cells and tissues, we also observe a poor correlation between protein and mRNA abundances, which might be clearly seen in the deep proteome and transcriptome atlas of healthy human tissues.[39] Hundreds of proteins could not be detected even for highly expressed mRNAs. At the same time, protein expression is often more stable across tissues than that of transcripts, which correlates well with our observation regarding higher differences in transcriptome composition between VICs and osteoblasts compared to lower proteome variability (Fig. 3). The mRNA/protein correlation might be especially low for specialized and terminally differentiated tissue, e.g. Moritz et al. (2019) revealed poor to moderate mRNA/protein correlation varying from 0.34 to 0.54 in brain.[40] Similarly, our previous study observed a low correlation between RNA-seq and shotgun proteomic data for various mesenchymal cell primary cultures [33] and VICs [27]. Therefore, we expect a limited correlation between transcriptomic and proteomic data here.

Still, comparing the RNA-seq data with various proteomics datasets obtained for the same samples might be used to roughly assess various PASEF techniques. Notably, the highest number of unique proteins was observed with DIA-PASEF data analyzed through a library-free approach, accounting for only 6.4% of all proteins identified (Fig. 2a). Meanwhile, traditional DIA-PASEF using a library-based search yielded the most balanced results in terms of both high protein quantification and methodological overlap. This finding aligns well with existing literature recognizing library-based searches as exceptionally reliable for analyzing DDA and DIA data.[41]

Achieving nearly a two-fold increase in the number of proteins identified is critical for the downstream identification of differentially expressed proteins. This significance is underscored by the fact that highly abundant proteins are often housekeeping proteins, which are remarkably stable from both evolutionary and functional perspectives.[42,43] In our study, this enhanced identification capability has enabled us to detect hundreds of proteins associated with key cellular compartments (such as peroxisomes and lysosomes) and processes (including endocytosis and apoptosis), as well as numerous signaling pathways, such as ErbB, NOD-like receptor, phosphatidylinositol, T-cell receptor, and EGFR (Fig. 2b-c). As a result, we significantly increased the detection of differentially expressed proteins (DEPs), identifying 371 DEPs in DDA

datasets and 646 DEPs in DIA datasets when comparing valve interstitial cells (VICs) and osteoblasts after osteogenic differentiation as an example (Supplementary Materials 1).

Further in-depth comparison of DEPs identified by different PASEF protocols revealed a surprisingly high correlation in DEP identification across various methods. The correlation of Log2 Fold Changes (Log2FC) between different approaches within all biological comparisons ranged from 0.8 to 0.92. We encountered no DEPs with opposing directions across the datasets (Fig. 2d-e, Supplementary Materials 2). The highest correlation was observed between DIA and DIA-ML approaches (0.91), indicating a robust consistency in our data.

In summary, our observations have shown that the DIA-PASEF method offers a significantly greater advantage over the DDA approach for complex samples in actual proteomics studies, surpassing the performance observed with HeLa test samples. While the library-based search in DIA-PASEF sets a high standard in shotgun proteomics, the DIA-NN-based library-free search presents a more accessible alternative. The library-free search, facilitated by the user-friendly DIA-NN software, rivals the library-based approach in terms of differentially expressed proteins (DEPs) identified, and it only exhibits a slight reduction in data reliability. Our findings suggest that for routine shotgun proteomics tasks on the timsTOF Pro platform, DIA-PASEF with a library-free search emerges as the preferable method.

Specificity of VICs osteogenic differentiation offers potential for target CAVD therapy

The comprehensive multi-omics dataset discussed in detail above allows us to investigate the differences in molecular mechanisms of normal osteogenic differentiation in osteoblasts and pathological osteogenic differentiation in valvular interstitial cells (VICs).

Calcified aortic valves, particularly in the later stages of calcific aortic valve disease (CAVD), are often presumed to exhibit a high degree of similarity to lamellar bone regarding calcium crystal structure and histological organization. However, upon closer examination, the structures akin to mature bone are only observed in a minority of the CAVD cases.[44] Consequently, our observations reveal significant differences in the mechanisms of osteogenic differentiation between osteoblast-like cells derived from adult bone and VICs isolated from calcified aortic valves in the CAVD latter stages. As previously demonstrated, the osteoblast proteome does not undergo significant changes during osteogenic differentiation.[32,33] In contrast, the VICs' proteome exhibits notable alterations (Supplementary Materials 4).[27] Before and following differentiation, VICs express higher levels of proteins related to chemotaxis, cell-matrix adhesion, and mesodermal markers. Conversely, osteoblasts show an increased expression of proteins involved in osteogenic differentiation, such as those associated with TGF-beta and Wnt signaling pathways, collagen synthesis, and calcium ion import. Given the endothelial origins of VICs[7], these differences align well with previous studies that have documented varied osteogenic differentiation mechanisms in cells of different origins. [9,30,33,45–47] Furthermore, research by Nantavisai et al. (2020) suggests that these distinctions could be leveraged for the selective inhibition of osteogenic differentiation in specific cell types *in vitro*.[45] From this perspective, proteins exclusively upregulated during VICs' osteogenic differentiation present promising targets for selective anti-CAVD treatments (supplementary materials 5).

We found an overexpression of Monoamine oxidase A (MAOA) during osteogenic differentiation of valvular interstitial cells (VICs) compared to osteoblasts. Recognized as a

critical marker for CAVD, MAOA has been identified as a potential treatment for this disease.[48] Importantly, our data highlight its selectivity in targeting pathological valve calcification, enhancing its therapeutic promise. Additionally, we observed an upregulation of A-Kinase Anchoring Protein 2 (AKAP2) exclusively during VICs' osteogenic differentiation. Previous studies have linked AKAP2 mutations with adolescent idiopathic scoliosis and demonstrated the critical role of the AKAP2/ERK1/2 signaling axis in longitudinal bone growth.[49,50] The specific upregulation of AKAP2 in VICs—unlike in osteoblasts—suggests its association with the early stages of osteogenic differentiation in VICs. Similarly, another protein upregulated in VICs differentiation, GLIPR2, also modulates ERK1/2 signaling, though its role in osteogenic differentiation has not been previously described.[51] The p38 mitogen-activated protein kinase (MAPK) signaling pathway, which includes ERK1/2, is involved in inflammation and is well-known to be associated with atherosclerotic and aortic valve calcification.[52] Moreover, it was shown that andrographolide inhibits VICs calcification via the NF-kappa B/Akt/ERK pathway *in vitro*, which supports our data regarding ERK1/2 to be a promising target for selective therapy against aortic valve calcification.[53]

Limitations of the study

Finally, we would like to emphasize the limitations of the study. First, we cultured primary cell cultures for several passages *in vitro* due to the high amount of samples needed. For this, we used a well-defined methodology [9, 27,29,30,33], guaranteeing that we worked with well-defined cell types but also limited our observation relative to the native tissue. Still, as discussed above, many of our observations correlate well with the data from the whole-tissue models. Other significant limitations are associated with general poor proteomics reproducibility and rapid development of data analysis tools. Particularly, Varnavides et al. (2022) compared 16 shotgun proteomics sample preparation methods and revealed method-dependent differences in specific protein features identification.[54] Beyond sample preparation, the data-dependent acquisition approach used by us for the DDA dataset and spectral library preparation is also known to have limited reproducibility due to stochastic sampling.[55] Finally, the software used in this study was significantly improved even during the time between submitting and publishing of the presented manuscript (especially in the case of library-free DIA workflow).

Supplementary Materials

Supplementary Materials 1: Results of differential expression analysis between VICs and osteoblasts in control and osteogenic differentiation for the three proteomics and RNA-seq datasets; Supplementary Materials 2: Correlation of Log2 Fold changes for differentially expressed proteins between various proteomic datasets and proteomic datasets with RNA-seq data; Supplementary Materials 3: Results of qualitative analysis (presence/absence) of protein/transcripts between VICs and osteoblasts in standard cultivation and after osteogenic differentiation (separate xlsx file); Supplementary Materials 4: Clustering of osteoblasts and human valve interstitial cells (VICs) samples by principal component analysis (PCA); Supplementary Materials 5: Venn diagrams representing the overlap of differentially expressed genes and proteins; Supplementary Materials 6: Boxplots for selective proteins

Data Availability

The mass spectrometry proteomics data have been deposited to the ProteomeXchange Consortium via the PRIDE[56] partner repository with the dataset identifier PXD036566. Raw reads are deposited in the NCBI SRA database with BioProject identifier PRJNA947173. All additional supporting data are available in the *GigaScience* repository, GigaDB [57].

Reproducible codes for data analysis in R are available at https://github.com/tniapp/Human-VICs-and-osteoblasts-osteogenic-differentiation.

Funding

The study was supported by a grant from the Russian Science Foundation 23-15-00320

Authors' contributions

A.L., P.K., I.T., A.I. performed data analysis; A.L., N.B., D.P., I.K., E.R., E.D., T.G. performed laboratory experiments; N.B., D.P., D.K., V.U., R.T., S.B., V.K. collected donor material, isolated primary cell cultures, performed cell cultivation and differentiation experiments; C.W. and K.X. project design consulting and manuscript editing; A.M. supervised the study; A.L., P.K., and A.M. drafted the manuscript. All authors read and approved the final manuscript.

Competing Interests

All authors declare no conflict of interest.

Acknowledgment

Shotgun proteomics analysis was carried out using the equipment of the Core Facility “Centre for Molecular and Cell Technologies” (St. Petersburg State University). RNA sequencing was performed on the Core Facility “Medical Genomics” (Tomsk NRMC) equipment and the TomskRegional Common Use Center.

References

1. Grosso A, Burger MG, Lunger A, Schaefer DJ, Banfi A, Di Maggio N. It Takes Two to Tango: Coupling of Angiogenesis and Osteogenesis for Bone Regeneration. *Front Bioeng Biotechnol*. 2017; doi: 10.3389/fbioe.2017.00068.

2. Rennenberg R, Kessels, Schurgers, Van Engelshoven, De Leeuw P, Kroon. Vascular calcifications as a marker of increased cardiovascular risk: A meta-analysis. *Vasc Health Risk Manag*. 2009; doi: 10.2147/VHRM.S4822.

3. Yi B, Zeng W, Lv L, Hua P. Changing epidemiology of calcific aortic valve disease: 30-year trends of incidence, prevalence, and deaths across 204 countries and territories. *Aging*. 2021; doi: 10.18632/aging.202942.

4. Coffey S, Roberts-Thomson R, Brown A, Carapetis J, Chen M, Enriquez-Sarano M, et al.. Global epidemiology of valvular heart disease. *Nat Rev Cardiol*. 2021; doi: 10.1038/s41569-021-00570-z.

5. Aikawa E, Hutcheson JD, editors. Cardiovascular Calcification and Bone Mineralization. Cham: Springer International Publishing; 2020. https://doi.org/10.1007/978-3-030-46725-8

6. Theodoris CV, Zhou P, Liu L, Zhang Y, Nishino T, Huang Y, et al.. Network-based screen in iPSC-derived cells reveals therapeutic candidate for heart valve disease. *Science*. 2021; doi: 10.1126/science.abd0724.

7. Rutkovskiy A, Malashicheva A, Sullivan G, Bogdanova M, Kostareva A, Stensløkken K, et al.. Valve Interstitial Cells: The Key to Understanding the Pathophysiology of Heart Valve Calcification. *J Am Heart Assoc*. 2017; doi: 10.1161/JAHA.117.006339.

8. Blaser MC, Kraler S, Lüscher TF, Aikawa E. Multi-Omics Approaches to Define Calcific Aortic Valve Disease Pathogenesis. *Circ Res*. 2021; doi: 10.1161/CIRCRESAHA.120.317979.

9. Lobov A, Malashicheva A. Osteogenic differentiation: a universal cell program of heterogeneous mesenchymal cells or a similar extracellular matrix mineralizing phenotype? *Biol Commun*. 2022; doi: 10.21638/spbu03.2022.104.

10. Guergues J, Wohlfahrt J, Stevens SM. Enhancement of Proteome Coverage by Ion Mobility Fractionation Coupled to PASEF on a TIMS–QTOF Instrument. *J Proteome Res*. 2022; doi: 10.1021/acs.jproteome.2c00336.

11. Fernández-Vega A, Farabegoli F, Alonso-Martínez MM, Ortea I. Comparison of TIMS-PASEF quantitative proteomics data-analysis workflows using FragPipe, DIA-NN, and Spectronaut from a user's perspective. bioRxiv. 2021. doi: https://doi.org/10.1101/2021.11.29.470373

12. Gómez-Varela D, Xian F, Grundtner S, Sondermann JR, Carta G, Schmidt M. Increasing taxonomic and functional characterization of host-microbiome interactions by DIA-PASEF metaproteomics. *Front Microbiol*. 2023; doi: 10.3389/fmicb.2023.1258703.

13. Huang Z, Kong W, Wong BJ, Gao H, Guo T, Liu X, et al.. Proteomic datasets of HeLa and SiHa cell lines acquired by DDA-PASEF and diaPASEF. *Data Brief*. 2022; doi: 10.1016/j.dib.2022.107919.

14. Wang H, Lim KP, Kong W, Gao H, Wong BJH, Phua SX, et al.. MultiPro: DDA-PASEF and diaPASEF acquired cell line proteomic datasets with deliberate batch effects. *Sci Data*. 2023; doi: 10.1038/s41597-023-02779-8.

15. Janetzko K, Rink G, Hecker A, Bieback K, Klüter H, Bugert P. A Single-Tube Real-Time PCR Assay for Mycoplasma Detection as a Routine Quality Control of Cell Therapeutics. *Transfus Med Hemotherapy*. 2014; doi: 10.1159/000357096.

16. Rappsilber J, Mann M, Ishihama Y. Protocol for micro-purification, enrichment, pre-fractionation and storage of peptides for proteomics using StageTips. *Nat Protoc*. 2007; doi: 10.1038/nprot.2007.261.

17. Love MI, Huber W, Anders S. Moderated estimation of fold change and dispersion for RNA-seq data with DESeq2. *Genome Biol*. 2014; doi: 10.1186/s13059-014-0550-8.

18. Yu G, Wang L-G, Han Y, He Q-Y. clusterProfiler: an R Package for Comparing Biological Themes Among Gene Clusters. *OMICS J Integr Biol*. 2012; doi: 10.1089/omi.2011.0118.

Field Code Changed

19. Yu G, He Q-Y. ReactomePA: an R/Bioconductor package for reactome pathway analysis and visualization. *Mol Biosyst*. 2016; doi: 10.1039/C5MB00663E.

20. Kolde R. pheatmap: Pretty Heatmaps. Github. 2018. https://github.com/raivokolde/pheatmap

21. Gu Z. Complex heatmap visualization. *iMeta*. 2022; doi: 10.1002/imt2.43.

22. Wickham H. ggplot2. Cham: Springer International Publishing; 2009; DOI: https://doi.org/10.1007/978-0-387-98141-3.

23. Chen H, Boutros PC. VennDiagram: a package for the generation of highly-customizable Venn and Euler diagrams in R. *BMC Bioinformatics*. 2011; doi: 10.1186/1471-2105-12-35.

24. Wang S, Li W, Hu L, Cheng J, Yang H, Liu Y. NAguideR: performing and prioritizing missing value imputations for consistent bottom-up proteomic analyses. *Nucleic Acids Res*. 2020; doi: 10.1093/nar/gkaa498.

25. Branden KV, Verboven S. Robust data imputation. *Comput Biol Chem*. 2009; doi: 10.1016/j.compbiolchem.2008.07.019.

26. Ritchie ME, Phipson B, Wu D, Hu Y, Law CW, Shi W, et al.. limma powers differential expression analyses for RNA-sequencing and microarray studies. *Nucleic Acids Res*. 2015; doi: 10.1093/nar/gkv007.

27. Semenova D, Zabirnyk A, Lobov A, Boyarskaya N, Kachanova O, Uspensky V, et al.. Multi-omics of in vitro aortic valve calcification. *Front Cardiovasc Med*. 2022; doi: 10.3389/fcvm.2022.1043165.

28. Lee NY, Li M, Ang KS, Chen J. Establishing a human bone marrow single cell reference atlas to study ageing and diseases. *Frontiers in Immunology*. 2023; doi: 10.3389/fimmu.2023.1127879

29. Kostina D, Lobov A, Klausen P, Karelkin V, Tikhilov R, Bozhkova S, etal.. Isolation of human osteoblast cells capable for mineralization and synthetizing bone-related proteins in vitro from adult bone. *Cells*. 2022; doi: 10.3390/cells11213356

30. Kostina A, Lobov A, Semenova D, Kiselev A, Klausen P, Malashicheva A. Context-Specific Osteogenic Potential of Mesenchymal Stem Cells. *Biomedicines*. 2021; doi: 10.3390/biomedicines9060673.

31. Osman L, Yacoub MH, Latif N, Amrani M, Chester AH. Role of human valve interstitial cells in valve calcification and their response to atorvastatin. *Circulation*. 2006; doi: 10.1161/CIRCULATIONAHA.105.001115

32. Khvorova IA, Kostina DA, Zainullina BR, Fefilova EA, Gromova ES, Tikhilov RM, et al.. Osteogenic Differentiation In Vitro of Human Osteoblasts Is Associated with Only Slight Shift in Their Proteomics Profile. *Cell Tissue Biol*. 2022; doi: 10.1134/S1990519X22060025.

33. Lobov A, Kuchur P, Khizhina A, Kotova A, Ivashkin A, Kostina D, et al.. Mesenchymal Cells Retain the Specificity of Embryonal Origin During Osteogenic Differentiation. *Stem Cells*. 2024; doi: 10.1093/stmcls/sxad081.

34. Yu F, Haynes SE, Teo GC, Avtonomov DM, Polasky DA, Nesvizhskii AI. Fast Quantitative Analysis of timsTOF PASEF Data with MSFragger and IonQuant. *Mol Cell Proteomics*. 2020; doi: 10.1074/mcp.TIR120.002048.

35. Demichev V, Messner CB, Vernardis SI, Lilley KS, Ralser M. DIA-NN: neural networks and interference correction enable deep proteome coverage in high throughput. *Nat Methods*. 2020; doi: 10.1038/s41592-019-0638-x.

36. Kristensen AR, Gsponer J, Foster LJ. Protein synthesis rate is the predominant regulator of protein expression during differentiation. *Molecular systems biology*. 2013; doi: 10.1038/msb.2013.47

37. Lian X, Guo J, Gu W, Cui Y, Zhong J, Jin J, et al.. Genome-wide and experimental resolution of relative translation elongation speed at individual gene level in human cells. *PLoS genetics*. 2016; doi: 10.1371/journal.pgen.1005901

38. Gygi SP, Rochon Y, Franza BR, Aebersold R. Correlation between protein and mRNA abundance in yeast. *Molecular and cellular biology*. 1999; doi: 10.1128/mcb.19.3.1720

39. Wang D, Eraslan B, Wieland T, Hallström B, Hopf T, Zolg DP, et al.. A deep proteome and transcriptome abundance atlas of 29 healthy human tissues. *Molecular systems biology*. 2019; doi: 10.15252/msb.20188503

40. Moritz CP, Mühlhaus T, Tenzer S, Schulenborg T, Friauf E. Poor transcript-protein correlation in the brain: negatively correlating gene products reveal neuronal polarity as a potential cause. *Journal of neurochemistry*. 2019; doi: 10.1111/jnc.14664

41. Fernández-Costa C, Martínez-Bartolomé S, McClatchy DB, Saviola AJ, Yu N-K, Yates JR. Impact of the Identification Strategy on the Reproducibility of the DDA and DIA Results. *J Proteome Res*. 2020; doi: 10.1021/acs.jproteome.0c00153.

42. Bédard C, Cisneros AF, Jordan D, Landry CR. Correlation between protein abundance and sequence conservation: what do recent experiments say? *Curr Opin Genet Dev*. 2022; doi: 10.1016/j.gde.2022.101984.

43. Ivanic J, Yu X, Wallqvist A, Reifman J. Influence of Protein Abundance on High-Throughput Protein-Protein Interaction Detection. Shakhnovich EI, editor. *PLoS ONE*. 2009; doi: 10.1371/journal.pone.0005815.

44. Mohler ER, Gannon F, Reynolds C, Zimmerman R, Keane MG, Kaplan FS. Bone Formation and Inflammation in Cardiac Valves. *Circulation*. 2001; doi: 10.1161/01.CIR.103.11.1522.

45. Nantavisai S, Pisitkun T, Osathanon T, Pavasant P, Kalpravidh C, Dhitavat S, et al.. Systems biology analysis of osteogenic differentiation behavior by canine mesenchymal stem cells derived from bone marrow and dental pulp. *Sci Rep*. 2020; doi: 10.1038/s41598-020-77656-0.

46. Dadras M, May C, Wagner JM, Wallner C, Becerikli M, Dittfeld S, et al.. Comparative proteomic analysis of osteogenic differentiated human adipose tissue and bone marrow-derived stromal cells. *J Cell Mol Med*. 2020; doi: 10.1111/jcmm.15797.

47. Kotova AV, Lobov AA, Dombrovskaya JA, Sannikova VY, Ryumina NA, Klausen P, et al.. Comparative Analysis of Dental Pulp and Periodontal Stem Cells: Differences in Morphology,

Functionality, Osteogenic Differentiation and Proteome. *Biomedicines*. 2021; doi: 10.3390/biomedicines9111606.

48. Decano JL, Iwamoto Y, Goto S, Lee JY, Matamalas JT, Halu A, et al.. A disease-driver population within interstitial cells of human calcific aortic valves identified via single-cell and proteomic profiling. *Cell Rep*. 2022; doi: 10.1016/j.celrep.2022.110685.

49. Li W, Li Y, Zhang L, Guo H, Tian D, Li Y, et al.. *AKAP2* identified as a novel gene mutated in a Chinese family with adolescent idiopathic scoliosis. *J Med Genet*. 2016; doi: 10.1136/jmedgenet-2015-103684.

50. Wang B, Jiang B, Li Y, Dai Y, Li P, Li L, et al.. AKAP2 overexpression modulates growth plate chondrocyte functions through ERK1/2 signaling. *Bone*. 2021; doi: 10.1016/j.bone.2021.115875.

51. Huang S, Liu F, Niu Q, Li Y, Liu C, Zhang L, et al.. GLIPR-2 Overexpression in HK-2 Cells Promotes Cell EMT and Migration through ERK1/2 Activation. Hotchin NA, editor. *PLoS ONE*. 2013; doi: 10.1371/journal.pone.0058574.

52. Reustle A, Torzewski M. Role of p38 MAPK in Atherosclerosis and Aortic Valve Sclerosis. *Int J Mol Sci*. 2018; doi: 10.3390/ijms19123761.

53. Huang Y, Zhou X, Liu M, Zhou T, Shi J, Dong N, et al.. The natural compound andrographolide inhibits human aortic valve interstitial cell calcification via the NF-kappa B/Akt/ERK pathway. *Biomed Pharmacother*. 2020; doi: 10.1016/j.biopha.2020.109985.

54. Varnavides G, Madern M, Anrather D, Hartl N, Reiter W, Hartl M. In search of a universal method: a comparative survey of bottom-up proteomics sample preparation methods. *Journal of proteome research*. 2022; doi: 10.1021/acs.jproteome.2c00265

55. Tabb DL, Vega-Montoto L, Rudnick PA, Variyath AM, Ham AJ, Bunk DM, et al.. Repeatability and reproducibility in proteomic identifications by liquid chromatography− tandem mass spectrometry. *Journal of proteome research*. 2010; doi: 10.1021/pr9006365

56. Perez-Riverol Y, Bai J, Bandla C, García-Seisdedos D, Hewapathirana S, Kamatchinathan S, et al.. The PRIDE database resources in 2022: a hub for mass spectrometry-based proteomics evidences. *Nucleic Acids Res*. 2022; doi: 10.1093/nar/gkab1038.

57. Lobov A, Kuchur P, Boyarskaya N, Perepletchikova D, Taraskin I, Ivashkin A, et al.. Supporting data for "Similar, but not the same: multi-omics comparison of human valve interstitial cells and osteoblast osteogenic differentiation expanded with an estimation of data-dependent and data-independent PASEF proteomic". GigaScience Database. 2024. https://doi.org/10.5524/102619

Click here to access/download;Figure;Fig_1.jpeg

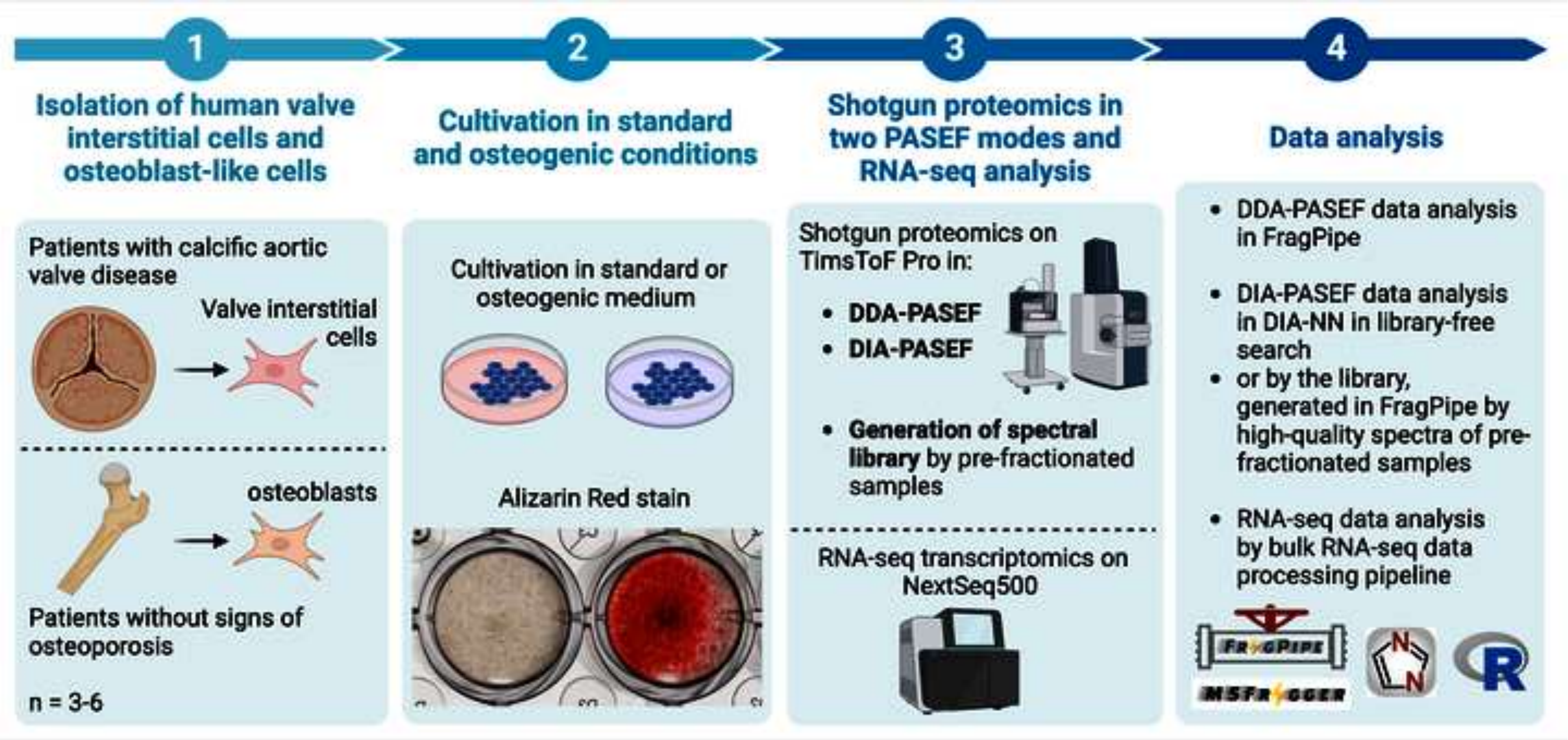

Click here to access/download;Figure;Fig_2_corrected.tiff

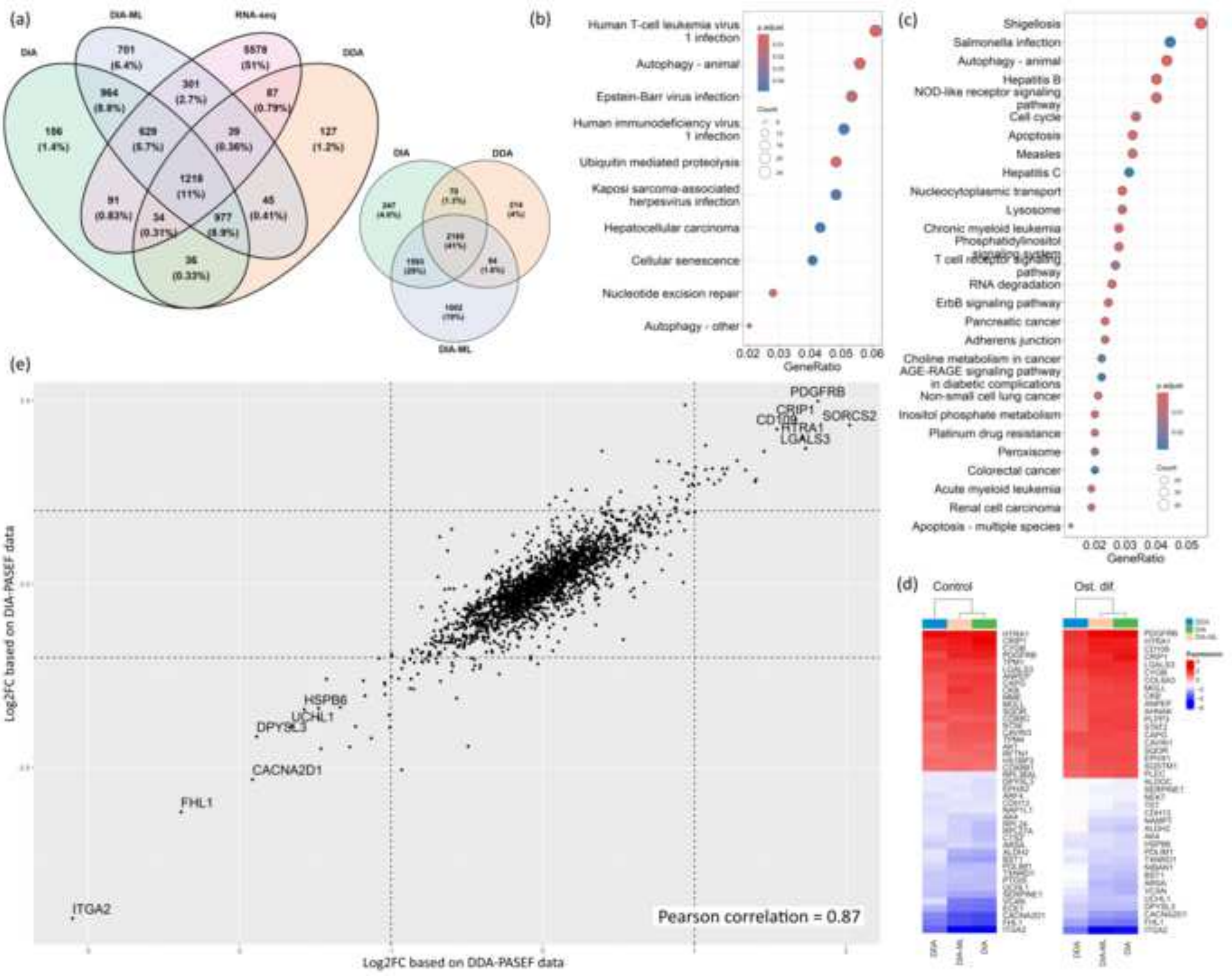

Click here to access/download;Figure;Fig_2.png

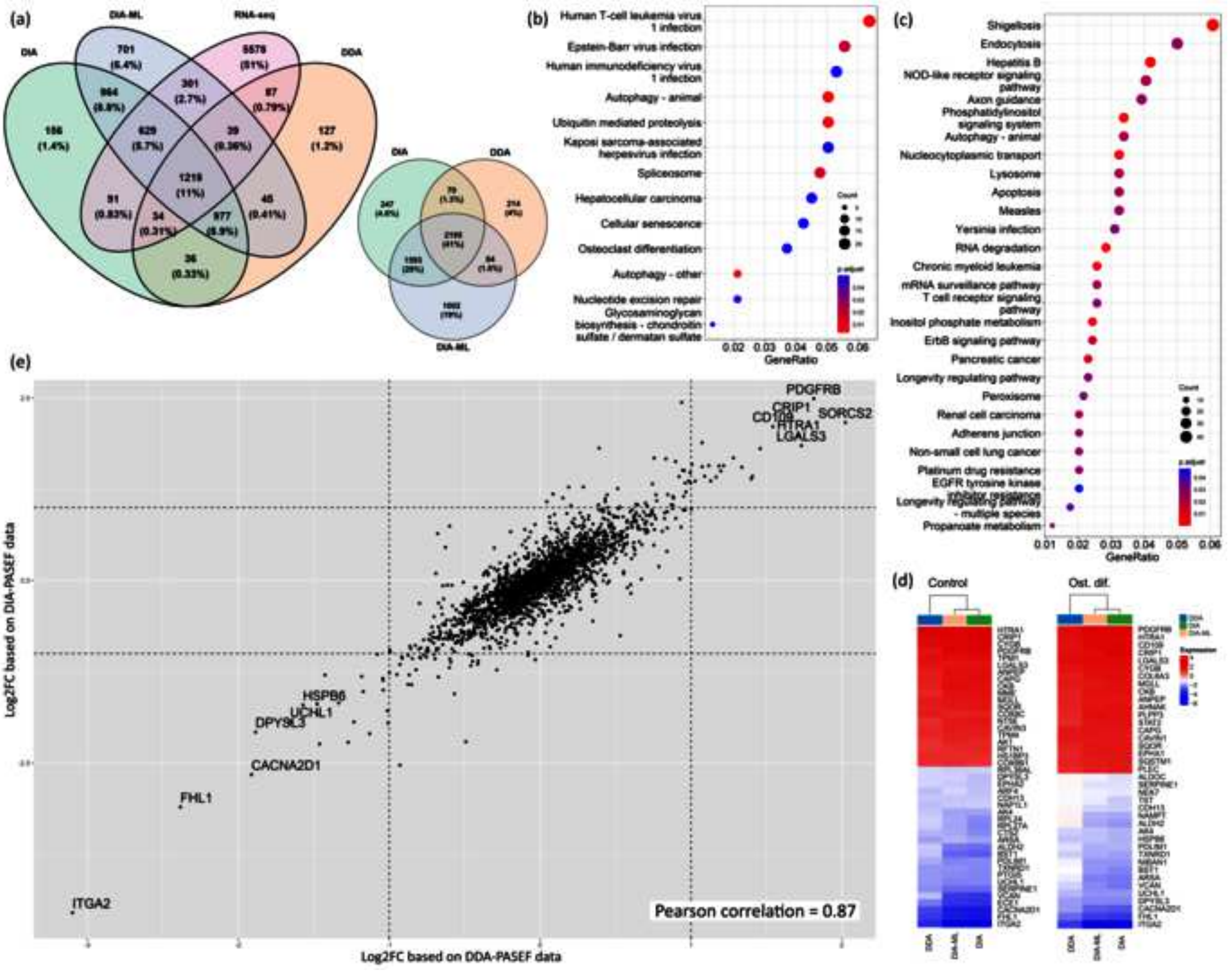

Click here to access/download;Figure;fig3.tiff

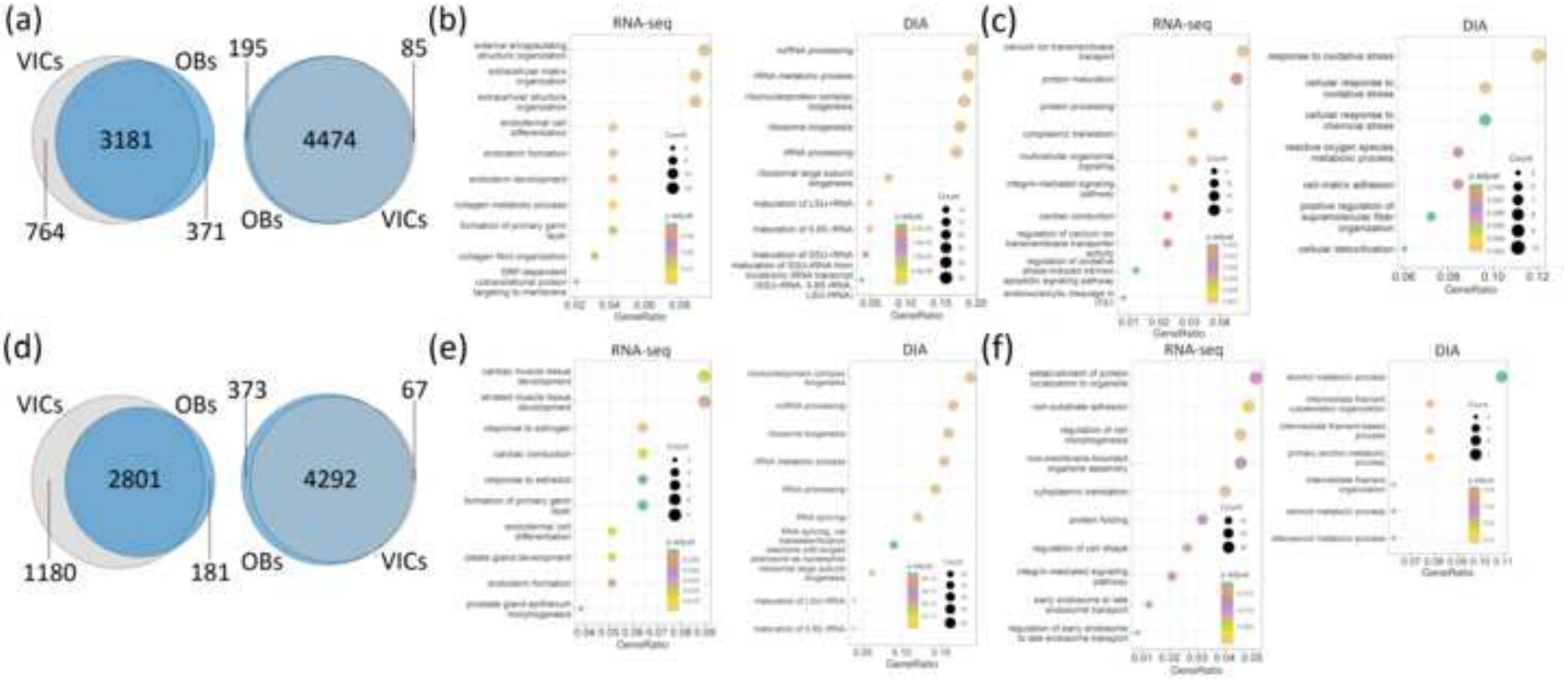

Click here to access/download;Figure;Fig_4.png

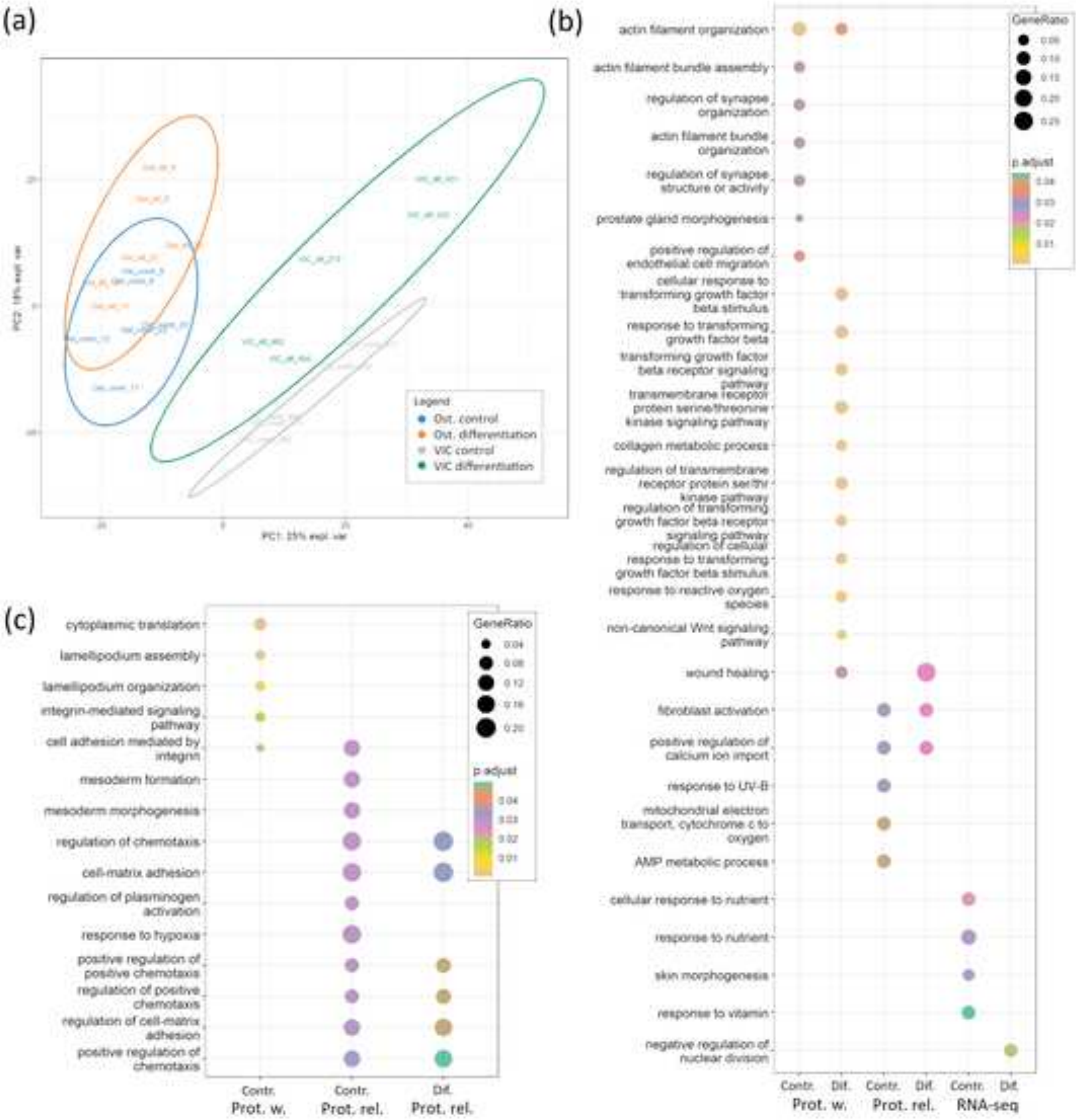

Click here to access/download
Supplementary Material
Supplementary_2-6.docx

Click here to access/download
Supplementary Material
Supplementary_2-5_corrected.docx

Click here to access/download
Supplementary Material
Supplementary_1.xlsx

Click here to access/download
Supplementary Material
Supplementary_3.xlsx

Answers to Reviewers

Click here to access/download;Personal Cover;Revewer answers GiGa.docx

We thank the reviewers for their appreciation and thorough evaluation of our study. All the comments have been addressed as detailed below in point-by-point answers. The changes made appear in orange in the manuscript.

Response to Reviewer #1

Summary of the paper. Here, Lobov and colleagues performed a multimodal comparison of osteogenic signatures in valve interstitial cells and osteoblasts. The authors have combined transcriptomic and proteomic techniques and distinct approaches to the data analysis (data-dependent acquisition, data-independent acquisition with classic library-based search, and data-independent acquisition with machine learning-based library-free search). The authors have identified 2,195 proteins common for all data acquisition methods and 5,405 proteins in total (the number significantly exceeding those could be obtained by any of the techniques), and found data-independent acquisition as the most powerful approach (4,105 proteins) for these datasets. Despite aforementioned differences in proteome coverage, these methods showed an excellent correlation when quantifying the protein expression (0.83 - 0.98) that advocates using each of them but still highlights data-independent acquisition as a technique of choice because of the highest proteome coverage. RNA-seq obtained transcripts for around 8,000 genes but transcriptomic and proteomic data did not correlate well. This is a commonly observed phenomenon in multi-omics studies. In vivo, this might be explained by a high proportion of proteins precipitating from systemic circulation rather than being synthesised by cell populations in situ. Here, the authors profiled the RNA and total protein lysate from the cell cultures, suggesting temporal shifts in transcription and translation of the same molecules.

The combination of distinct techniques and data analysis modalities is a significant advantage of the paper as it improves the methodological basis for analysing multi-omics data and raises the question on optimising the workflow of bioinformatics analysis in relation to the proteomic data.

The authors showed clear clusterisation of undifferentiated (i.e., primary) and differentiated valvular interstitial cells and identified a set of osteogenic proteins charasteristic of VIC-derived osteoblasts. Notably, the molecular pattern of osteogenic differentiation in valvular interstitial cells demonstrated slight differences in comparison with osteoblasts.

General comment

Overall, the study has no glaring omission and deserves to be published, although I would like to recommend extending Discussion by commenting the following issues:...

Authors' response: We sincerely thank the reviewer for appreciating the value of this work and extending the Discussion according to the comments below.

Comment 1.

The reasons behind such differences between transcriptomic and proteomic data?

Authors' response:

Thank you for the suggestion to discuss this point in more detail. We agreed that it is indeed an important and interesting question. We added a short paragraph to discuss this:

"The inherent discrepancies between RNA-seq and proteomics data are well known. It is generally assumed that this phenomenon comes from the variation in translation and protein degradation rates.[35,36] While the exact reason for the poor correlation of mRNA/protein abundance is hard to define, much phenomenological data has been accumulated. Notably, Gygi et al. (1999) represent one of the classic works on yeasts regarding this problem. They showed that the correlation between mRNA and protein abundances cannot predict protein expression. Moreover, the mRNA levels were stable for some genes, but the protein abundances varied more than 20-fold and vice versa.[37] No wonder that in human cells and tissues, we also observe a poor correlation between protein and mRNA abundances, which might be clearly seen in the deep proteome and transcriptome atlas of healthy human tissues.[38] Hundreds of proteins could not be detected even for highly expressed mRNAs. At the same time, protein expression is often more stable across tissues than that of transcripts, which correlates well with our observation regarding higher differences in transcriptome composition between VICs and osteoblasts compared to lower proteome variability (Fig. 3). The mRNA/protein correlation might be especially low for specialized and terminally differentiated tissue, e.g. Moritz et al. (2019) revealed poor to moderate mRNA/protein correlation varying from 0.34 to 0.54 in brain.[39] Similarly, our previous study observed a low correlation between RNA-seq and shotgun proteomic data for various mesenchymal cell primary cultures [32] and VICs [27]. Therefore, we expect a limited correlation between transcriptomic and proteomic data here. Still, comparing the RNA-seq data with various proteomics datasets obtained for the same samples might be used to roughly assess various PASEF techniques."

Comment 2.

The "core sets" of proteins characterising the osteogenic differentiation which are: 1) unique or top differentially expressed in bone-derived and VIC-derived osteoblasts; 2) have been either identified by all three proteomic data acquisition approaches or show similar results when analysing transcriptomic and proteomic data. Adding a table might be useful to show a focused summary for the subsequent studies.

Authors' response:

Thank you for raising this question. As suggested, we added the qualitative analysis showing proteins/transcripts unique for osteoblasts and VICs after osteogenic

differentiation. This analysis is represented in figure 3 and supplementary materials 3 in the current version of the manuscript:

“To better characterize the cells studied we started from qualitative analysis (presence/absence) of transcriptome and proteome composition (focusing on DIA dataset as the most representative one). As expected, we observed significant differences in transcriptome composition between VICs and osteoblasts in standard cultivation, while proteome composition was more similar (Fig. 3 a; supplementary materials 3). Transcripts specific for osteoblasts are associated with extracellular matrix and collagen fibril organization. Proteomic data reinforce the importance of pathways such as ribosome biogenesis and RNA splicing (Fig. 3 b). In contrast, for VICs we observed unique signaling and protein processing transcripts (Fig. 3 c). These differences become increased even more after osteogenic differentiation (Fig. 3 d-f).”

Regarding the second suggestion, we would like to emphesize that we underlined proteins being overexpressed by both RNA-seq and proteomics data in Table 2 and focused the discussion on them. We agreed that these proteins are the most interesting ones.

Comment 3.

The correlations between the differentially expressed pathways and abovementioned osteogenic protein signatures of bone-derived and VIC-derived osteoblasts (which proteins do correspond to which pathway). Adding a table might be useful to show a focused summary for the subsequent studies.

Authors’ response:

As suggested, we added the pathway enrichment analysis for the unique proteins mentioned above in Figure 3 (please see also the detailed answer within the answer to comment 2). We also briefly discussed comparing this analysis's results with the pathway enrichment results for differentially expressed proteins:

“Notably, there is a similarity between the gene ontology analysis for cell-type specific (Fig. 3) and differentially expressed (Fig. 4) transcripts/proteins. Particularly, both types of analyses identify genes associated with cytoplasmic translation, integrin-mediated signaling pathway, and regulation of cell matrix adhesion for VICs; while for osteoblasts, the similarity is associated with various extracellular matrix-related terms.”

Response to Reviewer #2

Summary of the paper. This study employed RNA sequencing transcriptomics and proteomics to explore the multi-omics profiles of valve interstitial cells (VICs) and osteoblasts during osteogenic differentiation. The study identified potential therapeutic

targets specific for VICs osteogenic differentiation in CAVD, including MAOA and ERK1/2 pathway. The authors conclude that DIA-based methods demonstrate even higher superiority against DDA for more sophisticated human primary cell cultures than it was shown before on test samples. While the classic library-based DIA approach has proved to be a golden standard for shotgun proteomics research, the DIA-ML offers significant advantages with a relatively minor compromise in data reliability, making it the method of choice for routine proteomics.

General comment

The study addresses and interesting and biologically relevant topic. The comparison of proteomic techniques should be of interest to readers. However, there are questions about the nature of cells being analyzed with the seeming lack of osteoblast specific genes/proteins suggesting either mixed cell lineages or possibly a more terminally differentiated cell population such as osteocytes, How this would affect the interpretation of the data is not clear. A cleaner characterization of the cell populations would strengthen the manuscript.

Authors' response: We sincerely thank the reviewer for the feedback and for recognizing the relevance of our work. We agree that a more detailed characterization of the cell populations might enhance the manuscript. However, the primary focus of our study is on the comparative analysis of proteomic techniques rather than on cellular characterization. The cells we used were comprehensively characterized in several of our recent studies (Kostina et al., 2021, 2022; Semenova et al., 2022; Bogdanova et al., 2019), where their osteoblast-specific markers and differentiation status were well-defined.

To maintain focus on the technical aspects of shotgun proteomics, we opted to limit the scope of the cellular characterization in this paper. Nevertheless, we have carefully addressed the concerns raised by citing our previous papers, where we introduced these cell isolation protocols, and by thoroughly discussing the potential implications of interpreting our data. We believe this approach balances the technical emphasis of our current work and the biological context. Please find more detailed answers below.

Comment 1-2.

The isolation protocol for bone is likely collecting more osteocytes than osteoblasts.

Data figure of OB characterization. Alizarin Red S staining was performed but data not clearly shown. Is this the 2 wells in Fig.1? If so, the manuscript would be significantly improved by including a better characterization of the cells. As ARS binds calcium it is possible that the positive stating is a Calcium-phosphate precipitate and not necessarily hydroxyapatite which is normally formed initially as nodules. Therefore, increased ALP staining or enzyme activity, changes in maker gene/protein expression such as Col-1, BSP, osteocalcin, OSX, Runx2, etc. would make a stronger case that

the cells are actually differentiating to osteoblasts as claimed. Likewise, some characterization of the VICs would also strengthen the conclusions.

Authors' response:

We limited the characterization of the cells in this paper because we comprehensively characterized these cells and their osteogenic differentiation in detail in previous papers (Kostina et al., 2021, 2022; Semenova et al., 2022; Bogdanova et al., 2019), and here, we aimed to focus on the proteomics data analysis rather than on the biology of these cell types. Nevertheless, we agreed that this is an important concern. Therefore, we included additional explanations in the analyses section:

"In this work, we used osteoblast and valve interstitial cells (VICs) isolated by the standard protocol comprehensively characterized by us previously [28,29]. Osteoblasts were isolated from a femur of adult patients and represent the terminally differentiated cells expressing RUNX2, BGLAP, and COL1A1 even in standard cultivation as was shown before. Still, an osteogenic medium is necessary for extracellular matrix mineralization [28]. VICs were isolated from calcified aortic valve leaflets of adult patients, which makes them similar to osteoblasts - both cells were isolated from calcified tissues. Still, VICs do not express osteogenic markers and do not show ALP activity in standard cultivation [29,30]."

Comment 3.

A Table of OB or VIC specific gene/protein expression, even if not significantly altered might provide additional information as to the characterization of the cells.

Authors' response:

Thank you for this suggestion. We included the qualitative analysis to characterize the gene/protein expression profiles of osteoblasts and VICs. The analysis is presented now as figure 3 with the description as follow:

"To better characterize the cells studied we started from qualitative analysis (presence/absence) of transcriptome and proteome (focusing on DIA dataset as the most representative one) composition. As expected, we observed significant differences in transcriptome composition between VICs and osteoblasts in standard cultivation, while proteome composition was more similar (Fig. 3 a). Transcripts specific for osteoblasts are associated with extracellular matrix and collagen fibril organization. Proteomic data reinforce the importance of pathways such as ribosome biogenesis and RNA splicing (Fig. 3 b). In contrast, for VICs we observed unique signaling and protein processing transcripts (Fig. 3 c). These differences become increase even more after osteogenic differentiation (Fig. 3 d-f)."

Comment 4.

Osteoclast genes are noted with the osteoblast differentiation dataset however these are very different cell types expressing a very different gene profile so the result might suggest contamination with osteoclasts and mixed lineages of cell populations?

Authors' response:

This is a very important observation; indeed, the osteoblasts express some of the proteins traditionally regarded as specific for osteoclasts. The recent development in the single-cell RNA-seq data allows us to uncover natural cell heterogeneity. It shows that despite the significant differences between various cell types (particularly between osteoblasts and osteoclasts), the number of qualitative differences (presence/absence of protein expression) is limited (Lee et al., 2023). Specifically, by the data of human bone marrow scRNA-seq atlas, many osteoclast-related proteins can be found in osteoblasts (https://www.immunesinglecell.org/atlas/bone_marrow; Lee et al., 2023). MMP9, ACP5 and CTSK expression are regarded as specific markers of osteoclasts. Indeed, the MMP9 and ACP5 are not expressed in our samples, which clearly shows that we do not have osteoclast contamination.

Moreover, osteoclasts are myeloid-origin cells expressing specific surface antigens (CD34 and CD117). Previously, we described in detail the protocol for osteoblasts isolation used here and showed the absence of these markers by flow cytometry (Kostina et al., 2022). So, the surface antigen markers for osteoblasts used in this work are different for osteoclasts.

Finally, we emphasize that the expression of some osteoclast-related genes in osteoblasts is well-known in the literature. Moreover, the expression of many of them in the osteoblasts is vital for osteogenesis. In particular, we would like to underline TAB2, PIK3CB, PIK3R2, and CTSK, which were found to be expressed in osteoblasts in our study. Kim et al. (2018), showed the deregulation of NF-κB by TAB2 degradation in osteoblasts and osteoclasts types (Kim et al., 2018). Similarly, PI-3-kinase class I plays a central role in bone formation through SMAD1 regulation in osteoblasts (Gámez et al., 2016). CTSK is traditionally regarded as an osteoclast protease, but osteoblasts are known to secrete it and also contribute to extracellular matrix remodeling (Mandelin et al., 2006).

We also added clarification regarding this in the Analyses section:

"Still, we do not observed expression of MMP9 and ACP5, identified to be specific markers of osteoclast linage by scRNA-seq human bone marrow atlas (immunesinglecell.org/atlas/bone_marrow; accessed 11/10/24)[28] and, as we showed before, we have not found the presence of osteoclast-associated surface antigens (CD34 and CD117) in the osteoblast cells used here.[29]"

Comment 5.

The fact that the cells were cultured instead of taken straight from the patient might also confound interpretation as this likely significantly alters gene and protein expression.

Authors' response:

We absolutely agree that the best way for our study would be to take the cells directly from the patient. Nevertheless, it is technically impossible due to the limitations for sample amounts in shotgun proteomics. This is especially true for our study in which we used the same cells for RNA-seq, protein fractionation for spectral library preparation, and two runs of shotgun proteomics with various data acquisition modes. Even with the high sensitivity of the TimsToF platform, the amount of protein that might be isolated straight from the patient would not be enough.

To make these limitations also clear for the readers, we added the "Limitations of the study" section at the end of the discussion:

"Finally, we would like to emphasize the limitations of the study. First, we cultured primary cell cultures for several passages in vitro due to the high amount of samples needed. For this, we used a well-defined methodology [9, 27,29,30,33], guaranteeing that we worked with well-defined cell types but also limited our observation relative to the native tissue. Still, as discussed above, many of our observations correlate well with the data from the whole-tissue models. Other significant limitations are associated with general poor proteomics reproducibility and rapid development of data analysis tools. Particularly, Varnavides et al. (2022) compared 16 shotgun proteomics sample preparation methods and revealed method-dependent differences in specific protein features identification.[54] Beyond sample preparation, the data-dependent acquisition approach used by us for the DDA dataset and spectral library preparation is also known to have limited reproducibility due to stochastic sampling.[55] Finally, the software used in this study was significantly improved even during the time between submitting and publishing the presented manuscript (especially in the case of library-free DIA workflow)."
