# Supplementary material for: Similar, but not the same: multiomics comparison of human valve interstitial cells and osteoblast osteogenic differentiation expanded with an estimation of data-dependent and data-independent PASEF proteomics
Source: Gigascience. 2025 Jan 11;14:giae110. doi: 10.1093/gigascience/giae110 (PMC11724719; doi:10.1093/gigascience/giae110)
Supplement: giae110_Response_to_Reviewer_Comments_Original_Submission [file giae110_response_to_reviewer_comments_original_submission.pdf]

Author's Response To Reviewer Comments

We thank the reviewers for their appreciation and thorough evaluation of our study. All the comments have been addressed as detailed below in point-by-point answers. The changes made appear in orange in the manuscript.

Response to Reviewer #1
Summary of the paper. Here, Lobov and colleagues performed a multimodal comparison of osteogenic signatures in valve interstitial cells and osteoblasts. The authors have combined transcriptomic and proteomic techniques and distinct approaches to the data analysis (data-dependent acquisition, data-independent acquisition with classic library-based search, and data-independent acquisition with machine learning-based library-free search). The authors have identified 2,195 proteins common for all data acquisition methods and 5,405 proteins in total (the number significantly exceeding those could be obtained by any of the techniques), and found data-independent acquisition as the most powerful approach (4,105 proteins) for these datasets. Despite aforementioned differences in proteome coverage, these methods showed an excellent correlation when quantifying the protein expression (0.83 - 0.98) that advocates using each of them but still highlights data-independent acquisition as a technique of choice because of the highest proteome coverage. RNA-seq obtained transcripts for around 8,000 genes but transcriptomic and proteomic data did not correlate well. This is a commonly observed phenomenon in multi-omics studies. In vivo, this might be explained by a high proportion of proteins precipitating from systemic circulation rather than being synthesised by cell populations in situ. Here, the authors profiled the RNA and total protein lysate from the cell cultures, suggesting temporal shifts in transcription and translation of the same molecules.
The combination of distinct techniques and data analysis modalities is a significant advantage of the paper as it improves the methodological basis for analysing multi-omics data and raises the question on optimising the workflow of bioinformatics analysis in relation to the proteomic data.
The authors showed clear clusterisation of undifferentiated (i.e., primary) and differentiated valvular interstitial cells and identified a set of osteogenic proteins charasteristic of VIC-derived osteoblasts. Notably, the molecular pattern of osteogenic differentiation in valvular interstitial cells demonstrated slight differences in comparison with osteoblasts.
General comment
Overall, the study has no glaring omission and deserves to be published, although I would like to recommend extending Discussion by commenting the following issues:...
Authors' response: We sincerely thank the reviewer for appreciating the value of this work and extending the Discussion according to the comments below.
Comment 1.
The reasons behind such differences between transcriptomic and proteomic data?
Authors' response:
Thank you for the suggestion to discuss this point in more detail. We agreed that it is indeed an important and interesting question. We added a short paragraph to discuss this:
"The inherent discrepancies between RNA-seq and proteomics data are well known. It is generally assumed that this phenomenon comes from the variation in translation and protein degradation rates.[35,36] While the exact reason for the poor correlation of mRNA/protein abundance is hard to define, much phenomenological data has been accumulated. Notably, Gygi et al. (1999) represent one of the classic works on yeasts regarding this problem. They showed that the correlation between mRNA and protein abundances cannot predict protein expression. Moreover, the mRNA levels were stable for some genes, but the protein abundances varied more than 20-fold and vice versa.[37] No wonder that in human cells and tissues, we also observe a poor correlation between protein and mRNA abundances, which might be clearly seen in the deep proteome and transcriptome atlas of healthy human tissues.[38] Hundreds of proteins could not be detected even for highly expressed mRNAs. At the same time, protein expression is often more stable across tissues than that of transcripts, which correlates well with our observation regarding higher differences in transcriptome composition between VICs and osteoblasts compared to lower proteome variability (Fig. 3). The mRNA/protein correlation might be especially low for specialized and terminally differentiated tissue, e.g. Moritz et al. (2019) revealed poor to moderate mRNA/protein correlation varying from 0.34 to 0.54 in brain.[39] Similarly, our previous study observed a low correlation between RNA-seq

and shotgun proteomic data for various mesenchymal cell primary cultures [32] and VICs [27]. Therefore, we expect a limited correlation between transcriptomic and proteomic data here. Still, comparing the RNA-seq data with various proteomics datasets obtained for the same samples might be used to roughly assess various PASEF techniques.‟

Comment 2.
The "core sets" of proteins characterising the osteogenic differentiation which are: 1) unique or top differentially expressed in bone-derived and VIC-derived osteoblasts; 2) have been either identified by all three proteomic data acquisition approaches or show similar results when analysing transcriptomic and proteomic data. Adding a table might be useful to show a focused summary for the subsequent studies.
Authors’ response:
Thank you for raising this question. As suggested, we added the qualitative analysis showing proteins/transcripts unique for osteoblasts and VICs after osteogenic differentiation. This analysis is represented in figure 3 and supplementary materials 3 in the current version of the manuscript:
‟To better characterize the cells studied we started from qualitative analysis (presence/absence) of transcriptome and proteome composition (focusing on DIA dataset as the most representative one). As expected, we observed significant differences in transcriptome composition between VICs and osteoblasts in standard cultivation, while proteome composition was more similar (Fig. 3 a; supplementary materials 3). Transcripts specific for osteoblasts are associated with extracellular matrix and collagen fibril organization. Proteomic data reinforce the importance of pathways such as ribosome biogenesis and RNA splicing (Fig. 3 b). In contrast, for VICs we observed unique signaling and protein processing transcripts (Fig. 3 c). These differences become increased even more after osteogenic differentiation (Fig. 3 d-f).”
Regarding the second suggestion, we would like to emphesize that we underlined proteins being overexpressed by both RNA-seq and proteomics data in Table 2 and focused the discussion on them. We agreed that these proteins are the most interesting ones.

Comment 3.
The correlations between the differentially expressed pathways and abovementioned osteogenic protein signatures of bone-derived and VIC-derived osteoblasts (which proteins do correspond to which pathway). Adding a table might be useful to show a focused summary for the subsequent studies.
Authors’ response:
As suggested, we added the pathway enrichment analysis for the unique proteins mentioned above in Figure 3 (please see also the detailed answer within the answer to comment 2). We also briefly discussed comparing this analysis's results with the pathway enrichment results for differentially expressed proteins:
‟Notably, there is a similarity between the gene ontology analysis for cell-type specific (Fig. 3) and differentially expressed (Fig. 4) transcripts/proteins. Particularly, both types of analyses identify genes associated with cytoplasmic translation, integrin-mediated signaling pathway, and regulation of cell matrix adhesion for VICs; while for osteoblasts, the similarity is associated with various extracellular matrix-related terms.”
Response to Reviewer #2
Summary of the paper. This study employed RNA sequencing transcriptomics and proteomics to explore the multi-omics profiles of valve interstitial cells (VICs) and osteoblasts during osteogenic differentiation. The study identified potential therapeutic targets specific for VICs osteogenic differentiation in CAVD, including MAOA and ERK1/2 pathway. The authors conclude that DIA-based methods demonstrate even higher superiority against DDA for more sophisticated human primary cell cultures than it was shown before on test samples. While the classic library-based DIA approach has proved to be a golden standard for shotgun proteomics research, the DIA-ML offers significant advantages with a relatively minor compromise in data reliability, making it the method of choice for routine proteomics.
General comment
The study addresses and interesting and biologically relevant topic. The comparison of proteomic techniques should be of interest to readers. However, there are questions about the nature of cells being analyzed with the seeming lack of osteoblast specific genes/proteins suggesting either mixed cell lineages or possibly a more terminally differentiated cell population such as osteocytes, How this would affect the interpretation of the data is not clear. A cleaner characterization of the cell populations would strengthen the manuscript.
Authors’ response: We sincerely thank the reviewer for the feedback and for recognizing the relevance of our work. We agree that a more detailed characterization of the cell populations might enhance the

manuscript. However, the primary focus of our study is on the comparative analysis of proteomic techniques rather than on cellular characterization. The cells we used were comprehensively characterized in several of our recent studies (Kostina et al., 2021, 2022; Semenova et al., 2022; Bogdanova et al., 2019), where their osteoblast-specific markers and differentiation status were well-defined.
To maintain focus on the technical aspects of shotgun proteomics, we opted to limit the scope of the cellular characterization in this paper. Nevertheless, we have carefully addressed the concerns raised by citing our previous papers, where we introduced these cell isolation protocols, and by thoroughly discussing the potential implications of interpreting our data. We believe this approach balances the technical emphasis of our current work and the biological context. Please find more detailed answers below.
Comment 1-2.
The isolation protocol for bone is likely collecting more osteocytes than osteoblasts.
Data figure of OB characterization. Alizarin Red S staining was performed but data not clearly shown. Is this the 2 wells in Fig.1? If so, the manuscript would be significantly improved by including a better characterization of the cells. As ARS binds calcium it is possible that the positive stating is a Calcium-phosphate precipitate and not necessarily hydroxyapatite which is normally formed initially as nodules. Therefore, increased ALP staining or enzyme activity, changes in maker gene/protein expression such as Col-1, BSP, osteocalcin, OSX, Runx2, etc. would make a stronger case that the cells are actually differentiating to osteoblasts as claimed. Likewise, some characterization of the VICs would also strengthen the conclusions.
Authors' response:
We limited the characterization of the cells in this paper because we comprehensively characterized these cells and their osteogenic differentiation in detail in previous papers (Kostina et al., 2021, 2022; Semenova et al., 2022; Bogdanova et al., 2019), and here, we aimed to focus on the proteomics data analysis rather than on the biology of these cell types. Nevertheless, we agreed that this is an important concern. Therefore, we included additional explanations in the analyses section:
"In this work, we used osteoblast and valve interstitial cells (VICs) isolated by the standard protocol comprehensively characterized by us previously [28,29]. Osteoblasts were isolated from a femur of adult patients and represent the terminally differentiated cells expressing RUNX2, BGLAP, and COL1A1 even in standard cultivation as was shown before. Still, an osteogenic medium is necessary for extracellular matrix mineralization [28]. VICs were isolated from calcified aortic valve leaflets of adult patients, which makes them similar to osteoblasts - both cells were isolated from calcified tissues. Still, VICs do not express osteogenic markers and do not show ALP activity in standard cultivation [29,30]."

Comment 3.
A Table of OB or VIC specific gene/protein expression, even if not significantly altered might provide additional information as to the characterization of the cells.
Authors' response:
Thank you for this suggestion. We included the qualitative analysis to characterize the gene/protein expression profiles of osteoblasts and VICs. The analysis is presented now as figure 3 with the description as follow:
"To better characterize the cells studied we started from qualitative analysis (presence/absence) of transcriptome and proteome (focusing on DIA dataset as the most representative one) composition. As expected, we observed significant differences in transcriptome composition between VICs and osteoblasts in standard cultivation, while proteome composition was more similar (Fig. 3 a). Transcripts specific for osteoblasts are associated with extracellular matrix and collagen fibril organization. Proteomic data reinforce the importance of pathways such as ribosome biogenesis and RNA splicing (Fig. 3 b). In contrast, for VICs we observed unique signaling and protein processing transcripts (Fig. 3 c). These differences become increase even more after osteogenic differentiation (Fig. 3 d-f)."

Comment 4.
Osteoclast genes are noted with the osteoblast differentiation dataset however these are very different cell types expressing a very different gene profile so the result might suggest contamination with osteoclasts and mixed lineages of cell populations?
Authors' response:
This is a very important observation; indeed, the osteoblasts express some of the proteins traditionally regarded as specific for osteoclasts. The recent development in the single-cell RNA-seq data allows us to uncover natural cell heterogeneity. It shows that despite the significant differences between various cell

types (particularly between osteoblasts and osteoclasts), the number of qualitative differences (presence/absence of protein expression) is limited (Lee et al., 2023). Specifically, by the data of human bone marrow scRNA-seq atlas, many osteoclast-related proteins can be found in osteoblasts (https://www.immunesinglecell.org/atlas/bone_marrow; Lee et al., 2023). MMP9, ACP5 and CTSK expression are regarded as specific markers of osteoclasts. Indeed, the MMP9 and ACP5 are not expressed in our samples, which clearly shows that we do not have osteoclast contamination.
Moreover, osteoclasts are myeloid-origin cells expressing specific surface antigens (CD34 and CD117). Previously, we described in detail the protocol for osteoblasts isolation used here and showed the absence of these markers by flow cytometry (Kostina et al., 2022). So, the surface antigen markers for osteoblasts used in this work are different for osteoclasts.
Finally, we emphasize that the expression of some osteoclast-related genes in osteoblasts is well-known in the literature. Moreover, the expression of many of them in the osteoblasts is vital for osteogenesis. In particular, we would like to underline TAB2, PIK3CB, PIK3R2, and CTSK, which were found to be expressed in osteoblasts in our study. Kim et al. (2018), showed the deregulation of NF-κB by TAB2 degradation in osteoblasts and osteoclasts types (Kim et al., 2018). Similarly, PI-3-kinase class I plays a central role in bone formation through SMAD1 regulation in osteoblasts (Gámez et al., 2016). CTSK is traditionally regarded as an osteoclast protease, but osteoblasts are known to secrete it and also contribute to extracellular matrix remodeling (Mandelin et al., 2006).
We also added clarification regarding this in the Analyses section:
"Still, we do not observed expression of MMP9 and ACP5, identified to be specific markers of osteoclast linage by scRNA-seq human bone marrow atlas (immunesinglecell.org/atlas/bone_marrow; accessed 11/10/24)[28] and, as we showed before, we have not found the presence of osteoclast-associated surface antigens (CD34 and CD117) in the osteoblast cells used here.[29]"
Comment 5.
The fact that the cells were cultured instead of taken straight from the patient might also confound interpretation as this likely significantly alters gene and protein expression.
Authors' response:
We absolutely agree that the best way for our study would be to take the cells directly from the patient. Nevertheless, it is technically impossible due to the limitations for sample amounts in shotgun proteomics. This is especially true for our study in which we used the same cells for RNA-seq, protein fractionation for spectral library preparation, and two runs of shotgun proteomics with various data acquisition modes. Even with the high sensitivity of the TimsToF platform, the amount of protein that might be isolated straight from the patient would not be enough.
To make these limitations also clear for the readers, we added the "Limitations of the study" section at the end of the discussion:
"Finally, we would like to emphasize the limitations of the study. First, we cultured primary cell cultures for several passages in vitro due to the high amount of samples needed. For this, we used a well-defined methodology [9, 27,29,30,33], guaranteeing that we worked with well-defined cell types but also limited our observation relative to the native tissue. Still, as discussed above, many of our observations correlate well with the data from the whole-tissue models. Other significant limitations are associated with general poor proteomics reproducibility and rapid development of data analysis tools. Particularly, Varnavides et al. (2022) compared 16 shotgun proteomics sample preparation methods and revealed method-dependent differences in specific protein features identification.[54] Beyond sample preparation, the data-dependent acquisition approach used by us for the DDA dataset and spectral library preparation is also known to have limited reproducibility due to stochastic sampling.[55] Finally, the software used in this study was significantly improved even during the time between submitting and publishing the presented manuscript (especially in the case of library-free DIA workflow)."
