# Supplementary material for: Similar, but not the same: multiomics comparison of human valve interstitial cells and osteoblast osteogenic differentiation expanded with an estimation of data-dependent and data-independent PASEF proteomics
Source: Gigascience. 2025 Jan 11;14:giae110. doi: 10.1093/gigascience/giae110 (PMC11724719; doi:10.1093/gigascience/giae110)
Supplement: giae110_Reviewer_1_Report_Original_Submission — Anton Kutikhin -- 5/31/2024 Reviewed [file giae110_reviewer_1_report_original_submission.pdf]

Reviewer Report

Title: Similar, but not the same: multi-omics comparison of human valve interstitial cells and osteoblast osteogenic differentiation expanded with an estimation of data-dependent and data-independent PASEF proteomics

Version: Original Submission **Date:** 5/31/2024

Reviewer name: Anton Kutikhin

Reviewer Comments to Author:

Here, Lobov and colleagues performed a multimodal comparison of osteogenic signatures in valve interstitial cells and osteoblasts. The authors have combined transcriptomic and proteomic techniques and distinct approaches to the data analysis (data-dependent acquisition, data-independent acquisition with classic library-based search, and data-independent acquisition with machine learning-based library-free search). The authors have identified 2,195 proteins common for all data acquisition methods and 5,405 proteins in total (the number significantly exceeding those could be obtained by any of the techniques), and found data-independent acquisition as the most powerful approach (4,105 proteins) for these datasets. Despite aforementioned differences in proteome coverage, these methods showed an excellent correlation when quantifying the protein expression (0.83 - 0.98) that advocates using each of them but still highlights data-independent acquisition as a technique of choice because of the highest proteome coverage.
RNA-seq obtained transcripts for around 8,000 genes but transcriptomic and proteomic data did not correlate well. This is a commonly observed phenomenon in multi-omics studies. In vivo, this might be explained by a high proportion of proteins precipitating from systemic circulation rather than being synthesised by cell populations in situ. Here, the authors profiled the RNA and total protein lysate from the cell cultures, suggesting temporal shifts in transcription and translation of the same molecules.
The combination of distinct techniques and data analysis modalities is a significant advantage of the paper as it improves the methodological basis for analysing multi-omics data and raises the question on optimising the workflow of bioinformatics analysis in relation to the proteomic data.
The authors showed clear clusterisation of undifferentiated (i.e., primary) and differentiated valvular interstitial cells and identified a set of osteogenic proteins charasteristic of VIC-derived osteoblasts. Notably, the molecular pattern of osteogenic differentiation in valvular interstitial cells demonstrated slight differences in comparison with osteoblasts.
Overall, the study has no glaring omission and deserves to be published, although I would like to recommend extending Discussion by commenting the following issues:
1. The reasons behind such differences between transcriptomic and proteomic data?
2. The "core sets" of proteins characterising the osteogenic differentiation which are: 1) unique or top differentially expressed in bone-derived and VIC-derived osteoblasts; 2) have been either identified by all three proteomic data acquisition approaches or show similar results when analysing transcriptomic and proteomic data. Adding a table might be useful to show a focused summary for the subsequent studies.

3. The correlations between the differentially expressed pathways and abovementioned osteogenic protein signatures of bone-derived and VIC-derived osteoblasts (which proteins do correspond to which pathway). Adding a table might be useful to show a focused summary for the subsequent studies.

Methods

Are the methods appropriate to the aims of the study, are they well described, and are necessary controls included? Choose an item.

Conclusions

Are the conclusions adequately supported by the data shown? Choose an item.

Reporting Standards

Does the manuscript adhere to the journal's guidelines on minimum standards of reporting? Choose an item.

Choose an item.

Statistics

Are you able to assess all statistics in the manuscript, including the appropriateness of statistical tests used? Choose an item.

Quality of Written English

Please indicate the quality of language in the manuscript: Choose an item.

Declaration of Competing Interests

Please complete a declaration of competing interests, considering the following questions:

- Have you in the past five years received reimbursements, fees, funding, or salary from an organisation that may in any way gain or lose financially from the publication of this manuscript, either now or in the future?
- Do you hold any stocks or shares in an organisation that may in any way gain or lose financially from the publication of this manuscript, either now or in the future?
- Do you hold or are you currently applying for any patents relating to the content of the manuscript?
- Have you received reimbursements, fees, funding, or salary from an organization that holds or has applied for patents relating to the content of the manuscript?
- Do you have any other financial competing interests?
- Do you have any non-financial competing interests in relation to this paper?

If you can answer no to all of the above, write 'I declare that I have no competing interests' below. If your reply is yes to any, please give details below.

I declare that I have no competing interests.

I agree to the open peer review policy of the journal. I understand that my name will be included on my report to the authors and, if the manuscript is accepted for publication, my named report including any attachments I upload will be posted on the website along with the authors' responses. I agree for my report to be made available under an Open Access Creative Commons CC-BY license (http://creativecommons.org/licenses/by/4.0/). I understand that any comments which I do not wish to be included in my named report can be included as confidential comments to the editors, which will not be published.

Choose an item.

To further support our reviewers, we have joined with Publons, where you can gain additional credit to further highlight your hard work (see: https://publons.com/journal/530/gigascience). On publication of this paper, your review will be automatically added to Publons, you can then choose whether or not to claim your Publons credit. I understand this statement.

Yes Choose an item.
