# Supplementary material for: Similar, but not the same: multiomics comparison of human valve interstitial cells and osteoblast osteogenic differentiation expanded with an estimation of data-dependent and data-independent PASEF proteomics
Source: Gigascience. 2025 Jan 11;14:giae110. doi: 10.1093/gigascience/giae110 (PMC11724719; doi:10.1093/gigascience/giae110)
Supplement: giae110_Reviewer_2_Report_Original_Submission — George R Beck -- 9/23/2024 Reviewed [file giae110_reviewer_2_report_original_submission.pdf]

Reviewer Report

Title: Similar, but not the same: multi-omics comparison of human valve interstitial cells and osteoblast osteogenic differentiation expanded with an estimation of data-dependent and data-independent PASEF proteomics

Version: Original Submission **Date:** 9/23/2024

Reviewer name: George R Beck

Reviewer Comments to Author:

Manuscript Number: GIGA-D-24-00130
Full Title: Similar, but not the same: multi-omics comparison of human valve interstitial cells and osteoblast osteogenic differentiation expanded with an estimation of data-dependent and data-independent PASEF proteomic.
Summary: This study employed RNA sequencing transcriptomics and proteomics to explore the multi-omics profiles of valve interstitial cells (VICs) and osteoblasts during osteogenic differentiation. The study identified potential therapeutic targets specific for VICs osteogenic differentiation in CAVD, including MAOA and ERK1/2 pathway. The authors conclude that DIA-based methods demonstrate even higher superiority against DDA for more sophisticated human primary cell cultures than it was shown before on test samples. While the classic library-based DIA approach has proved to be a golden standard for shotgun proteomics research, the DIA-ML offers significant advantages with a relatively minor compromise in data reliability, making it the method of choice for routine proteomics.
Comments: The study addresses and interesting and biologically relevant topic. The comparison of proteomic techniques should be of interest to readers. However, there are questions about the nature of cells being analyzed with the seeming lack of osteoblast specific genes/proteins suggesting either mixed cell lineages or possibly a more terminally differentiated cell population such as osteocytes, How this would affect the interpretation of the data is not clear. A cleaner characterization of the cell populations would strengthen the manuscript.
1) The isolation protocol for bone is likely collecting more osteocytes than osteoblasts.
2) Data figure of OB characterization. Alizarin Red S staining was performed but data not clearly shown. Is this the 2 wells in Fig.1? If so, the manuscript would be significantly improved by including a better characterization of the cells. As ARS binds calcium it is possible that the positive stating is a Calcium-phosphate precipitate and not necessarily hydroxyapatite which is normally formed initially as nodules. Therefore, increased ALP staining or enzyme activity, changes in maker gene/protein expression such as Col-1, BSP, osteocalcin, OSX, Runx2, etc. would make a stronger case that the cells are actually differentiating to osteoblasts as claimed. Likewise, some characterization of the VICs would also strengthen the conclusions.
3) A Table of OB or VIC specific gene/protein expression, even if not significantly altered might provide additional information as to the characterization of the cells.
4) Osteoclast genes are noted with the osteoblast differentiation dataset however these are very different cell types expressing a very different gene profile so the result might suggest contamination

with osteoclasts and mixed lineages of cell populations?
5) The fact that the cells were cultured instead of taken straight from the patient might also confound interpretation as this likely significantly alters gene and protein expression.

Methods

Are the methods appropriate to the aims of the study, are they well described, and are necessary controls included? Choose an item.

Conclusions

Are the conclusions adequately supported by the data shown? Choose an item.

Reporting Standards

Does the manuscript adhere to the journal's guidelines on minimum standards of reporting? Choose an item.

Choose an item.

Statistics

Are you able to assess all statistics in the manuscript, including the appropriateness of statistical tests used? Choose an item.

Quality of Written English

Please indicate the quality of language in the manuscript: Choose an item.

Declaration of Competing Interests

Please complete a declaration of competing interests, considering the following questions:

- Have you in the past five years received reimbursements, fees, funding, or salary from an organisation that may in any way gain or lose financially from the publication of this manuscript, either now or in the future?
- Do you hold any stocks or shares in an organisation that may in any way gain or lose financially from the publication of this manuscript, either now or in the future?
- Do you hold or are you currently applying for any patents relating to the content of the manuscript?
- Have you received reimbursements, fees, funding, or salary from an organization that holds or has applied for patents relating to the content of the manuscript?
- Do you have any other financial competing interests?
- Do you have any non-financial competing interests in relation to this paper?

If you can answer no to all of the above, write 'I declare that I have no competing interests' below. If your reply is yes to any, please give details below.

I declare that I have no competing interests

I agree to the open peer review policy of the journal. I understand that my name will be included on my report to the authors and, if the manuscript is accepted for publication, my named report including any attachments I upload will be posted on the website along with the authors' responses. I agree for my report to be made available under an Open Access Creative Commons CC-BY license (http://creativecommons.org/licenses/by/4.0/). I understand that any comments which I do not wish to be included in my named report can be included as confidential comments to the editors, which will not be published.

Choose an item.

To further support our reviewers, we have joined with Publons, where you can gain additional credit to further highlight your hard work (see: https://publons.com/journal/530/gigascience). On publication of this paper, your review will be automatically added to Publons, you can then choose whether or not to claim your Publons credit. I understand this statement.

Yes Choose an item.
